# Supplementary material for: Teaming up main group metals with metallic iron to boost hydrogenation catalysis
Source: Nat Commun. 2022 Jun 9;13:3210. doi: 10.1038/s41467-022-30840-4 (PMC9184469; doi:10.1038/s41467-022-30840-4)
Supplement: Supplementary file 1 — Supplementary Information [file 41467_2022_30840_MOESM1_ESM.pdf]

Supplementary Information: Teaming up main group metals with metallic iron to boost hydrogenation catalysis

S. Harder et al.

Contents:

1. General experimental conditions S2

2. Metal activation by Metal Vapor Synthesis (MVS) S3

3. Solid state investigation on Ba^0, Fe^0 and the grinded BaFe mixture S6

4. Catalytic hydrogenation of alkenes, arenes, alkynes and imines S15

5. Investigations towards the mechanism and the nature of the BaFe catalyst S27

6. Monitoring of hydrogenation catalysis by ^{1}H NMR S34

References S63

1. General experimental conditions

All experiments were conducted in dry glassware or stainless steel reactors under an inert nitrogen atmosphere by applying standard Schlenk techniques or gloveboxes (MBraun). Deuterated benzene (C_6D_6, 99.6%D, Sigma-Aldrich) was degassed, dried over activated molecular sieves (3 Å) and stored under an inert atmosphere. $CDCl_3$ (99.8%D, Sigma-Aldrich) was used as obtained. The solvents benzene (Carl Roth GmbH, 99.5%) and toluene (Carl Roth GmbH, ≥99.5%) were degassed and dried over a column with activated aluminum oxide in a Solvent Purification System (Innovative Technology, Pure Solv 400-4-MD) and then stored under inert atmosphere over molecular sieves (3 Å). Following reagents were commercially available and, if not specified otherwise, were purified according to following standard steps. Liquids indicated by "*l*" were stirred over CaH_2 for 24 h and subsequently distilled and stored under nitrogen over molecular sieves (3 Å). Solids indicated by "*s*" were sublimed and stored under nitrogen in a glovebox. Hexamethyldisilazane (ABCR, 99.5%; no further purification; degassed and stored over molecular sieves, 3 Å), *n*-heptane (Sigma-Aldrich, 99%; no further purification; degassed and stored over molecular sieves, 3 Å), 1-hexene (Acros Organics, 97%; *l*), cyclohexene (Fluka, 99%; *l*), *cis*-3-hexene (TCI Chemicals, 97%; *l*), *trans*-3-hexene (TCI Chemicals, 97%; *l*), 1,2-diphenylethylene (Sigma-Aldrich, 97%; *l*), 1-methyl-cyclohexene (Alfa Aesar, 96%; *l*), triphenylethylene (Alfa Aesar, 98%; *s*), tetraphenylethylene (Sigma-Aldrich, 98%; *s*), *p*-xylene (Acros Organics, 99%; no further purification; degassed and stored over molecular sieves, 3 Å), anthracene (Sigma-Aldrich, 97%; *s*), naphthalene (Sigma-Aldrich, 99%; *s*), biphenyl (Sigma-Aldrich, >99%; *s*), acenaphthylene (TCI Chemicals, >94%; *s*), diphenylacetylene (Sigma-Aldrich, 98%; %; no further purification; degassed and stored over molecular sieves, 3 Å), 3-hexyne (Sigma-Aldrich, 99%; *l*), N-benzylidene-*tert*-butyl-imine (Sigma-Aldrich, 98%; *l*), quinoline (TCI Chemicals, >97%; *l*), 1-phenylcyclohexene (ABCR, 95%; *l*) and tetramethylethylene (Alfa Aesar, 97%, *l*). The imine $Ph_2C{=}NPh$ was synthesized according to a literature procedure.[1] Alkaline earth metals were obtained from ABCR: Ba (rod under mineral oil, 99+%), Sr (distilled dendritic pieces, 99.8%), Ca (turnings, 98%), Mg (turnings, 99.98%). Iron was obtained in the form of commercially available 5 inch nails. Prior to use, the larger pieces of metal were cut in smaller chunks (2-3 mm) with a heavy duty wire cutter. NMR spectra were measured on Bruker Avance III HD 400 MHz and Bruker Avance III HD 600 MHz NMR spectrometers. Chemical shifts (δ) are denoted in ppm (parts per million) and coupling constants in Hz (Hertz). ^{1}H and ^{13}C NMR spectra were referenced to the solvent residual signal ($SiMe_4$ = 0 ppm). Signal multiplicities are described using common abbreviations: s (singlet), d (doublet), t (triplet), q (quartet), quint (quintet), m (multiplet) and br (broad). CHN Elemental analysis was performed with a Hekatech Eurovector EA3000 analyzer or commissioned to an external company: Mikroanalytisches Laboratorium Kolbe, Oberhausen,

Germany. Quantitative CHN and metal analyses of the Fe^0 and Ba^0 samples obtained by cocondensation was commissioned to Mikroanalytisches Laboratorium Kolbe. GC/MS measurements were performed with a Thermo Scientific™ Trace™ 1310 gas chromatography system (carrier gas helium) with detection by a Thermo Scientic™ ISQ™ LT Single Quadrupole mass spectrometer. A Phenomenex® ZebronTMZB-5 column of the dimensions 0.25mm x 30m with a film thickness of 0.25 µm was used. The samples (1 µl) were injected with an Instant Connect-SSL Module in the split mode (Injector Temperature: 280 °C, split ratio 0.9, carrier gas flow 1.2 mL/min). Conditions for mass spectrometry: ion source temperature 280 °C, ionizing energy (70 eV), mass range 20-500 (m/z). The molecular identity was confirmed by comparison with entries in the NIST/EPA/NIH mass spectral library (version 2.2, built June 10 2014).

The particle morphologies of powdered Fe^0, Ba^0 and a spent FeBa catalyst were analyzed by scanning electron microscopy (SEM) using an Apreo S Lovac microscope and by transmission electron microscopy (TEM) studies using an Jeol JEM 2200 fs microscope equipped with probe-side Cs-corrector operated at 200 kV acceleration voltage. Samples were inserted with an Air-Free Transfer TEM holder. X-ray diffraction (XRD) patterns were collected with a Stoe STADI P diffractometer with Mo $K_{\alpha 1}$ radiation (λ: 0.7093 Å, 40 kV, 40 mA). The powdered catalysts were measured in sealed capillaries (Ø 500 µm) to prevent oxidation. X-ray photoelectron spectroscopy (XPS) was performed using a Versaprobe II™ from Ulvac-Phi with monochromatic Al K_{α} light at 1486.6 eV photon energy and an 45° emission angle between analyser and sample.

All hydrogenation experiments were carried out in stainless steel high-pressure autoclaves (volume: 15 mL) made by Advanced Machinery and Technology Chemnitz GmbH (Amtech). Autoclaves and stirring bars have never been in contact with transition metal catalysts. Autoclaves were only cleaned with dilute solutions of acetic acid to avoid metal abrasion and were dried prior to use by heating in an oven at 80 °C overnight. The reactors were connected by flexible high pressure tubing to a high pressure device consisting of a pressure regulator (1-50 bars) and a metering valve and pressurized with hydrogen (H_2, Air Liquide, 5N-purity: 99.999%).

2. Metal activation by Metal Vapor Synthesis (MVS)

General considerations:

CAUTION: All activated metal powders are extremely pyrophoric and burn vigorously in air.

The metals and a volatile organic matrix were evaporated under high vacuum and cocondensed together on a glass surface which was cooled to the temperature of liquid nitrogen. The home-build reactor follows the principle of a common oil diffusion pump with backing pump and cold trap operating under high vacuum in the range of 10^{-4} to 10^{-6} mbar.[2,3] The heating unit encloses an aluminum oxide container for the metal to be evaporated. The organic matrix, which cocondensed with the metal, was introduced through manifolds. In a typical Metal Vapor Synthesis (MVS) experiment, the metal (ca. 1.5-5.0 g) was cut into pieces of circa 5 mm which were placed in the aluminum oxide crucible and the reactor was evacuated to ca. 10^{-5} mbar. The crucible containing the metal was heated up to ca. 250 °C for several hours. During this process all volatiles, including humidity on the glass surface, were removed and collected in the cold trap. After previous cooling of the apparatus to room temperature, the reaction flask was cooled further with liquid nitrogen. Subsequently, ca. 10 mL of organic matrix was introduced through the manifold by evaporation. The matrix condensed on the cold glass surface and formed a thin layer of frozen organic solvent on the glass wall. This organic layer acts as a spacer between the flask and cocondensed metal and assists in draining and isolation of the metallic slurry. Subsequently, the metal in the aluminum oxide crucible was heated to the temperature needed for metal vaporization while the organic matrix (ca. 140 mL) was continuously introduced. The desired onset temperature was reached when metal condensation on the glass walls started, as indicated by formation of vividly colored cocondensates of finely divided metal. In case of alkaline-earth metals, often blue or yellow/green colors appeared which later turned black or brown, depending on the solvent. At the onset of metal evaporation, the crucible temperature was measured by an internal heat sensor. The onset temperature is generally equal to the melting point of the metal. To maintain a significant metal vapor flow, the temperature during the cocondensation experiment was adjusted circa 100 °C higher than the onset temperature. Typical values are dependent on the machine and the quality of the vacuum but a guideline for the operation temperature can be found below for the individual metals. The cocondensation process was stopped after all solvent (matrix) had been cocondensed into the reaction flask (ca. 1 h). After completion of metal cocondensation, the cooling bath under the condensation flask was removed, the reactor was filled with nitrogen gas and was allowed to warm up to room temperature. The metal suspension that drains to the bottom of the flask can be easily isolated by transferring it into a syringe with a long stainless steel cannula. The metal suspension was collected in a centrifuge Schlenk tube and after centrifugation and removing of the supernatant, the remaining metal powder was dried in vacuum at room temperature and stored in a glovebox. Generally, ca. 70% of the initially introduced metal could be isolated in the form of highly pyrophoric activated metal powder.

Activated alkaline-earth metals Ba, Sr, Ca and Mg: An aluminium oxide crucible was completely filled with alkaline earth metal pieces. Typical amounts used: Ba ca. 4.0 g, Sr ca. 2.0 g, Ca ca. 1.5 g and Mg ca. 2.0 g. MVS was carried out as described above at a pressure of circa 10^{-5} mbar and at temperatures that are circa 100 °C higher than the metal melting points; melting points: Mg 650 °C, Ca 842 °C, Sr 777 °C, Ba 727°C. For the metals Ba, Sr and Ca, *n*-heptane was used as the organic matrix. Cocondensation of Mg and *n*-heptane gave larger unreactive Mg lumps, however, using THF as a matrix led to a highly reactive Mg powder. Generally, ca. 70% of the initially introduced metal could be isolated in the form of highly pyrophoric activated metal powder. The activated Ba metal powder that was obtained by this method was analyzed for metal content. Elemental analysis (w%): Ba 93.72, C 1.38, H 0.34, N 3.24. The high N value is due to adsorption of N_2 on the Ba^0 surface.

Activated iron:

Iron was activated by cocondensation with an organic solvent at a vacuum of 10^{-5} mbar and an operation temperature that is circa 100 °C higher than the melting point of Fe (mp: 1535 °C). When *n*-heptane was used, the obtained iron powder formed an agglomerate which could not be removed by cannula transfer from the reaction vessel, presumably due to the magnetic nature of the material. However, using toluene during the cocondensation process a red solution of $Fe(toluene)_2$ was obtained. This labile complex[4] decomposed above ca. -60°C to a fine slurry of metallic Fe^0 which after standing for two days separated from the organic fraction.

Although not strictly necessarily, it was found that addition of hexamethyldisilazane, $HN(SiMe_3)_2$, during the cocondensation process gave an increased yield of activated Fe^0. Optimization led to the following method: Iron chunks (ca. 5.0 g) were cocondensed in a manner as described above for the alkaline-earth metals but the organic matrix was replaced by toluene (150 mL) and hexamethyldisilazane ($HN(SiMe_3)_2$, 20 mL) and at a vacuum of 10^{-5} mbar the operation temperature was circa 1635 °C (be aware that this is machine and vacuum dependent). The metal powder was separated from the mother liquor by decantation, washed several times with 10 mL portions of toluene and after isolation dried in vacuum at room temperature. The activated Fe powder was stored under N_2 in a glove box at room temperature. Yield: 3.20 g Fe in the form of a pitch-black, highly pyrophoric powder. Elemental analysis (w%): Fe 91.37, C 2.89, H 0.66, N 0.45.

3. Solid state investigation on Ba^0, Fe^0 and the ground BaFe mixture

During our investigations on alkene hydrogenation with Ba^0/Fe^0 mixtures, it was found that grinding the two metal powders with mortar and pestle is advantageous for the catalytic activity. Activated Ba^0, Fe^0 and the ground BaFe mixture have been characterized by powder X-ray diffraction (p-XRD), transmission electron microscopy (TEM), scanning electron microscopy (SEM) and X-ray photoelectron spectroscopy (XPS). The quality of the TEM measurements was negatively affected by problems to disperse the samples using sonication. The SEM and XPS measurements were affected by problems to insert the samples in the machine under inert conditions.

The p-XRD data showed that the Fe^0, Ba^0 and BaFe catalysts are crystalline nanosized metallic powders with particle diameters of circa 5 nm. According to XPS measurements, the surfaces not only consist of metallic Fe^0 and Ba^0 but are partially oxidized which is related to their highly reactive pyrophoric nature. The metal samples were stored in closed ampoules under high vacuum and the XPS samples were freshly prepared in a glovebox under inert gas conditions (Ar, < 0.01 ppm O_2). However, the reactivity of these nanostructured catalysts is sufficiently high that their surfaces already decomposed by traces of O_2 and H_2O during sample insertion. The color of these partial decomposed samples remained pitch-black indicating that only the surface was partially oxidized due to sample insertion (Ba^0 should become white upon oxidation whereas Fe^0 would turn brown).

Figure S1 shows the powder X-ray diffractograms (p-XRD) for the Ba^0, Fe^0 and the ground BaFe catalyst. These samples were measured in sealed capillaries which were filled in the inert atmosphere of a glovebox (Ar, < 0.01 ppm O_2) and therefore these spectra are not affected by partial surface oxidation. Only Bragg reflections corresponding to the metallic phases of Ba^0 and Fe^0 are visible, while reflexes due to the presence of other crystalline phases including metal oxides or hydroxides, were not observed. Although metallic barium usually crystallizes in a body-centered cubic lattice (α-Ba), the observed p-XRD pattern is in good agreement with the reported pattern for its β-modification which crystallizes in a face-centered cubic lattice. The β-modification of Ba^0 was first observed for barium particles obtained by metal evaporation and spraying into high vacuum,[5] *i.e.* conditions similar to those used for the preparation of the activated Ba^0 catalyst. Based on the peak width, the particle size was calculated to ca. 5 nm for both the Ba^0 and the Fe^0 catalysts.

SEM photographs of the Fe^0 and Ba^0 catalysts are displayed in Fig. S2. These show that the nanoparticles have a high tendency to agglomerate, resulting in the formation of large particle clusters with roughly 100 nm in size. These clusters are composed of much smaller, uniform particles. The acquisition of high-

resolution images is made difficult caused by the partial oxidation of the extremely reactive particles as a result of the short exposure to traces of air during transfer into the SEM sample chamber.

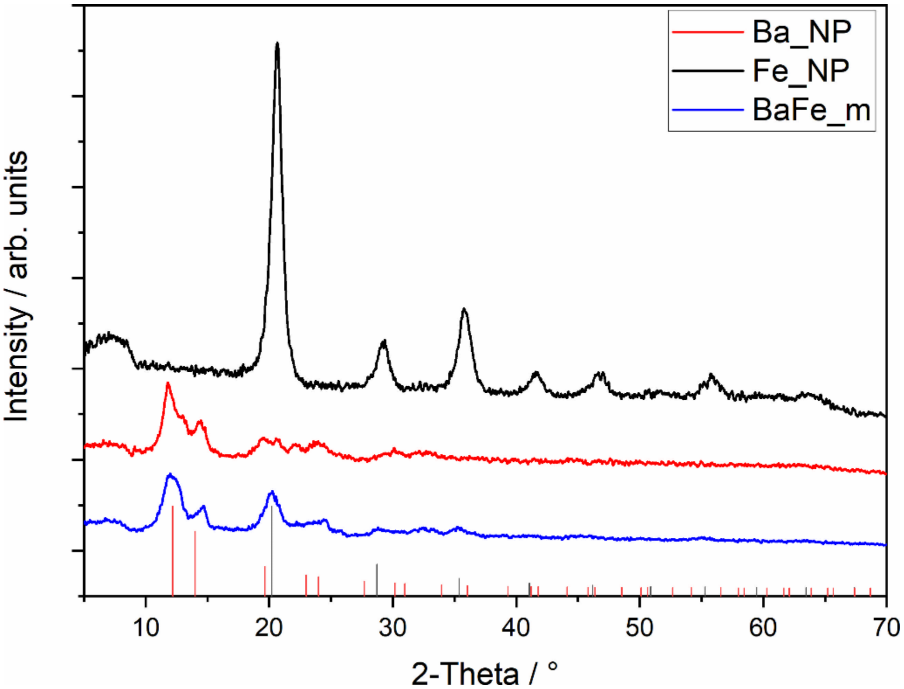

Figure S1. p-XRD spectra for activated Ba^0, Fe^0 and BaFe. The vertical lines at the bottom show the reference positions for elemental iron (black, ICSD#52258) and elemental barium (red, ICSD#52679).

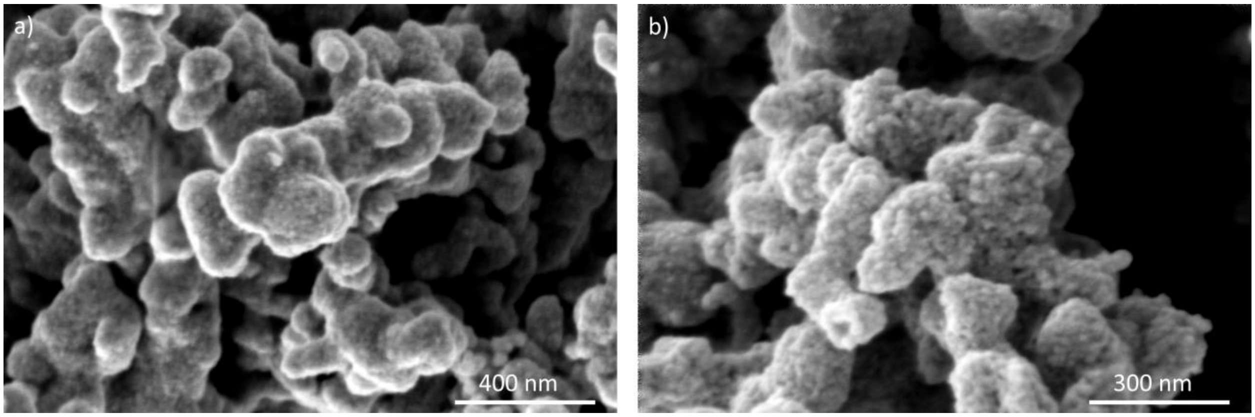

Figure S2. SEM photographs for the activated a) Ba^0 and b) Fe^0 nanoparticle agglomerates (partially oxidized at the surface).

TEM photographs of Fe^0, Ba^0 and a spent BaFe catalyst are displayed in Fig. S3. As the samples were inserted in the machine using an Air-Free Transfer TEM holder, surface oxidation was only minimal. The

TEM photographs show the formation of large particle agglomerates which consist of very small, uniform sub-10 nm particles. The high agglomeration tendency is due to the specific synthetic method which avoids use of sterically demanding organic molecules (capping agents) on the particle surface and complicates the acquisition of high-resolution images. Further dispersion of these particles by sonication was not possible.

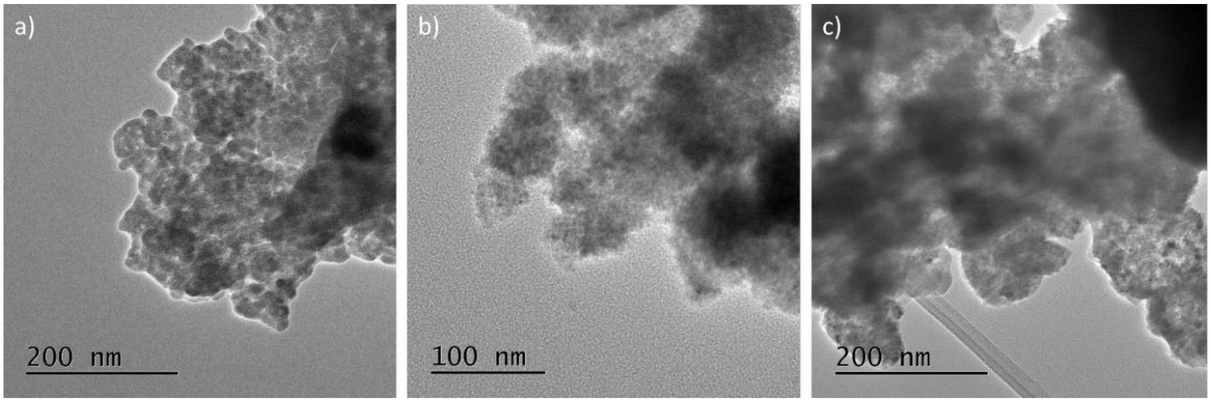

Figure S3. TEM photographs for the activated a) Ba^0, b) Fe^0 and c) a spent BaFe catalyst.

For further insight into the elemental composition and the valence states of the elements on the surface, X-ray photoelectron spectroscopy (XPS) was performed in the SEM. The XP survey spectra shown in Fig. S4 confirm the presence of the respective metals in addition to carbon and oxygen for the Ba^0 and Fe^0 catalysts, respectively. The Ba^0 catalyst also shows adsorbed N_2 which is typical for this metal. The XP spectrum for the ground BaFe catalyst is merely a superposition of Ba^0 and Fe^0 spectra. This is in agreement with the observation that Ba and Fe do not form alloys.[6]

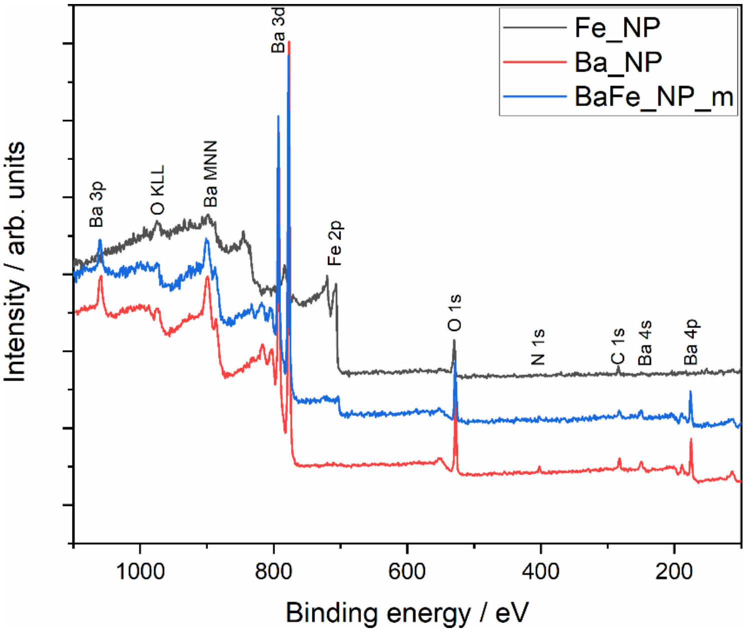

Figure S4. Survey XPS for the activated Ba^0, Fe^0 and BaFe catalysts.

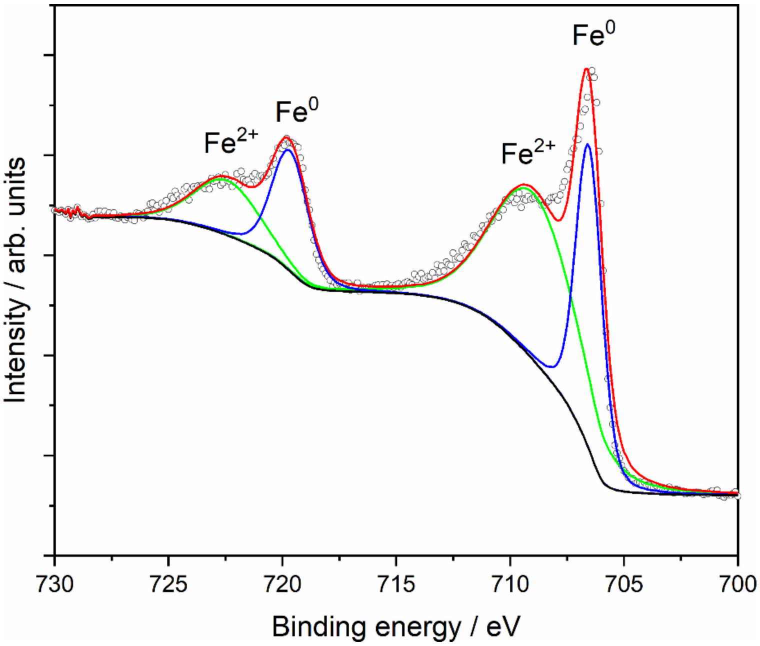

Figure S5. High-resolution XP spectrum of the Fe 2*p* region for the Fe^0 catalyst.

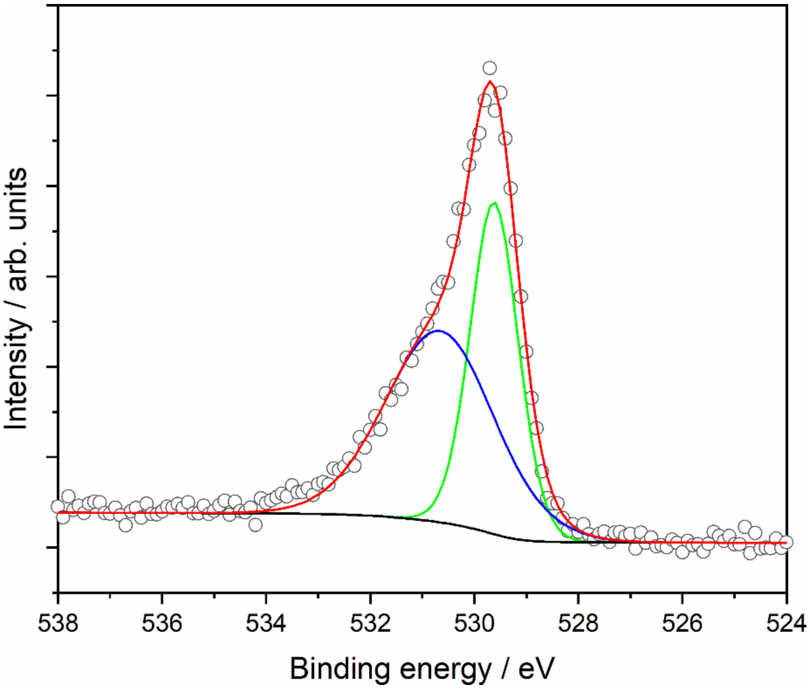

Figure S6. High-resolution XP spectrum of the O 1*s* region for the Fe^0 catalyst.

The high-resolution spectrum for the Fe 2*p* region for the Fe^0 catalyst (Fig. S5) shows two peaks for Fe $2p_{3/2}$ and Fe $2p_{1/2}$ at 706.7 and 719.7 eV, respectively, corresponding to metallic iron.[7] A second set of peaks at higher binding energies is visible at 709.5 eV and 722.7 eV. These values are in good agreement for the reported values for oxidized iron (Fe^{2+}). The ratio between metallic and oxidized Fe species is roughly 1:1.3. The high-resolution O 1*s* spectrum (Fig. S6) can be deconvoluted into two peaks, whereas the main peak at 529.6 eV corresponds to oxygen in the lattice of iron oxides and the minor peak at 530.7 eV to iron hydroxides.

The high-resolution spectrum for the Ba 3*d* region for the Ba^0 catalyst (Fig. S7) shows two peaks for Ba $3d_{5/2}$ and Ba $3d_{3/2}$ at 780.6 and 795.9 eV, corresponding to metallic barium, while a second set of signals at lower binding energies (777.7 and 792.8 eV) are in good agreement with reported values for Ba in the oxidation state +II, suggesting the presence of BaO. The ratio between metallic and oxidized Ba species is about 1:1.1. The high-resolution O 1*s* spectrum (Fig. S8) can be deconvoluted into two peaks, whereas the main peak at 528.5 eV corresponds to oxygen in metal oxide and the minor peak at 531.6 eV to barium hydroxide.[8]

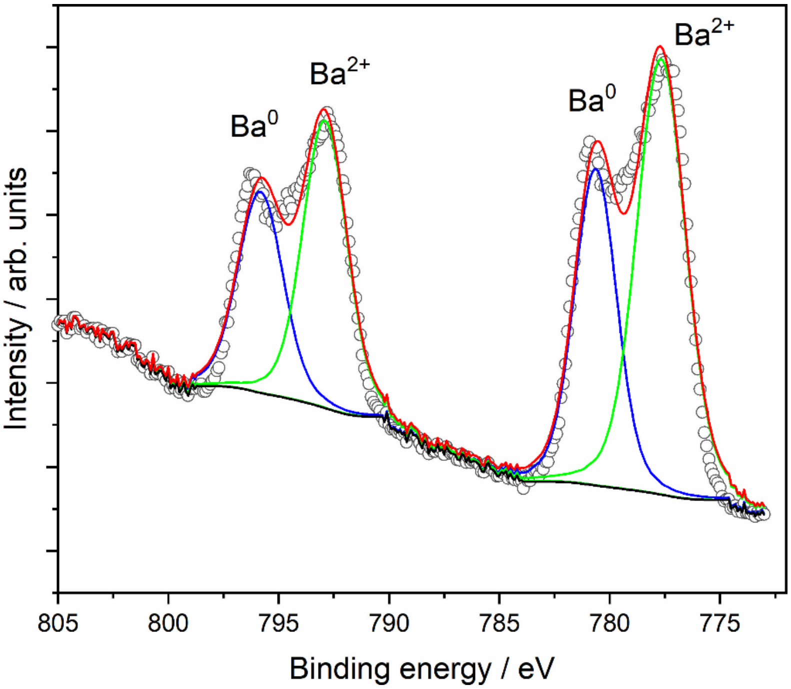

Figure S7. High-resolution XP spectrum of the Ba 3*d* region for the Ba^0 catalyst.

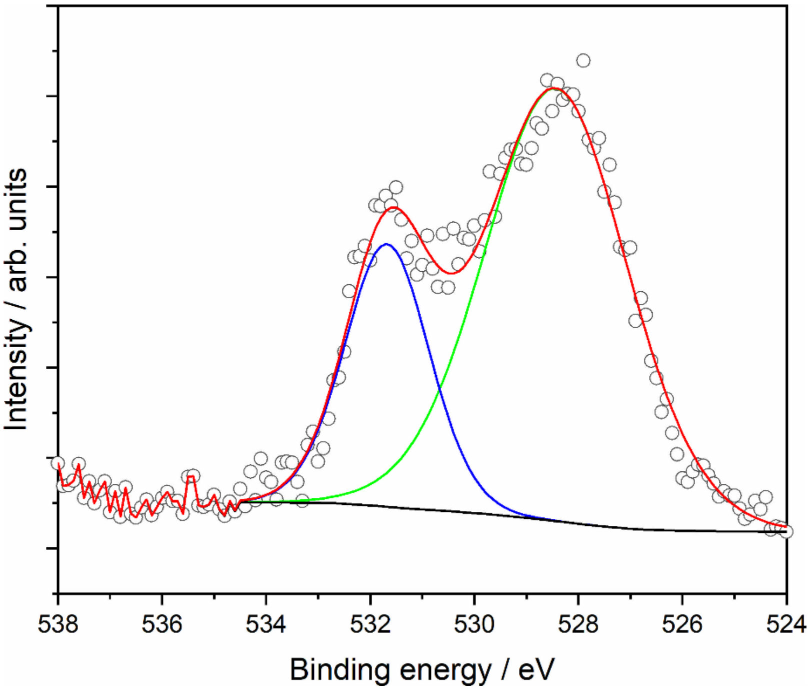

Figure S8. High-resolution XP spectrum of the O 1*s* region for the Ba^0 catalyst.

The spatial distribution of the elements Ba and Fe within the BaFe catalyst powder was determined by EDX mapping. Figure S9 shows the SEM photograph of a larger area of the native powder and the corresponding elemental mappings. Since the sample could not be introduced in the machine under purely inert conditions, partial oxidation/hydrolysis of the surface is to be expected but this does not influence the Ba/Fe distribution. It can be clearly seen that the elements barium (red) and iron (green) are spatially separated and present in micrometer sized areas. This distribution is due to the production method in which activated powders of pure Ba and Fe are mixed with a mortar and pestle. The composite image shows that the Ba-rich and the Fe-rich regions are complementary to each other.

The elemental composition of the catalyst in the native state was determined by EDX measurement (Fig. S10). This technique determines the nature and quantity of elements at the surface of the material (up to a depth of circa 1 μm). Due to partial oxidation of the surface, not only Ba and Fe but also O is present but this does not affect the Ba/Fe ratio. As the distribution of Ba and Fe in the native BaFe catalyst is rather inhomogeneous, the Ba/Fe ratio was determined by EDX for two larger areas of circa 200 x 200 μm (Table S1). The results of the two measurements are in good agreement and show an equimolar Ba/Fe ratio which corresponds to the 1/1 Ba/Fe ratio used for its preparation.

Also the spent catalyst (after hydrogenation of benzene at 150 °C and 50 bar H_2 pressure) was analysed by EDX mapping. Figure S11 shows that there is no significant change in the spatial distribution of Ba and Fe. It is, however, clear that the surface of the catalyst after use is much richer in Ba than in Fe. This was confirmed by EDX mapping in four different areas of circa 200 x 200 μm (Table S2). Although this showed a strongly heterogeneous Ba/Fe distribution, it is clear that the surface of the catalyst is enriched in Ba. Instead of the expected Ba/Fe ratio of 1/1 for the native catalyst, a Ba/Fe ratio of circa 2/1 is observed. Since the catalyst is fully recovered from the reaction mixture (see Figure S16), the enrichment of Ba in the surface area can only be explained by the proposed mechanism in which Ba^0 enters solution in the form of a homogeneous component which at the end of the catalytic reaction precipitates again in the form of Ba^0 or BaH_2.

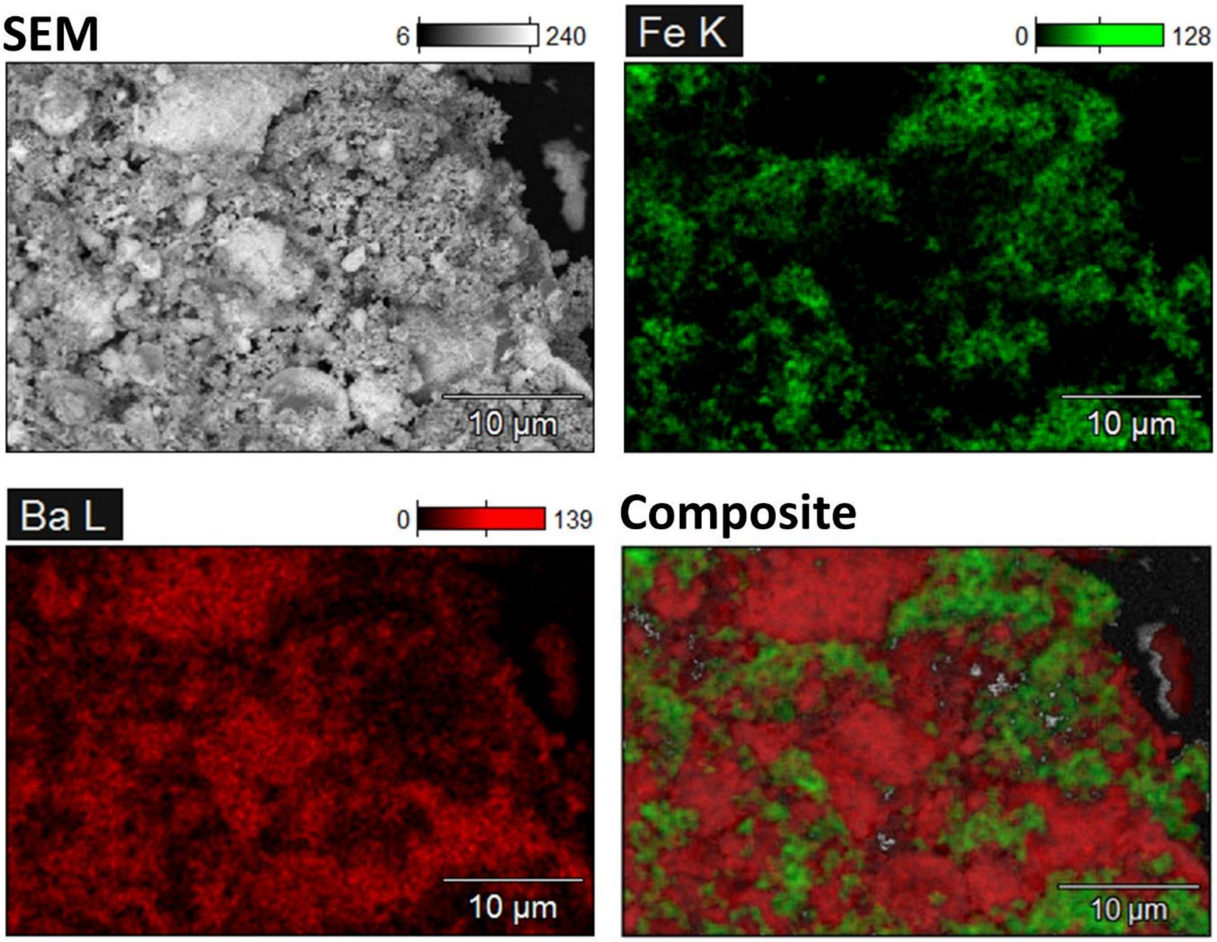

Figure S9. SEM photograph of the native BaFe catalyst and corresponding EDX elemental mapping of the elements Ba (red) and Fe (green).

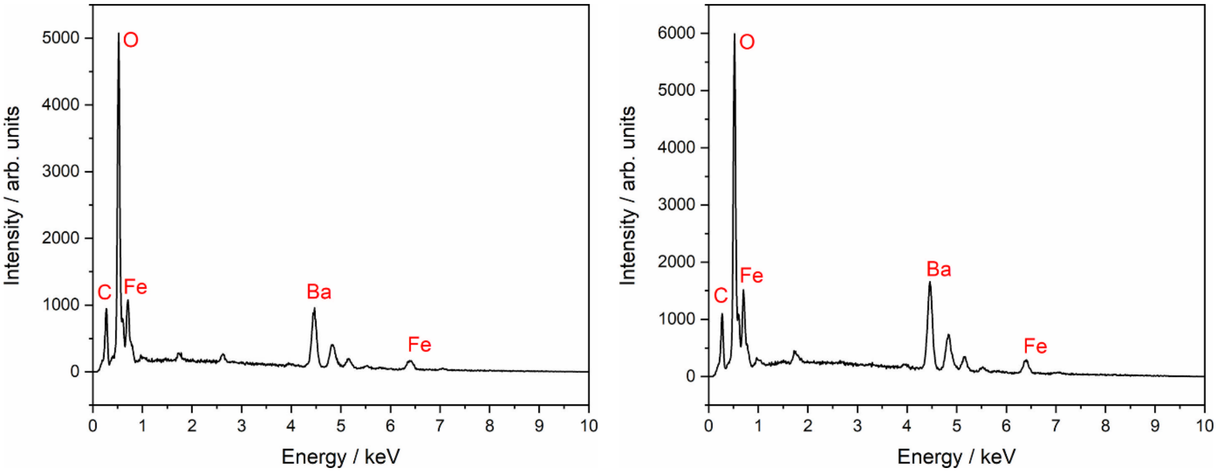

Figure S10. EDX spectrum of native (left) BaFe nanoparticles and after catalysis (right). The carbon signal is caused by the use of carbon adhesive tape for fixation of the powder on the sample holder.

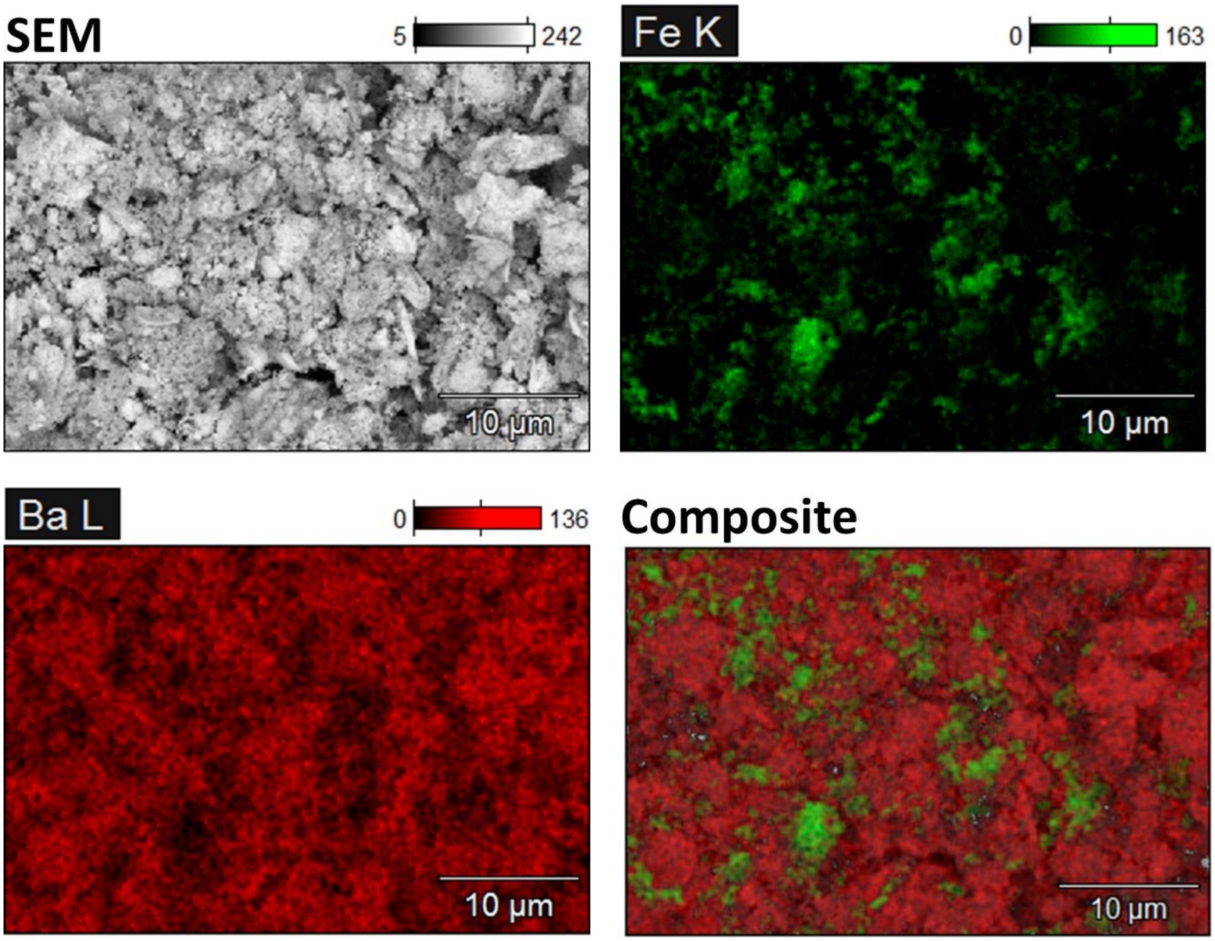

Figure S11. SEM photograph of BaFe nanoparticles after catalysis and corresponding EDS elemental mapping of the elements Ba(red) and Fe (green).

Table S1. Elemental composition of the surface of the native BaFe catalyst determined by EDX.

**Area**	**Ba at-%**	**Fe at-%**
1	48.6	51.4
2	49.5	50.5
Average	49.1	50.9

Table S2. Elemental composition of the surface of the spent BaFe catalyst determined by EDX.

Area	Ba at-%	Fe at-%
1	70.4	29.6
2	67.4	32.6
3	75.9	24.1
4	55.3	44.7
Average	67.3	32.7

4. Catalytic hydrogenation of alkenes, arenes, alkynes and imines

(1) General method for catalytic hydrogenation: A 15 ml autoclave equipped with a magnetic stir bar was stored overnight at 80 °C in a drying cabinet and subsequently transferred to the inert atmosphere of a nitrogen-filled glovebox. The desired amount of metal catalyst was weighed into the autoclave and subsequently the substrate was added. In case of the BaFe catalyst, the two powders were premixed by mortar and pestle. This was done on a large scale (few grams) in the inert atmosphere of a glovebox.

In case of liquid substrates, the catalytic reactions were conducted neat in 500 µL of the substrate (the amount of catalyst was calculated based on the desired mol%). Runs with very low catalyst loadings were conducted on a larger scale (small scale reactions would lead to inaccuracies in catalyst addition). For example: 1-hexene (2000 µL) with 0.05 mol% BaFe (1.5 mg) or cyclohexene (750 µL) with 0.33 mol% BaFe (4.7 mg). In hydrogenations of benzene a substrate volume of 750 µL was used for direct NMR analysis. This is the volume needed to fill a 5 mm NMR tube and the total reactor contents were, after filtering in order to remove paramagnetic Fe, transferred to an NMR tube for direct no-D NMR measurement (measurements without deuterated solvents and lock; internal spectrometer frequency was used for referencing). Solid substrates were first dissolved in *n*-heptane and added to the reactor as a solution.

After loading the reactors in a glovebox (N_2, <0.01 ppm O_2), they were and transferred outside to be pressurized with hydrogen to the specified pressure. After closing the gas inlet, the autoclaves were then placed into preheated aluminum blocks on magnetic stirrers and quickly reached the desired temperature. After a given time, the reactor was cooled to room temperature and conversion was determined by means of ^{1}H NMR spectroscopy and GC-MS analysis. In experiments in which heptane was

used as a solvent (solid substrates), the heptane solvent was removed under high vacuum prior to analysis. In most cases, the experimental conditions, including the time, were optimized for full conversion (>99%). Conversion times < 1 h were optimized with an accuracy of circa 0.1 h. Conversion times < 5 h were optimized with accuracies of circa 0.25 h. Conversion times < 10 h were optimized with accuracies of circa 0.5 h. Longer reaction times are less accurate (ca 1 h accuracy). All results were verified by repeating the experiment at least three times.

The catalytic activity of the BaFe catalyst is compared to earlier reported results on the catalytic activity of Ba^0.[9] Since TOF values are instantaneous and strongly dependent on time and substrate conversion,[10] we aimed to determine TOF values for essentially full conversion (99%), using optimized times (*vide supra*). Since TOF values are also dependent on substrate concentration and temperature, we kept the reaction conditions equal. This approach gives reasonably accurate turn-over-frequencies (TOF's) which must be seen as minimal values. Since we assume that the Fe part of the catalyst is fully heterogeneous, only surface atoms can be active. For ideal spherical particles of 5 nm (estimated by p-XRD), the degree of dispersion (ratio of surface to total atoms) is circa 15-20%. This means that, calculated per surface atom, our catalysts are at least a factor 5-6 times more active.

Since the reactors are closed off from the H_2 source during the hydrogenation experiment, the given pressure relates to the starting pressure. The pressure drops during H_2 consumption but in all cases there was a residual H_2 pressure of at least 5 bar after full conversion. At low H_2 pressure, the quantity of H_2 in the reactor is not sufficient for full substrate conversion. Therefore, runs at low pressure were conducted with reactors in which the gas inlets were kept open to the H_2 source. This was only possible for substrates of which the boiling points are substantially higher than the reactor operating temperature.

(2) Catalytic activity of Fe^0: The catalytic activity of activated Fe^0 without any cocatalyst has been investigated (Table S3). While terminal alkenes and cyclic internal alkenes like cyclohexene can be fully converted, the Fe^0 catalyst is hardly active in reduction of internal linear alkenes (*e.g.* 3-hexene) and fully inactive in reduction of 1,1-diphenylethylene or tri-substituted alkenes (*e.g.* 1-phenylcyclohexene and 1-methylcyclohexene). As it also failed to reduce benzene, Ba^0 alone is clearly the better hydrogenation catalyst (numbers in Table S3 are taken from ref. [9]). Only for 1-hexene the Fe^0 catalyst was shown to be slightly more active than the Ba^0 catalyst.

Table S3. Comparison of catalytic hydrogenations with only Ba^0 or only Fe^0, both activated by cocondensation (MVS).

	Catalyst Ba^0						Catalyst Fe^0					
**substrate**	**mol %**	**H_2 [bar]**	**T [°C]**	**t [h]**	**conv. [%]**	**TOF $[h^1]$**	**mol %**	**H_2 [bar]**	**T [°C]**	**t [h]**	**conv. [%]**	**TOF $[h^1]$**
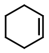	5	12	25	3	99 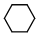	7	5	12	25	3	2 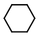	0.1
	5	12	80	0.5	99 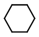	40	5	12	80	>1	15 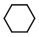	3
	2.5	12	100	1	99 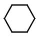	40	4	12	100	20	53 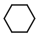	0.7
	-	-	-	-	-	-	2.5	50	150	4	99 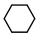	10
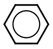	10	50	150	144	99 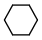	0.1	10	50	150	24	0	0
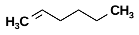	5	12	100	3	99 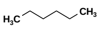	7	2.5	20	150	0.2	87/10 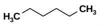 / 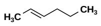	20
	2.5	12	30	1.25	1/99 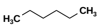 / 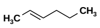	0.3	2.5	12	22	3	23/73 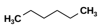 / 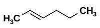	3.1
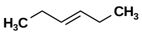	10	20	120	24	99 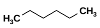	0.4	2.5	20	150	5	11 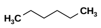	0.9
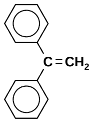	2.5	20	120	0.5	>99 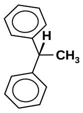	80	2.5	20	120	10	0	0
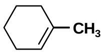	10	20	150	24	99 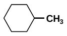	0.4	2.5	20	150	10	0	0

(3) Catalytic activity for BaFe at a constant low pressure of 6 bar: A metal mixture of Ba^0 and Fe^0 is also at low constant H_2 pressure active in hydrogenation catalysis (Table S4). For runs at higher temperature (120-150 °C) the reactor was pressurized and before heating shut off from the H_2 source in order to prevent boil-off of the substrate. This means that there is a natural pressure drop during hydrogenation. In hydrogenations at lower temperature the valve to the H_2 source could be kept open and consequently these were run at a constant H_2 pressure. Table S4 shows that alkene and arene hydrogenation with the BaFe catalyst do not necessarily need a high H_2 pressure but can also be run at a low constant H_2 pressure of 6 bar.

Table S4. Catalytic hydrogenation with BaFe at low temperature and a low (constant) pressure of 6 bar (in green) compared to closed reactors (H_2 source shut off) at higher variable pressure (in red); reaction times for full conversion have been optimized.

substrate	mol%	T [°C]	H_2 [bar]	t [h]	conv. [%]
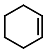	2.5	20	12 Closed reactor	0.5	99 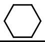
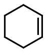	2.5	20	6 Constant pressure	0.5	89 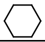
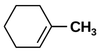	5	20	20 Closed reactor	7	99 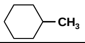
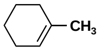	5	20	6 Constant pressure	7	99 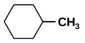
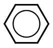	3	60	50 Closed reactor	4	84 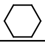
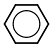	3	60	6 Constant pressure	4	94 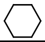

Not only the H_2 pressure but also the substrate concentration has no effect on the conversion rate: the hydrogenation of benzene to cyclohexane is essentially not affected by dilution with heptane (Table S5).

Table S5. Investigation of the substrate concentration dependency for the catalytic hydrogenation of benzene to cyclohexane with BaFe (1 mol%, 50 bar H_2, 120 °C, 1.5 h).

Substrate	Solvent	Conversion
0.75 ml benzene	neat	35%
0.75 ml benzene	0.75 ml heptane	32%
0.75 ml benzene	1.5 ml heptane	33%
0.75 ml benzene	2.25 ml heptane	33%

(4) Hydrogenation with X/Fe catalysts (X = molecular main group metal catalyst): Although the majority of our research is focussed on the BaFe catalyst system, it was noticed that the addition of finely divided Fe^0 also boosts the catalytic activity of molecular main group metal complexes (Table S6). Simple Ae metal amides like $Ae[N(SiMe_3)_2]_2$ (AeN''_2), which are precursors to soluble Ae metal hydride clusters, also cooperate with Fe^0. While MgN''_2 is fully inactive in alkene hydrogenation, the mixture with Fe^0 forms a highly active catalytic system which reduces even the most challenging alkenes like $Me_2C{=}CMe_2$. Also for the heavier BaN''_2, catalytic activity is enhanced by several orders of magnitude. Interestingly, commercially available BaH_2 (ABCR, 99.5%, 60 mesh) alone is not catalytically active as a hydrogenation catalyst, however, in combination with Fe^0 it becomes a potent reducing agent. The scope of molecular main group metal catalysts is not restricted to Ae metal complexes. The catalyst system LiN''/Fe is able to hydrogenate cyclohexene, a task which even with BaN''_2 alone could not be fulfilled. Table 1 in the manuscript and Table S6 summarize the results for catalytic hydrogenation of various substrates.

Table S6. Catalytic hydrogenations with different cooperative catalysts X/Fe^0. Reaction times for full conversion have not been optimized.

substrate	X/Fe^0	mol%	H_2 [bar]	T [°C]	t [h]	conv. [%]
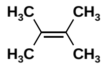	$Mg[N(SiMe_3)_2]_2$/Fe	3	50	150	6	>99 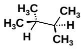
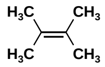	BaH_2/Fe	3.4	50	150	6	50 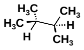
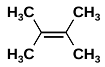	$Ba[N(SiMe_3)_2]_2$/Fe	3.4	50	150	6	93 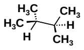
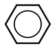	Mg(*n*-Bu)$_2$/Fe	3	50	150	0.5	27 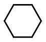
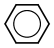	$Mg[N(SiMe_3)_2]_2$/Fe	1.5	50	150	2	33 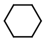
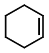	BaH_2/Fe	1.4	50	80	2	>99 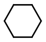
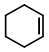	$Mg[N(SiMe_3)_2]_2$/Fe	1.5	50	80	2	>99 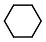
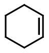	$LiN(SiMe_3)_2$/Fe	1.5	12	80	2	20 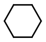
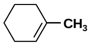	$Ba[N(SiMe_3)_2]_2$/Fe	3.4	50	150	4	>99 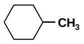

For accuracy the following typical quantities have been used for experiments in Table 1 (see manuscript):

Table 1. Catalytic benzene hydrogenation (benzene: 0.75 ml, 657 mg, 8.41 mmol) with Fe^0 (1.5 mol% = 0.126 mmol = 7.0 mg) and catalyst component X (1.5 mol%).[a] Conditions: 150 °C, 50 bar, 2 h.

X 1.5 mol%	X mg	conv. [%]	TOF [h^{-1}]
Ba^0	17.3	>99	>33
CaH_2	5.3	0	0
SrH_2	11.3	3	1
BaH_2	17.6	82	21
MgN''_2	43.5	32	11
CaN''_2	45.5	29	10
SrN''_2	51.5	40	13
BaN''_2	57.7	86	29
LiN''	21.1	2.5	1
NaN''	23.1	10	3
KN''	25.1	40	13
$[Ba(H)N'']_7$	37.6	21	7
$[(BDI)MgH]_2$	55.8	>99	>33

[a] For the aggregates $[Ba(H)N'']_7$ and $[(BDI)MgH]_2$ the 1.5 mol% X has been calculated based on the monomeric units Ba(H)N'' and (BDI)MgH.

Benzene hydrogenation with $[(BDI)MgH]_2/Fe^0$: The last experiment in Table 1 (manuscript and above) is an example in which a soluble, molecular Mg hydride catalysts cooperates with Fe^0. The bulky β-diketiminate (BDI) ligand in $[(BDI)MgH]_2$ contributes to its stability for ligand exchange reactions to $Mg(BDI)_2$ and insoluble MgH_2, and therefore keeps the Mg hydride in solution.

Attempts to hydrogenate benzene with $[(BDI)MgH]_2$ (1.5 mol%, 150 °C, 50 bar, 2 h) were unsuccessful. Cyclohexane could not be detected and the catalyst is unchanged (Figure S12). However, the addition of 1.5 mol% of MVS-activated Fe^0 led to full conversion to cyclohexane (Figure S13). The Fe^0 catalyst could be separated from the mother liquor with a magnet. The no-D ^1H NMR spectrum of the mother liquor

shows that complex [(BDI)MgH]$_2$ is unchanged (Figure S14), showing that the Fe^0 and [(BDI)MgH]$_2$ catalysts both work in synergy.

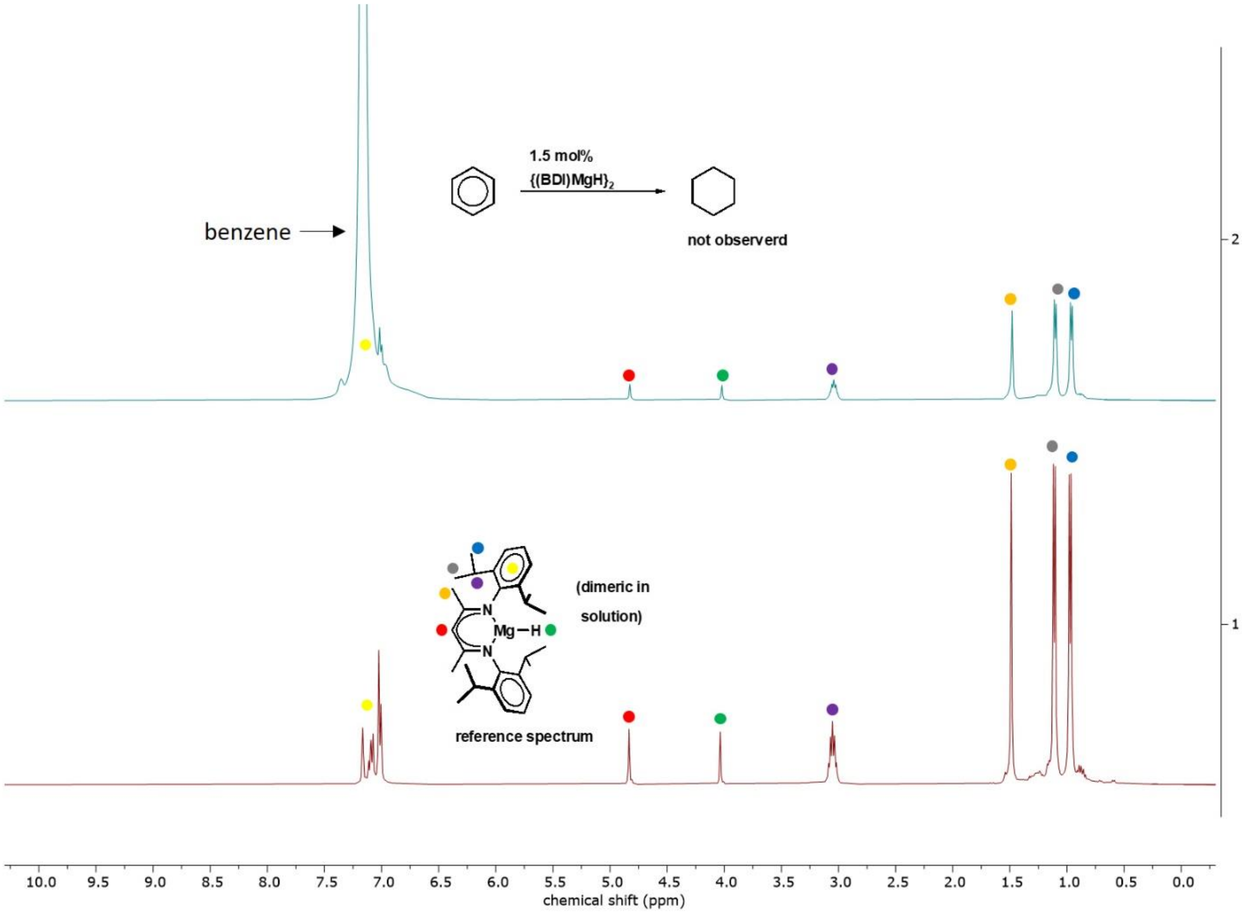

Figure S12. Top: no-D ^{1}H NMR spectra for the attempted conversion of benzene to cyclohexane using 1.5 mol% [(BDI)MgH]$_2$ as the catalyst showing 0% conversion (150 °C, 50 bar, 2 h). The catalyst is unchanged. Bottom: Reference ^{1}H NMR spectrum for [(BDI)MgH]$_2$ in C_6D_6 for comparison.

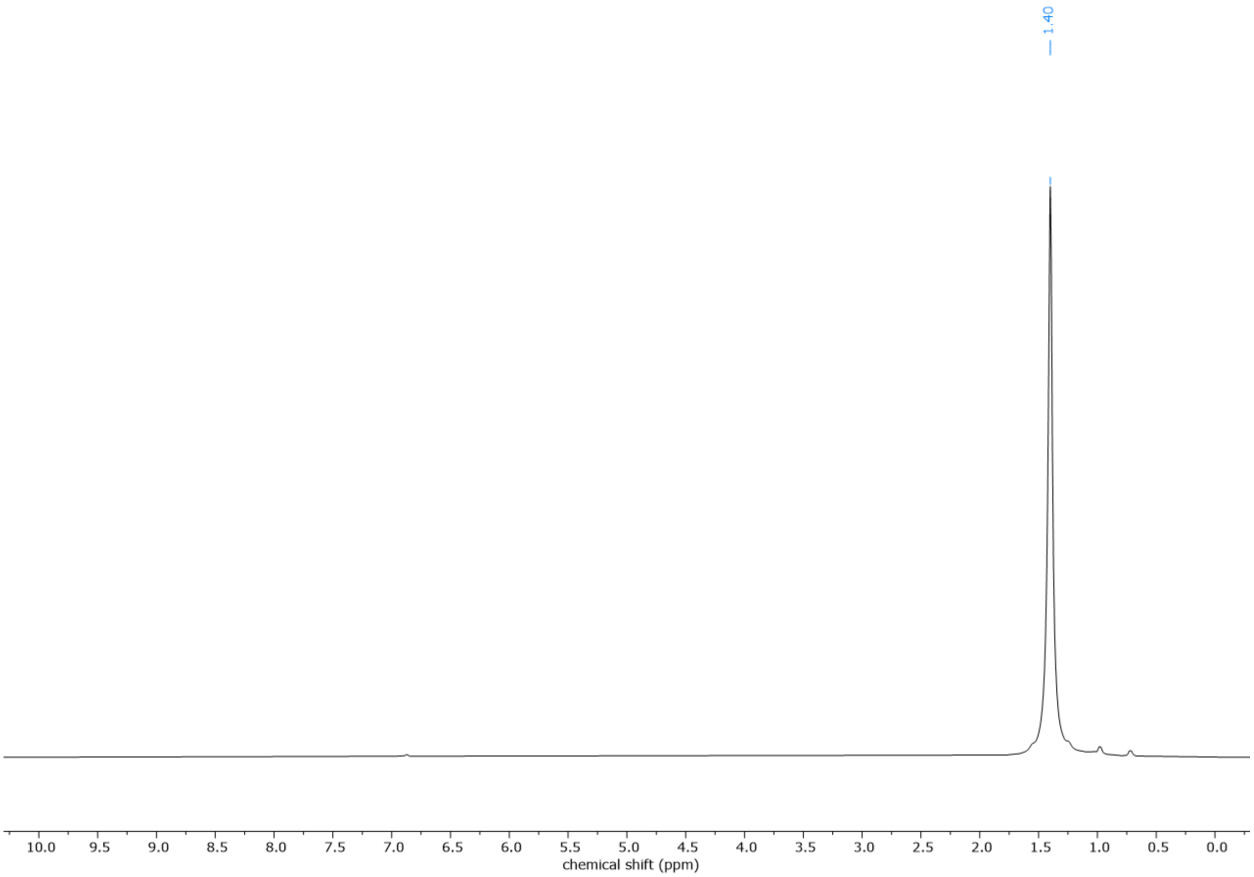

Figure S13. no-D ^{1}H NMR spectrum for the conversion of benzene to cyclohexane using 1.5 mol% of Fe^{0} and 1.5 mol% $[(BDI)MgH]_2$ as the catalyst showing full conversion (150 °C, 50 bar, 2 h).

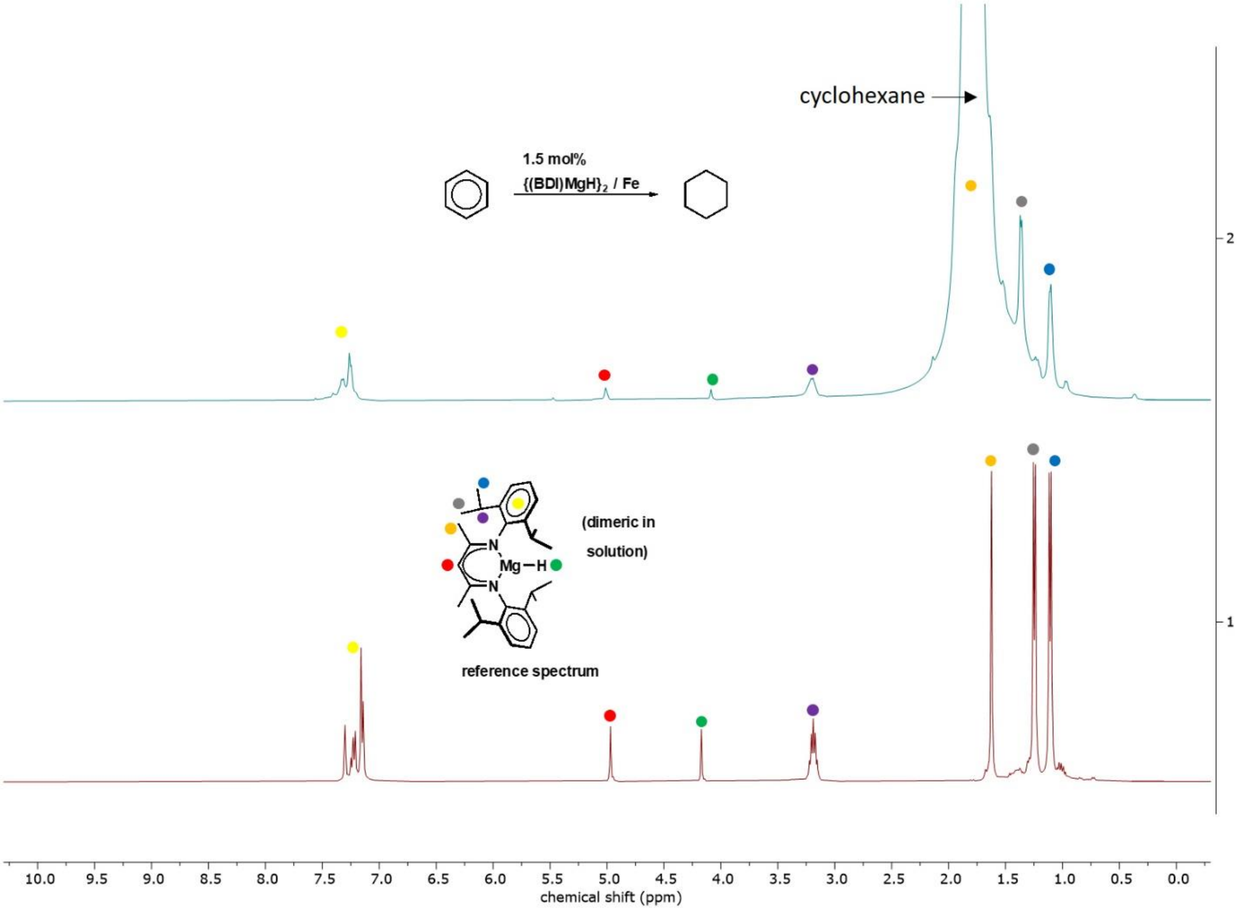

Figure S14. Top: Amplified no-D ^1H NMR spectrum for the conversion of benzene to cyclohexane using 1.5 mol% of Fe^0 and 1.5 mol% $[(BDI)MgH]_2$ as the catalyst. Apart from cyclohexane, small signals for $[(BDI)MgH]_2$ are visible in the baseline. These signals agree very well, but do not exactly coincide, with those for $[(BDI)MgH]_2$ in C_6D_6 (bottom). This is due to a change of solvent from benzene to cyclohexane.

(5) Hydrogenation with catalysts based on different Fe^0 sources: The importance of finely divided Fe^0 particles and clean unoxidized metal surfaces was evaluated using different Fe^0 cocatalysts (Table S7). Under no circumstances did we find any acceleration for the catalytic activity of Ba^0 by addition of commercially available Fe powder (stored under air). Pyrophoric Fe^0, obtained by thermal decomposition of iron oxalate under vacuum, is also an efficient cocatalyst for Ba^0. Commercially available Fe oxalate was decomposed under vacuum by heating a 25 ml Schlenk flask with 2.0 g of yellow Fe-oxalate·$(H_2O)_2$ with a Bunsen burner. At a temperature of circa 200 °C the water was released. The Schlenk was then further heated to circa 400 °C until CO_2 release stopped (after circa 10 minutes). Due to vigourous CO_2

evaporation, considerable quantities of pyrophoric iron are lost in the tubes of the vacuum line and the dust filter. The yield of pitchblack pyrophoric iron was circa 1.0 g. Since this is an approximately equimolar mixture of Fe^0 and Fe_3O_4 (with traces of FeO),[11] we used in catalysis experiments the double amount of pyrophoric iron. In all cases, the most active Fe cocatalyst was found to be Fe^0 powder obtained by MVS.

Table S7. Investigations towards the Fe source in BaFe catalysts. Fe^0(MVS) = iron activated by metal vapour synthesis. Fe^0(powder) = commercially obtained iron powder. Fe^0(oxalate) = iron obtained by thermal decomposition of iron oxalate (the double amount of Fe was used as cocatalyst).

Substrate	Catalyst	mol%	p H_2 [bar]	T [°C]	t [h]	conv. [%]	TOF [h^{-1}]
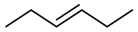 *trans*	Ba^0/Fe^0(MVS)	0.25	20	120	0.5	99	800
	Ba^0/Fe^0(powder)	3	20	120	5	0	0
	Ba^0/Fe^0(oxalate)	0.25	20	120	0.5	0	0
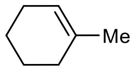	Ba^0/Fe^0(MVS)	0.5	20	150	0.5	99	400
	Ba^0/Fe^0(powder)	3	20	150	5	0	0
	Ba^0/Fe^0(oxalate)	0.5	20	150	0.5	33	132
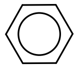	Ba^0/Fe^0(MVS)	3	50	150	0.5	99	67
	Ba^0/Fe^0(powder)	3	50	150	5	0	0
	Ba^0/Fe^0(oxalate)	3	50	150	0.5	67	14
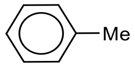	Ba^0/Fe^0(MVS)	3	50	150	3	99	11
	Ba^0/Fe^0(powder)	3	50	150	3	0	0
	Ba^0/Fe^0(pyrophoric)	3	50	150	3	16	2

(6) Optimization of the Ba/Fe ratio: The Ba^0 / Fe^0 ratio in the BaFe catalyst was optimized for its activity in benzene hydrogenation (Table S8). The activity increases linearly with Fe content but reaches its peak at an equimolar ratio (Fig. S15). As additional Fe hardly affects the activity, hydrogenation catalysis was performed with 1 / 1 mixtures.

Table S8. The effect of the Fe/Ba ratio on catalytic benzene-d_6 hydrogenation. Experimental details: 15 mL stainless steel autoclave, 0.75 mL C_6D_6, 50 bar H_2, 150 °C, 2h. Conversion determined by ^{2}D NMR.

mol% Ba	mol% Fe	Fe/Ba	conv. [%]
1.3	0	0	4
1.3	0.16	0.12	25
1.3	0.48	0.37	44
1.3	0.8	0.62	60
1.3	1.12	0.86	82
1.3	1.6	1.23	86

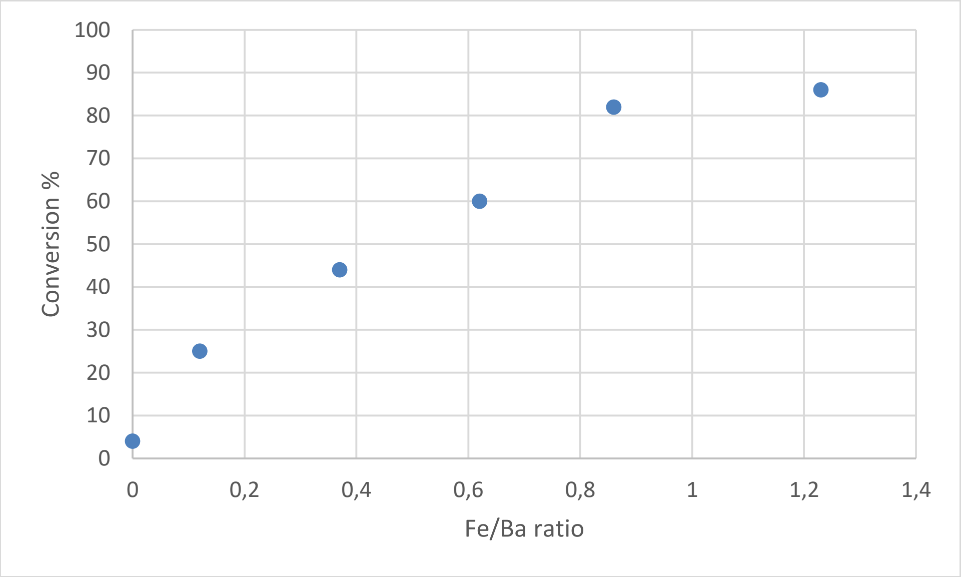

Figure S15. Conversion of benzene to cyclohexane (Table S8) as a function of the Fe/Ba ratio.

(7) Hydrogenation with AeFe catalysts (Ae = Mg, Ca, Sr, Ba): Benzene and cyclohexene hydrogenation with mixtures of Ae^0 and Fe^0 has been investigated. All Ae^0 metals show considerable activities when combined with Fe^0 (Table S9 and S10). It should be noted that Mg^0 alone in the form of a highly pyrophoric activated powder is fully inactive in benzene hydrogenation. In contrast, the MgFe combination shows a significant activity.

Table S9. Catalytic benzene-d_6 hydrogenation with Ae^0/Fe^0 catalysts (1 mol%, 150 °C, 50 bar H_2, 1.5 h). Conversion determined by ^{2}D NMR.

**catalyst**	**conv. [%]**	**TOF [h^{-1}]**
Ba/Fe	87	58
Sr/Fe	30	20
Ca/Fe	90	60
Mg/Fe	63	42

Table S10. Catalytic hydrogenation of cyclohexene to cyclohexane Ae^0/Fe^0 catalysts (2 mol%, 50 °C, 12 bar H_2, 1.0 h). Conversion determined by ^{1}H NMR.

**catalyst**	**conv. [%]**	**TOF [h^{-1}]**
Ba/Fe	>99	>50
Sr/Fe	>99	>50
Ca/Fe	>99	>50
Mg/Fe	38	19

5. Investigations towards the mechanism and the nature of the BaFe catalyst

The catalyst system that consists of Ba^0 and Fe^0 in a 1/1 ratio has been further investigated in order to draw conclusions on its nature. The following observations have been made:

(1) Grinding of the metals

Thorough mixing of Ba^0 and Fe^0 powders with a mortar and pestle increases the activity of the BaFe catalyst by at least a factor of 10 (Table S11).

Table S11. Impact of metal grinding on catalyst activation (1 mol% BaFe, 35 bar H_2).

Substrate	Treatment	T [°C]	t [h]	Conv. [%]
Benzene	unground	120	1	6
Benzene	ground	120	1	60
Toluene	unground	150	3	2
Toluene	ground	150	3	21

(2) Catalyst poisoning with metallic mercury

The activity of the BaFe catalyst is affected by addition of metallic mercury (Table S12). Although controversial,[12] inhibition of catalytic activity by Hg^0 suggests that a part of the catalytic system is heterogeneous.

Table S12. The effect of addition of metallic mercury on the activity of a BaFe catalyst in hydrogenation of C_6H_6 (750 μL, 1.5 mol% BaFe, 1.5 h, 150 °C, 50 bar H_2).

BaFe (mg)	BaFe (mmol)	Hg (mg)	Hg (mmol)	Conv. [%]
23	0.12	0	0	86
22	0.11	160	0.8	40
23	0.12	370	1.8	23

(3) Catalyst recycling

The BaFe catalyst has been recycled at least three times and could be reused without loss of activity (Table S13). Benzene was converted to cyclohexane using a BaFe catalyst. After separation of the spent catalyst with a magnet (Fig. S16), the catalyst was tested again for its activity. This procedure was repeated. The

catalyst remained active and did not show signs of activity loss. The mother liquor after a catalytic run is colorless and is not paramagnetic, excluding significant formation of organo-iron complexes. There is no residue after removal of all volatiles indicating that the catalyst after the catalytic run is insoluble. Also after using a larger quantity of BaFe catalyst (1.0 g) no visible quantities (naked eye or microscope) of catalyst remain after evaporation of all volatiles from the mother liquor.

Table S13. Catalytic benzene-d_6 hydrogenation with a recycled BaFe catalyst. Conversion determined by ^{2}D NMR.

catalyst	mol%	p(H_2) [bar]	T [°C]	t [h]	conv. [%]
BaFe cycle 1	3	50	150	0.5	94.3
BaFe cycle 2	3	50	150	0.5	93.9
BaFe cycle 3	3	50	150	0.5	93.0

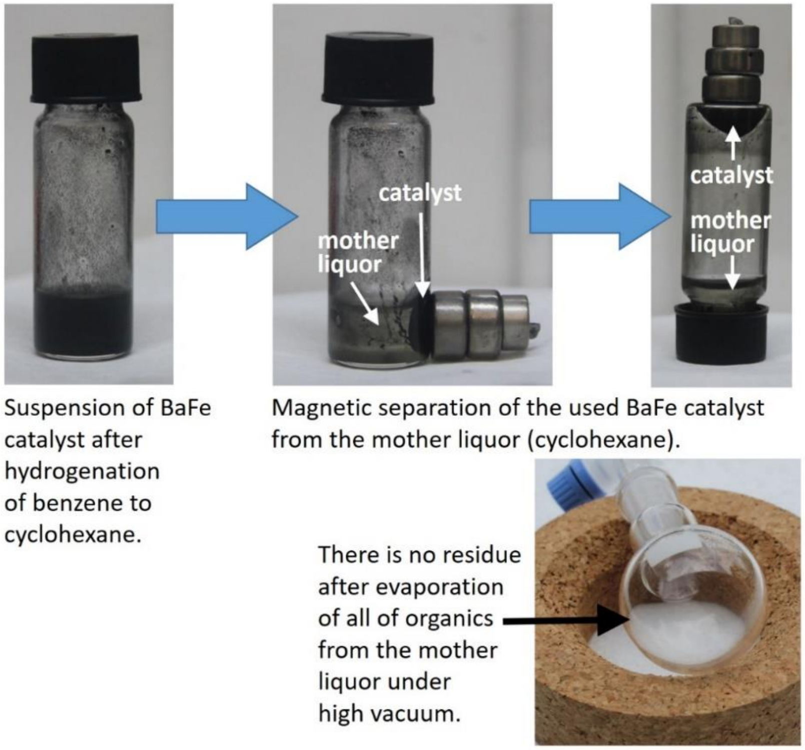

Figure S16. Facile recycling of the spent BaFe catalyst by magnetic separation. The mother liquor does not contain metal salts.

(4) Investigations on a spent BaFe catalyst

A spent BaFe catalyst (from: benzene-to-cyclohexane conversion, 5 mol% catalyst, 150 °C, 50 bar H_2) was separated from the mother liquor with a magnet, dried under high vacuum, and analyzed. Elemental analysis (Fa. Kolbe) gave the following results (w%): Ba 70.12, Fe 27.97, C 1.41, H 0.17, N 0.00. This equals the following molar ratio (mol%): Ba 39.35, Fe 38.58, C 9.03, H 13.04, N 0.00. This fits for a heterogeneous catalyst with a Ba/Fe ratio of circa 1/1 and some CH-organics adsorbed on the metal surface. Based on the C/H ratio of circa 1/1.45 this could be a mixture of C_6H_6 and C_6H_{12} (or cyclohexadiene and cyclohexene intermediates).

Proof for the presence of reducing Ba^0 metal – Reaction with benzophenone: Addition of benzophenone to a spent BaFe catalyst suspended in a toluene/THF mixture led to immediate formation of a dark-blue color, indicative for formation of the radical anion $Ph_2CO^{\bullet-}$ (Fig. S17).

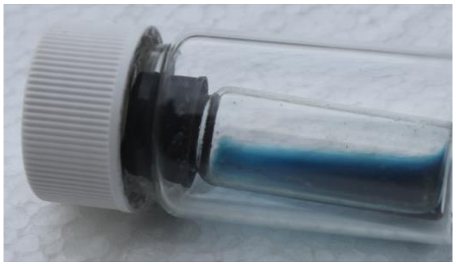

Figure S17. Formation of the $Ph_2CO^{\bullet-}$ radical anion by addition of Ph_2CO to a spent BaFe catalyst (10 mg) suspended in a toluene/THF mixture.

Proof for the presence of reducing Ba^0 metal – Reaction with pivaldehyde: 30 mg (0.15 mmol) of a used BaFe catalyst (C_6D_6, 150 °C, 50 bar H_2, 6 h) was suspended in C_6D_6 (750 µL) and 12 µL of pivaldehyde (0.11 mmol) was added. The suspension was allowed to stir at room temperature for 3 h. The mixture was then filtered and analyzed by ^{1}H NMR. The only reaction product was determined to be 2,2,5,5-tetramethyl-3-hexanone which was formed by the reductive coupling of two equivalents of pivaldehyde in the presence of Ba^0 to give barium 2,2,5,5-tetramethylhexane-3,4-diolate and subsequent elimination of BaO (Fig. S18).

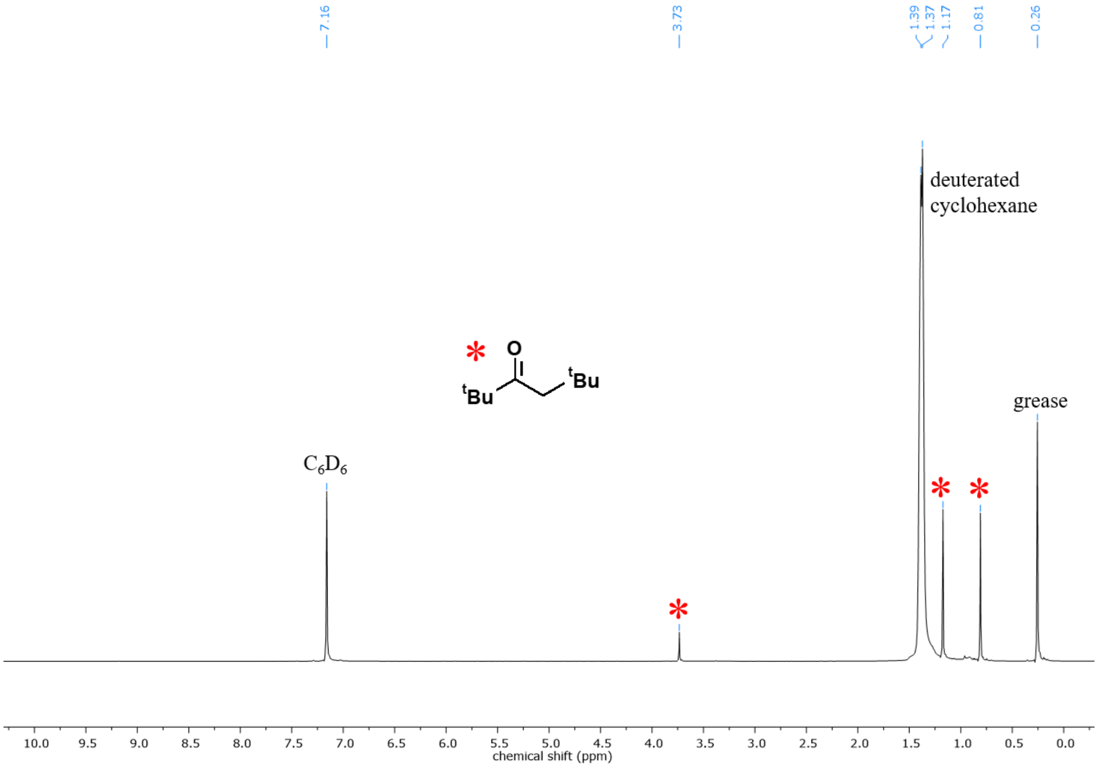

Figure S18. Formation of be 2,2,5,5-tetramethyl-3-hexanone formed by reaction of a spent BaFe catalyst with pivaldehyde (proof for Ba^0). Deuterated cyclohexane was formed by hydrogenation of C_6D_6.

Proof for the presence of Ba hydride species – Reaction with CD_3OD: A septum-sealed reaction vial with a suspension of 100 mg of the used BaFe catalyst in toluene (2 mL) under N_2 was equipped with a magnetic stir bar. Methanol-d_4 was added with syringe to the stirred suspension. The evolving gas was passed via Teflon tubing in a screw-capped NMR tube with septum and bubbled through cooled $CDCl_3$. When the gas evolution had stopped, the NMR tube was sealed with a closed screw cap and a ^{1}H NMR spectrum was recorded (Fig. S19). Formation of H_2 and H-D can only be explained by the presence of Ba-H species. Ba^0 reacts with CD_3OD to give $Ba(OCD_3)_2$ and D_2.

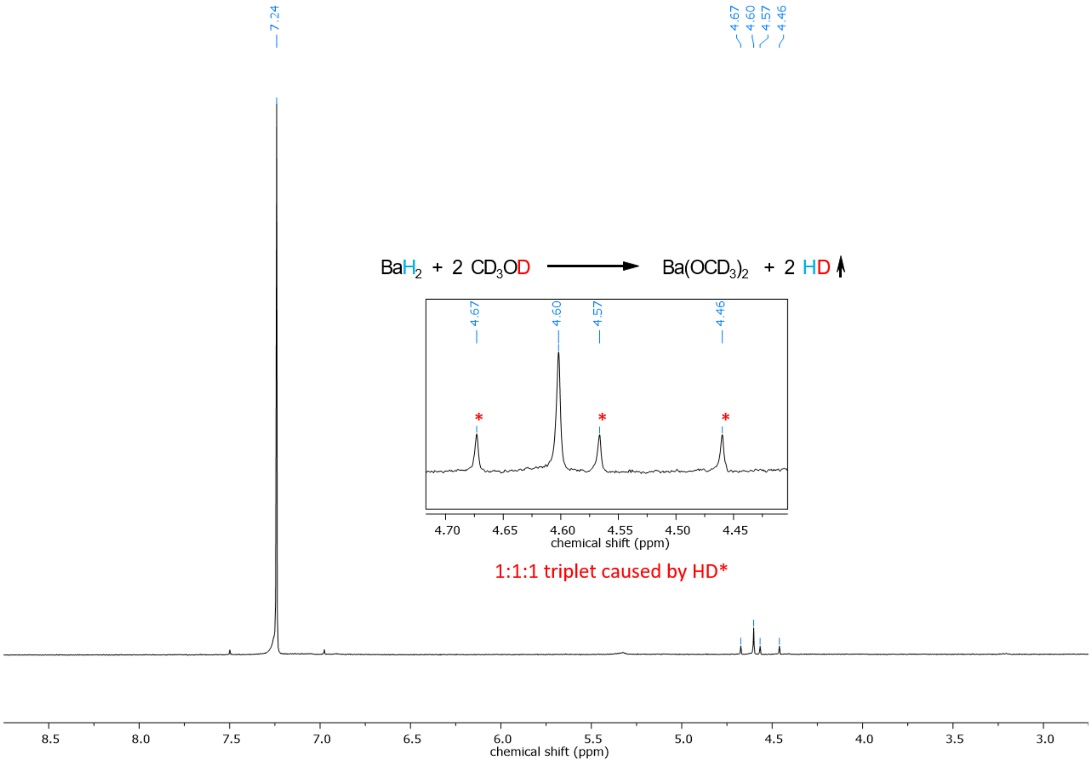

Figure S19. ^{1}H NMR spectrum of the gas that has been formed from a used BaFe catalyst which was quenched with methanol-d_4 (H_2 singlet 4.60 ppm, HD triplet at 4.57 ppm).

Proof for the presence of reducing Ba hydride species – Reaction with Me_3SiCl: 27 mg (0.14 mmol) of a used BaFe catalyst (C_6D_6, 150 °C, 50 bar H_2, 6 h) was suspended in C_6D_6 (750 µL) and 50 µL of Me_3SiCl (0.39 mmol, excess) was added. The suspension was allowed to stir in a glovebox at room temperature for 3 days. The mixture was then filtered and analyzed by ^{1}H NMR (Fig. S20). Although the expected product Me_3SiH has a boiling point of 6 °C, it could be clearly detected in the mother liquor (Si-H signal at 3.99 ppm, multiplet). Also a sharp singlet at 3.20 ppm was observed which could be assigned to the distribution product SiH_4.[13]

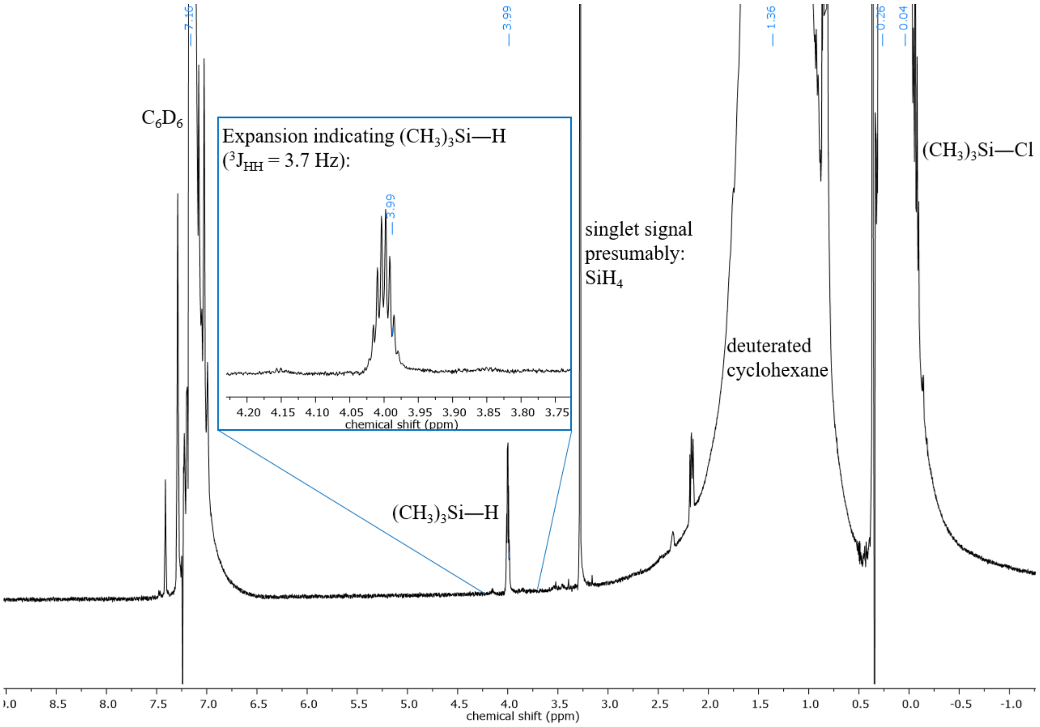

Figure S20. ^{1}H NMR spectrum of the gaseous product that has been formed from a used BaFe catalyst (used in the C_6D_6 to cyclohexane-d_6 reduction) which was quenched with Me_3SiCl.

6. Monitoring of hydrogenation catalysis by ^{1}H NMR

The conversion in catalytic hydrogenation experiments with the BaFe catalyst was determined by ^{1}H NMR and GC-MS measurements. These spectra have been ordered according to substrate in the same order as in Fig. 3 in the manuscript. Also reference spectra for the substrates and for catalytic runs with only Fe^0 or only Ba^0 (additional data to those reported in ref. [9]) are incorporated.

1-Hexene

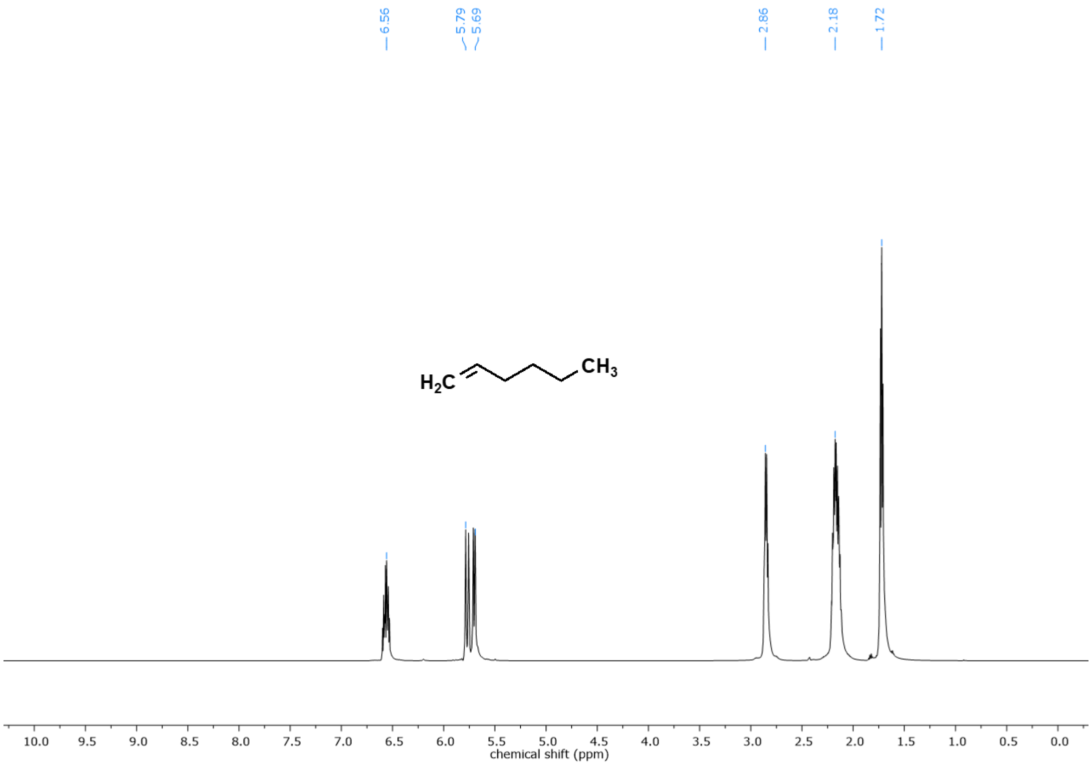

Figure S21. ^{1}H NMR reference spectrum (600 MHz, no-D NMR neat, 298 K) of 1-hexene.

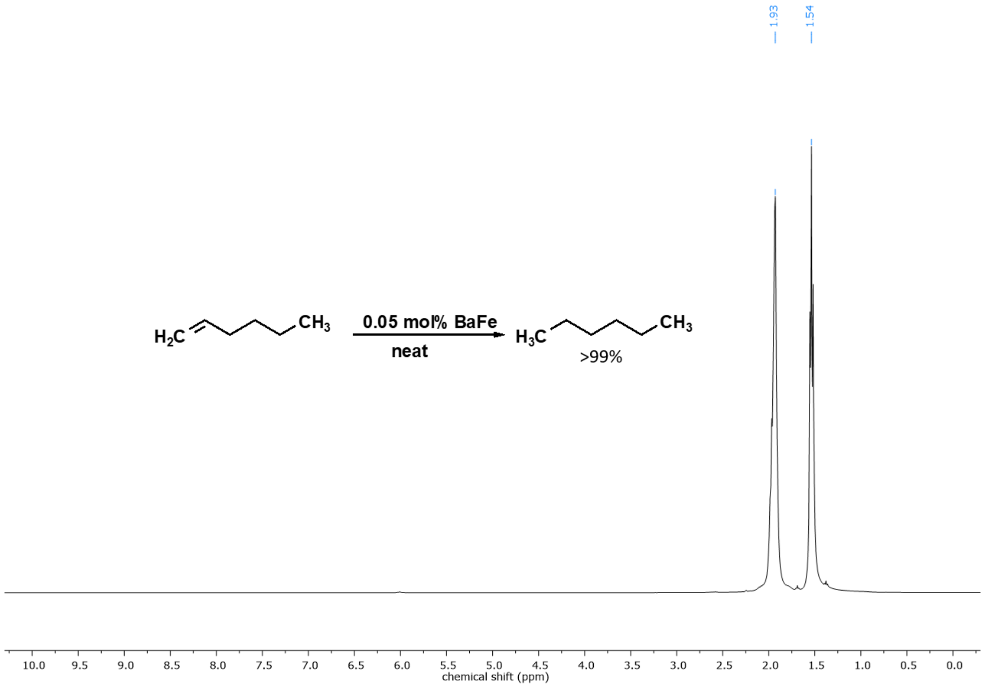

Figure S22. ^{1}H NMR spectrum (600 MHz, no-D NMR neat, 298 K) after catalytic hydrogenation of 1-hexene (neat, 20 bar H_2) with 0.05 mol% BaFe at 100°C for 15 min. Conversion to hexane: >99%.

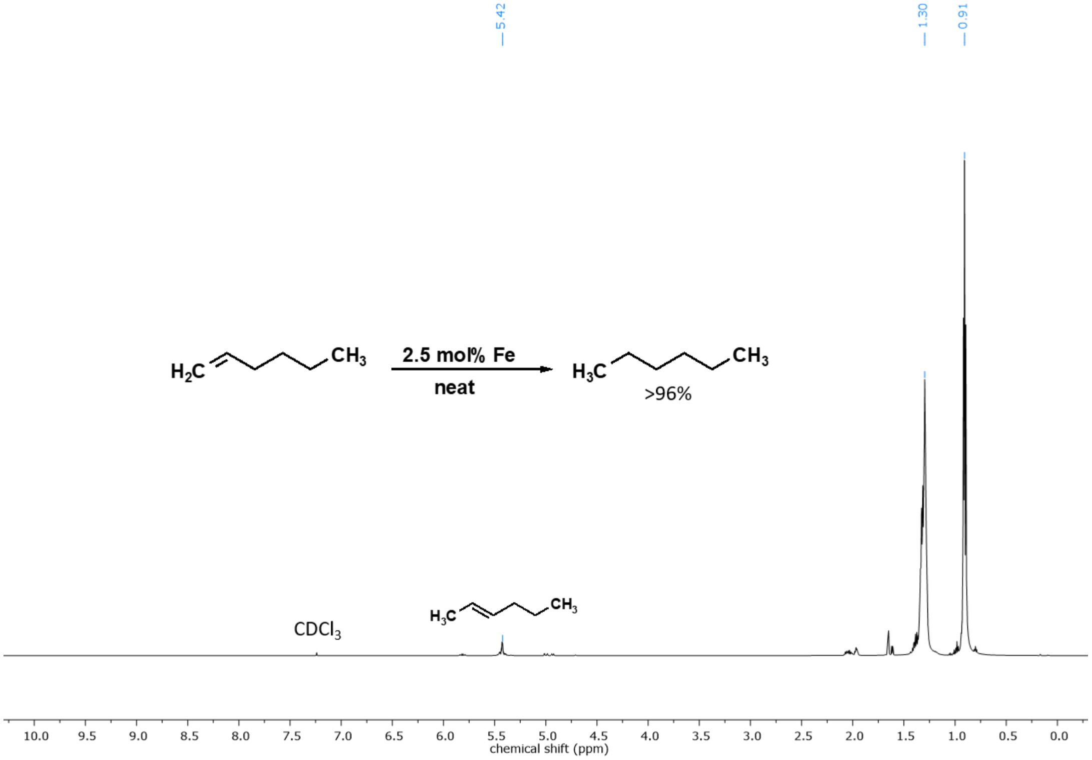

Figure S23. ^{1}H NMR spectrum (600 MHz, $CDCl_3$, 298 K) after catalytic hydrogenation of 1-hexene (neat, 20 bar H_2) with 2.5 mol% Fe at 150°C for 10 minutes. Conversion to hexane >96%; formation of 2-hexene detectable (< 4%).

Cyclohexene

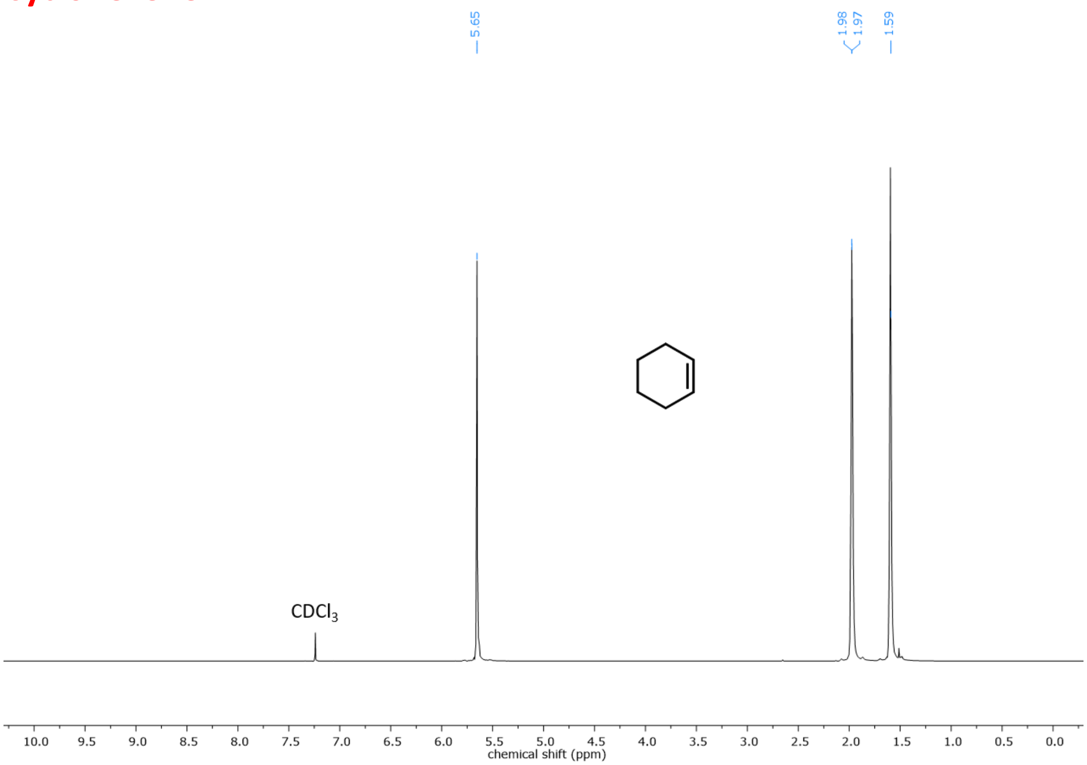

Figure S24. ^{1}H NMR reference spectrum (600 MHz, $CDCl_3$, 298 K) of cyclohexene.

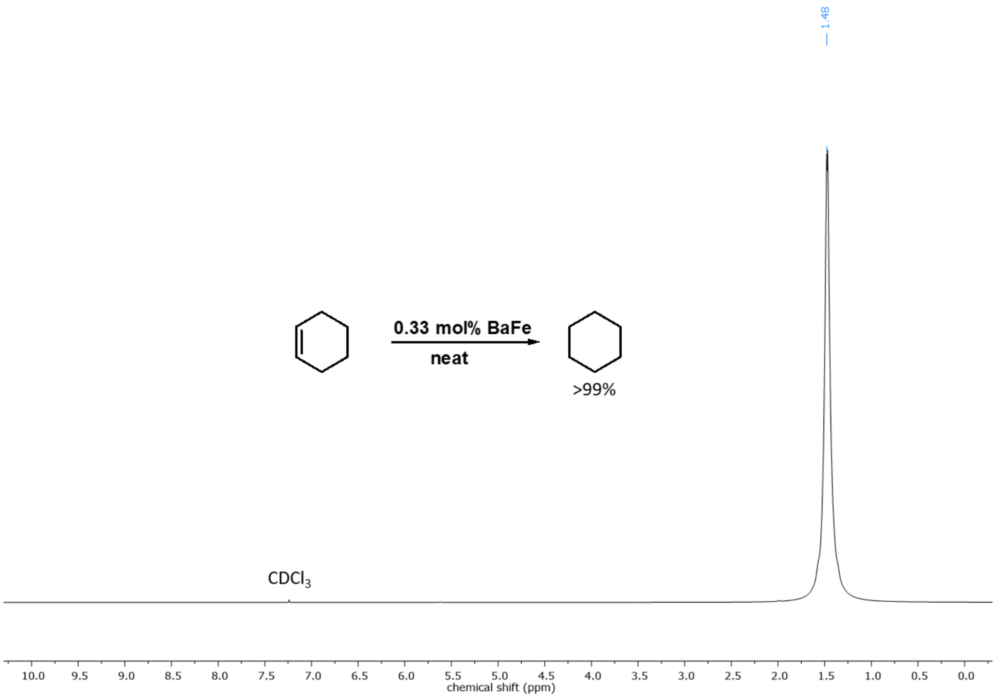

Figure S25. ^{1}H NMR spectrum (600 MHz, $CDCl_3$, 298 K) after catalytic hydrogenation of cyclohexene (neat, 12 bar H_2) with 0.33 mol% BaFe at 100°C for 15 min. Conversion to cyclohexane: >99%.

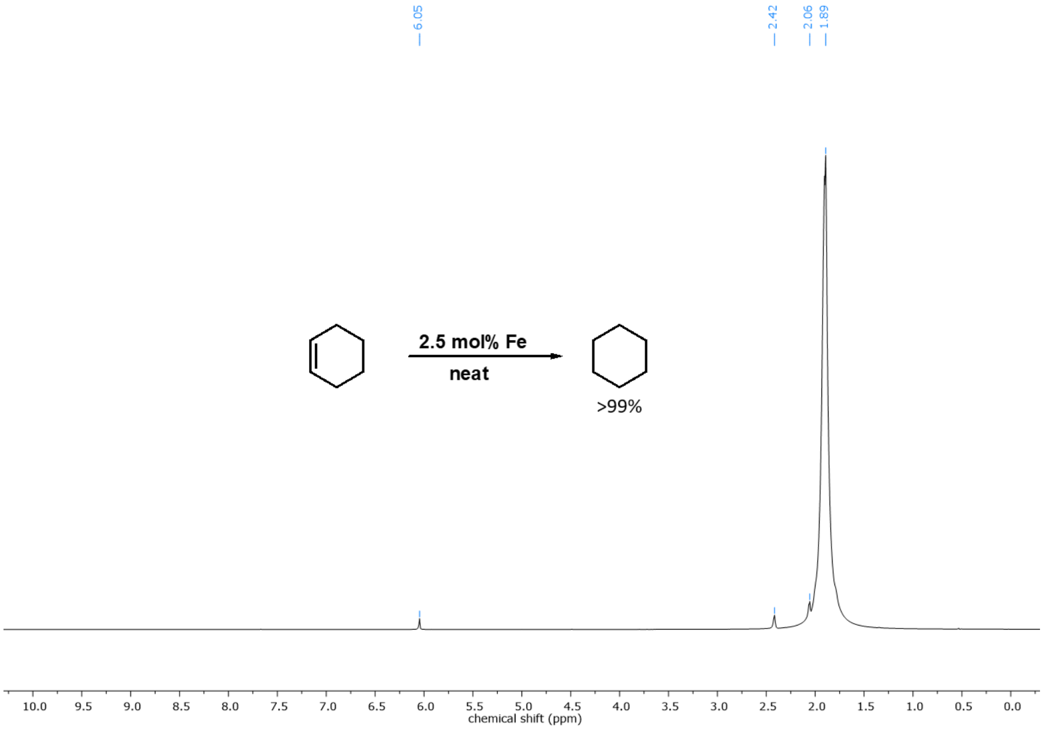

Figure S26. ^{1}H NMR spectrum (600 MHz, no-D NMR neat, 298 K) after catalytic hydrogenation of cyclohexene (neat, 50 bar H_2) with 2.5 mol% Fe at 150°C for 4 h. Conversion to cyclohexane >99%.

cis-3-Hexene

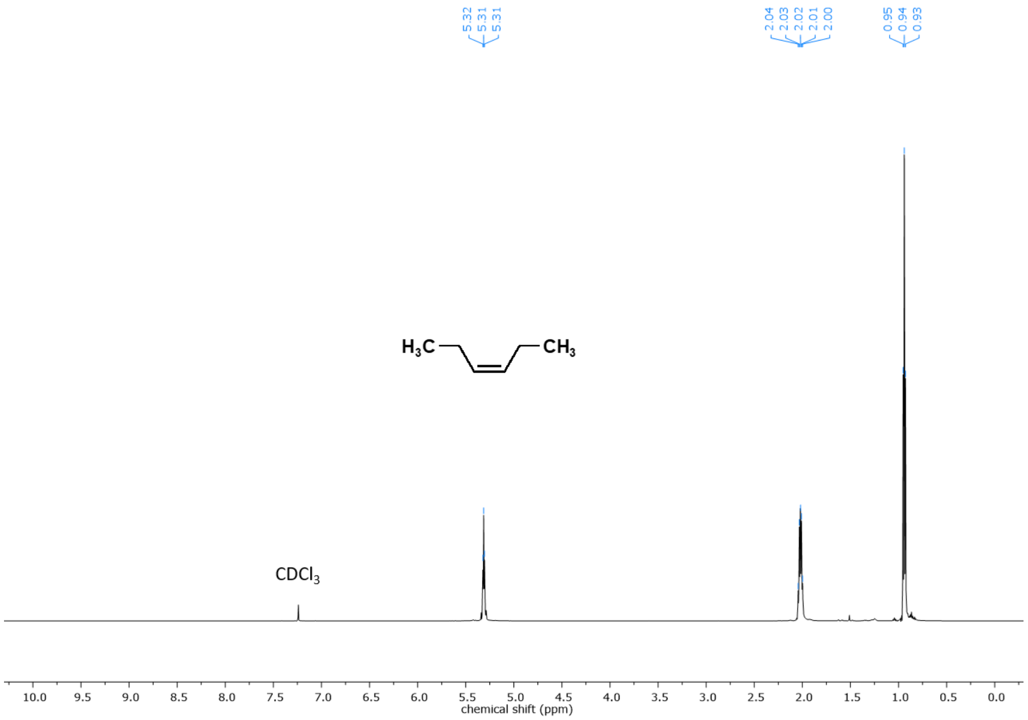

Figure S27. ^{1}H NMR reference spectrum (600 MHz, $CDCl_3$, 298 K) of *cis*-3-hexene.

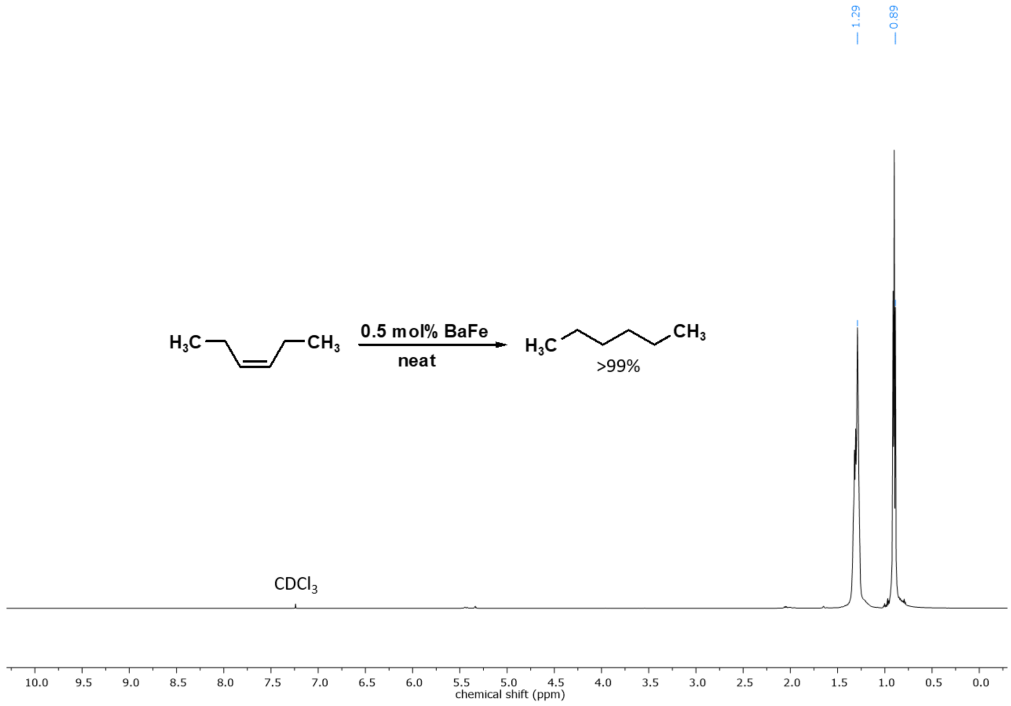

Figure S28. ^{1}H NMR spectrum (600 MHz, $CDCl_3$, 298 K) after catalytic hydrogenation of *cis*-3-hexene (neat, 12 bar H_2) with 0.5 mol% BaFe at 120°C for 15 min. Conversion to hexane: >99%.

trans-3-Hexene

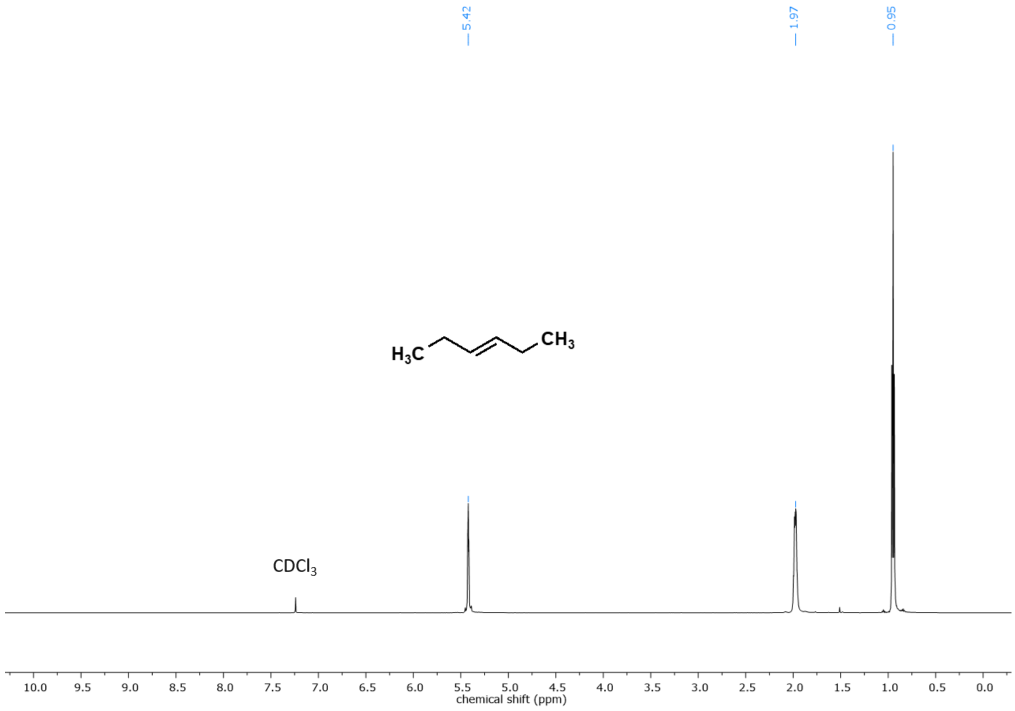

Figure S29. ^{1}H NMR reference spectrum (600 MHz, $CDCl_3$, 298 K) of *trans*-3-hexene.

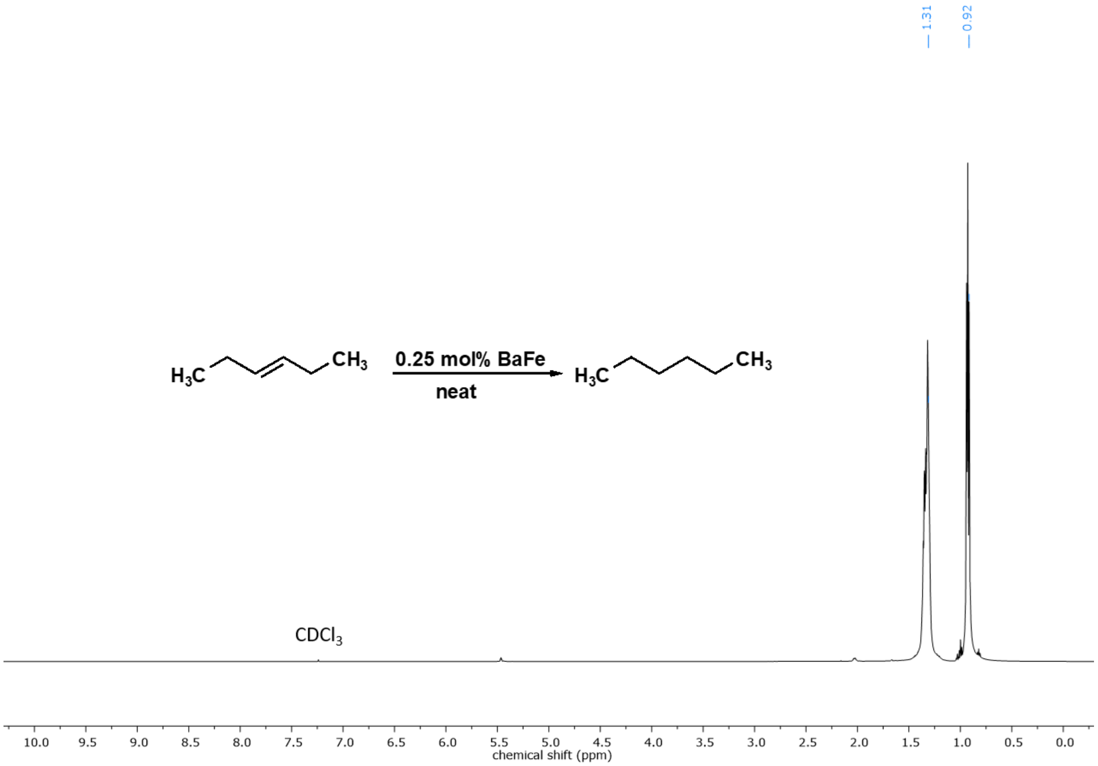

Figure S30. ^{1}H NMR spectrum (600 MHz, $CDCl_3$, 298 K) after catalytic hydrogenation of *trans*-3-hexene (neat, 12 bar H_2) with 0.25 mol% BaFe at 100°C for 30 min. Conversion to hexane: >99%.

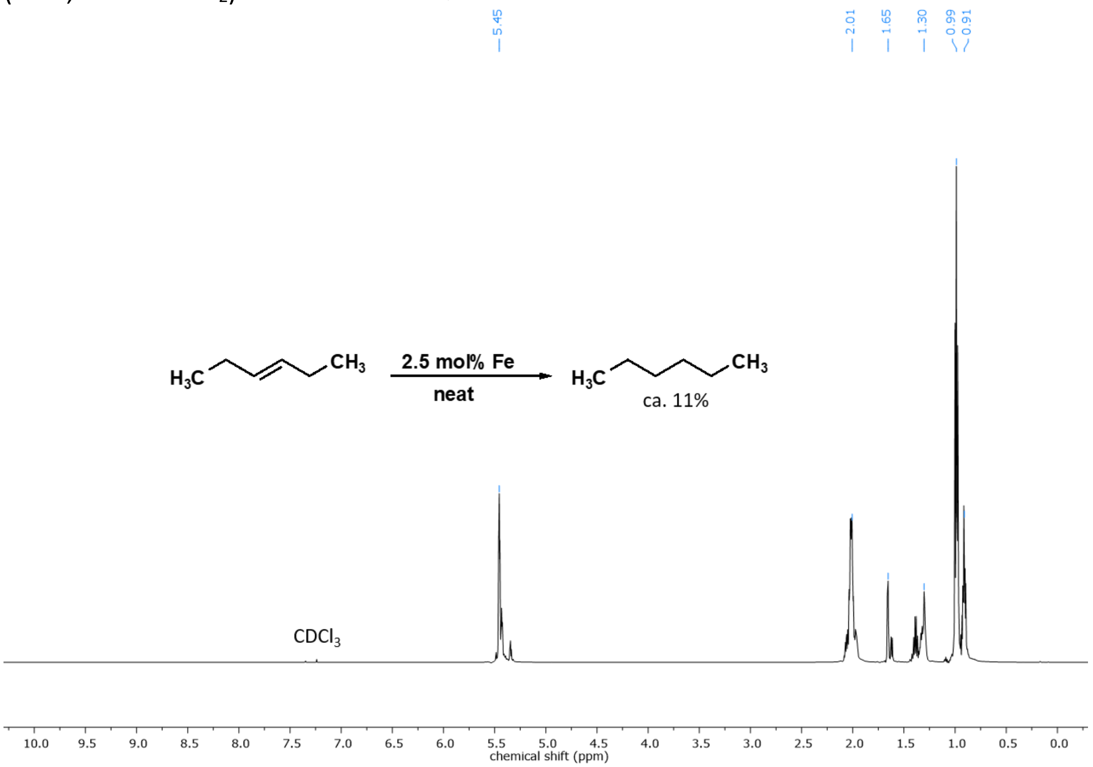

Figure S31. ^{1}H NMR spectrum (600 MHz, $CDCl_3$, 298 K) after catalytic hydrogenation of *trans*-3-hexene (neat, 20 bar H_2) with 2.5 mol% Fe at 150°C for 5 h. Conversion (GC/MS) to hexane: ca. 11%.

1-Methylcyclohexene

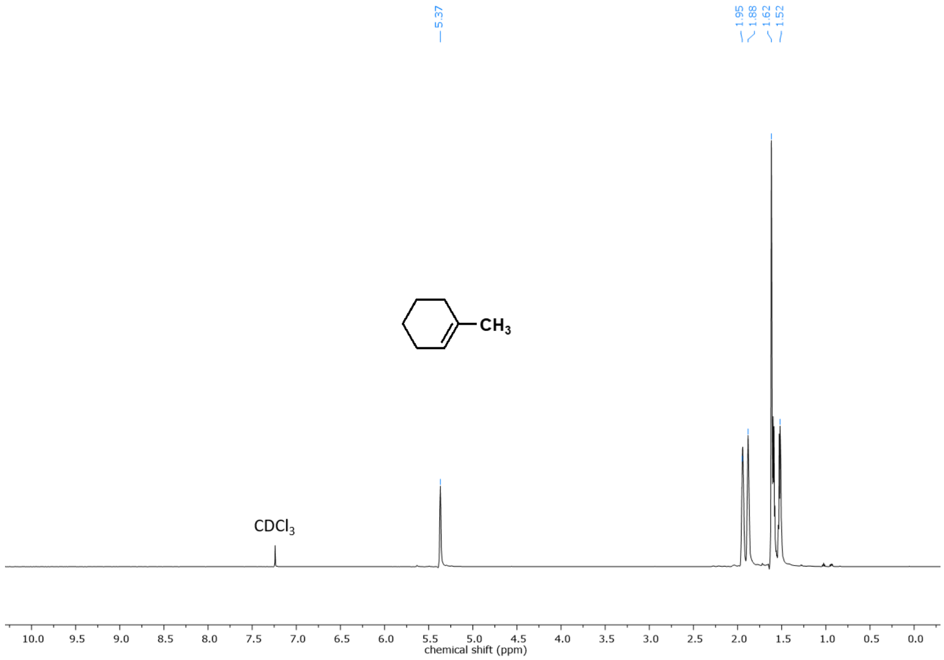

Figure S32. ^{1}H NMR reference spectrum (600 MHz, $CDCl_3$, 298 K) of 1-methylcyclohexene.

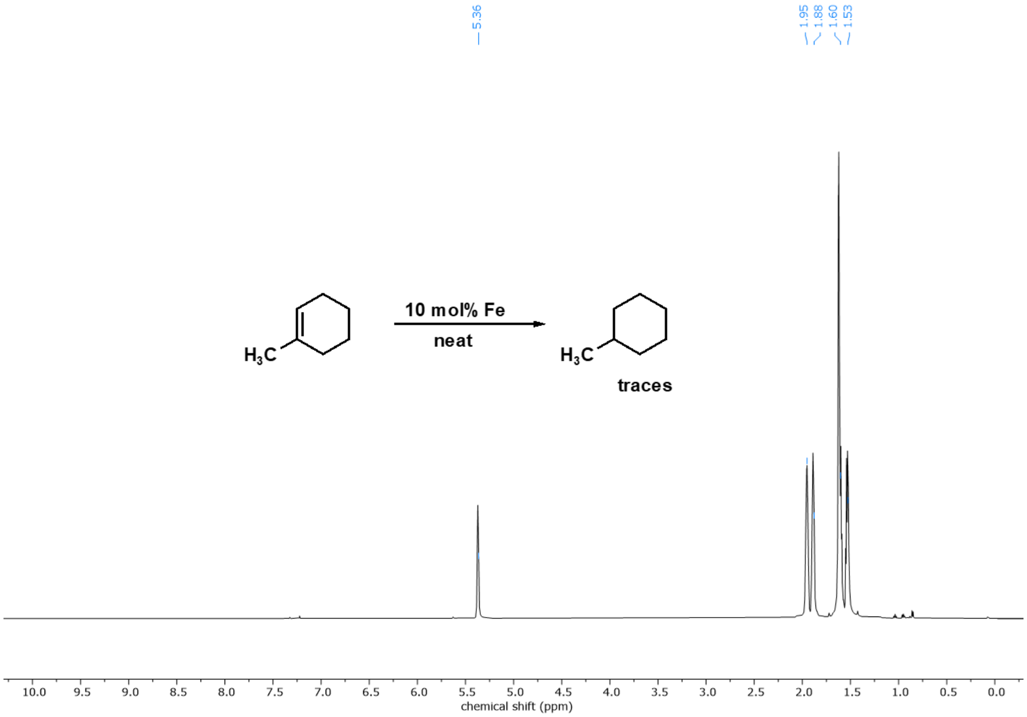

Figure S33. ^{1}H NMR spectrum (600 MHz, $CDCl_3$, 298 K) after attempted catalytic hydrogenation of 1-methylcyclohexene (neat, 20 bar H_2) with 10 mol% Fe at 150°C for 24 h. Conversion to methylcyclohexane: <1%.

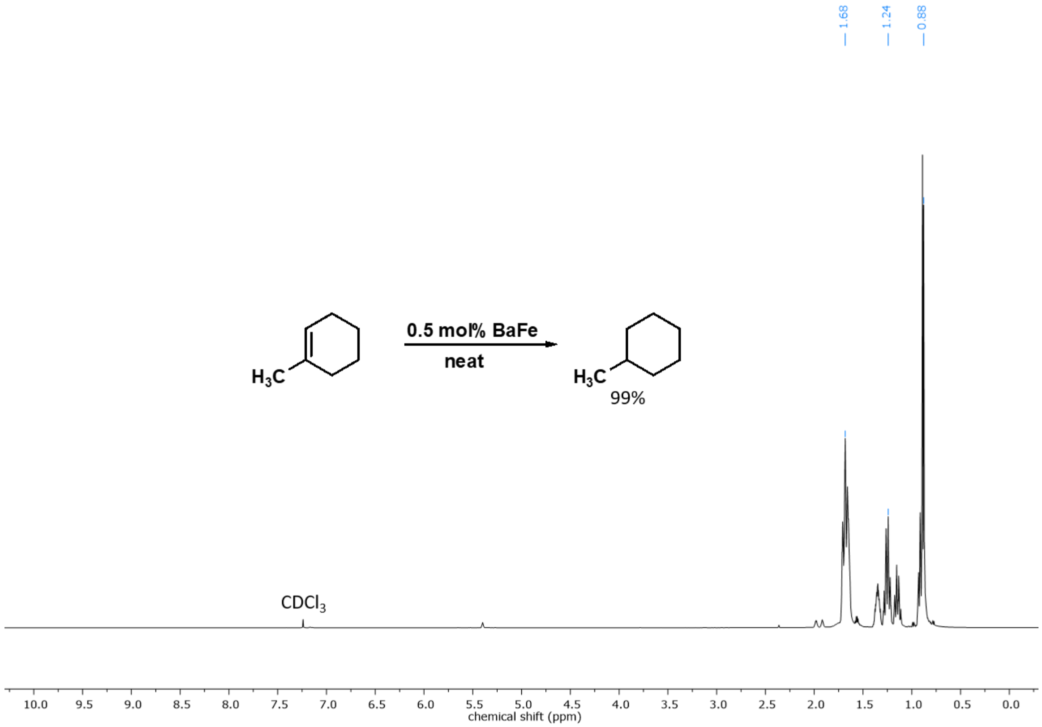

Figure S34. ^{1}H NMR spectrum (600 MHz, $CDCl_3$, 298 K) after catalytic hydrogenation of 1-methylcyclohexene (neat, 20 bar H_2) with 0.5 mol% BaFe at 150°C for 30 min. Conversion to methylcyclohexane: 99%.

1,1-Diphenylethylene

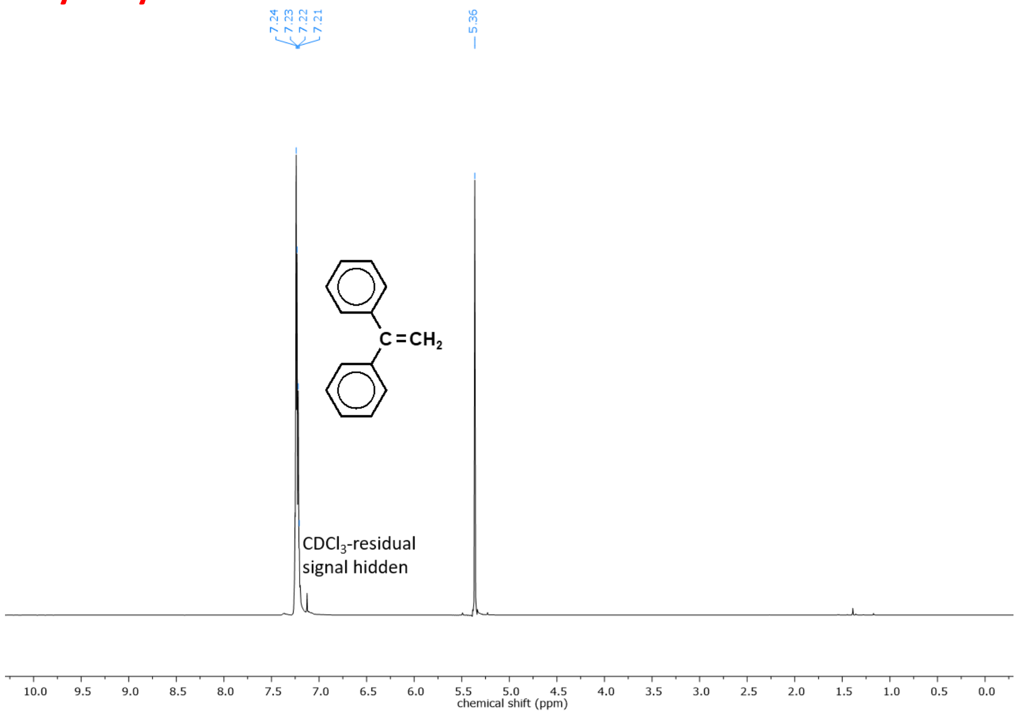

Figure S35. ^{1}H NMR reference spectrum (600 MHz, $CDCl_3$, 298 K) of 1,1-diphenylethylene.

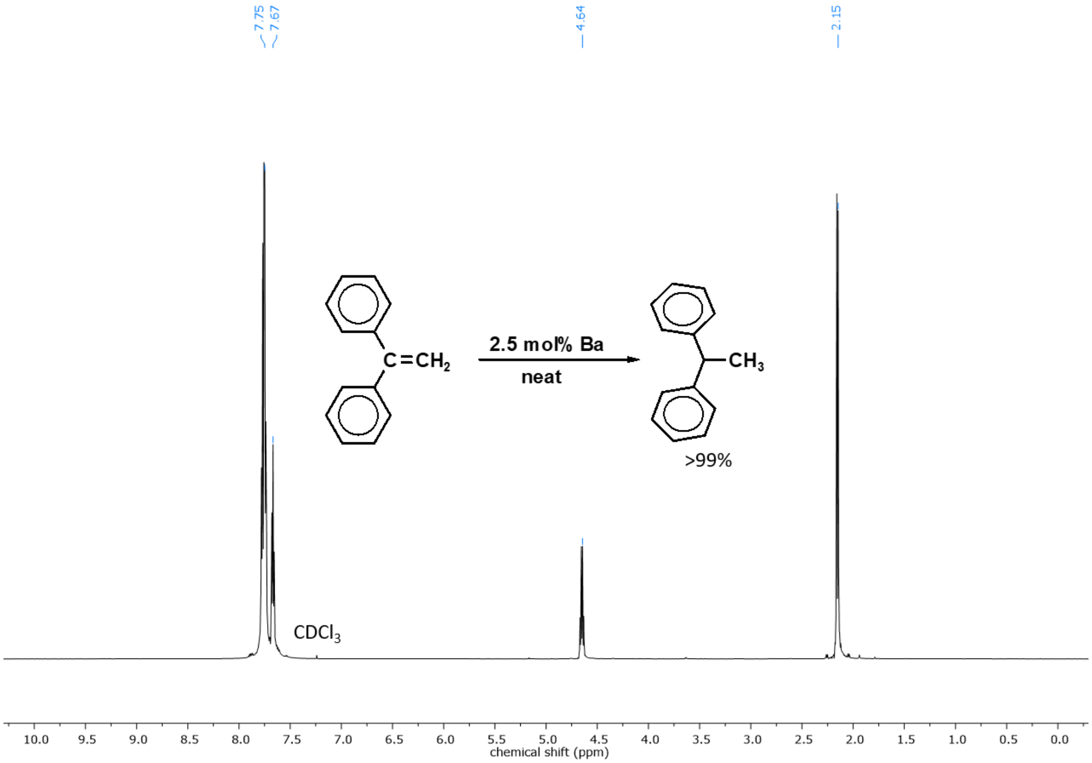

Figure S36. ^{1}H NMR spectrum (600 MHz, $CDCl_3$, 298 K) after catalytic hydrogenation of 1,1-diphenylethylene (neat, 20 bar H_2) with 2.5 mol% Ba at 120°C for 30 min. Conversion to 1,1-diphenylethane: >99%.

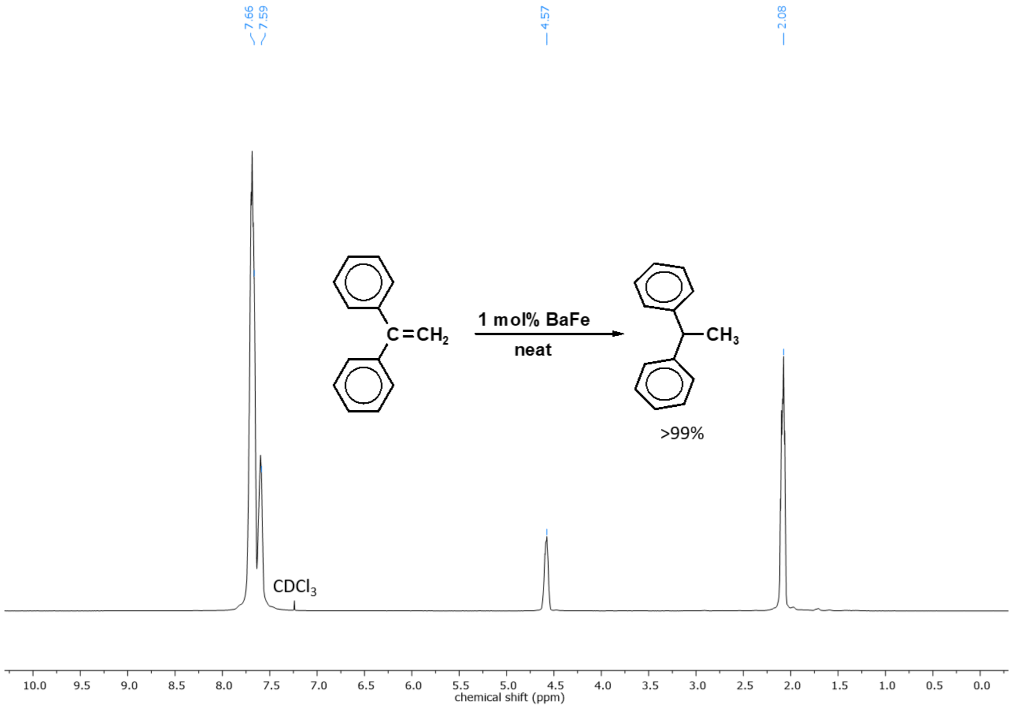

Figure S37. ^{1}H NMR spectrum (600 MHz, $CDCl_3$, 298 K) after catalytic hydrogenation of 1,1-diphenylethylene (neat, 20 bar H_2) with 1 mol% BaFe at 120°C for 30 min. Conversion to 1,1-diphenylethane: >99%.

Triphenylethylene

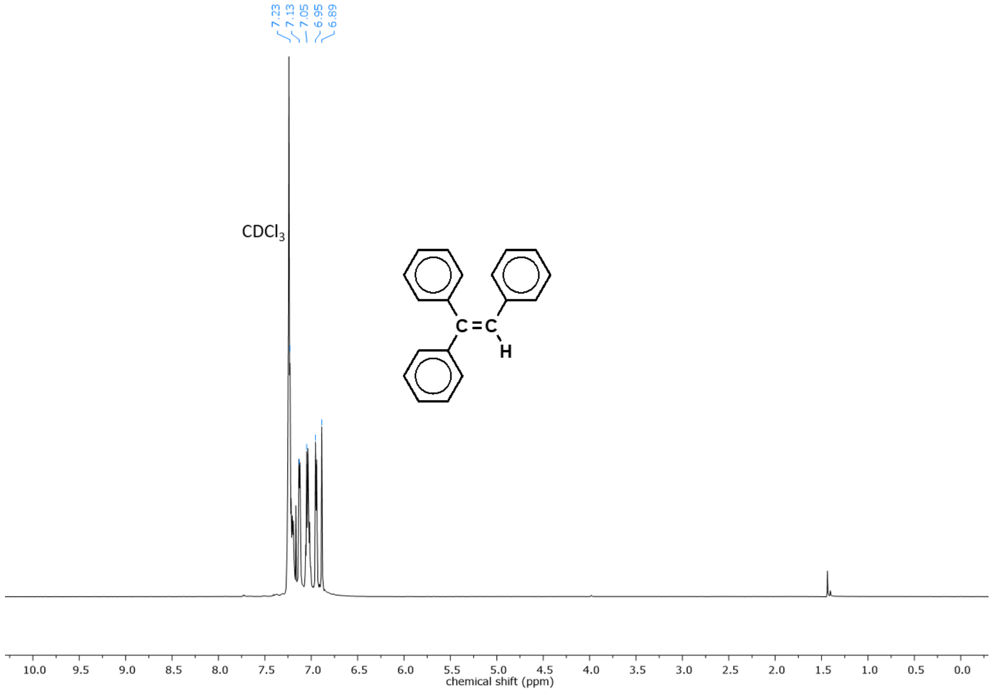

Figure S38. ^{1}H NMR reference spectrum (600 MHz, $CDCl_3$, 298 K) of 1,1,2-triphenylethylene.

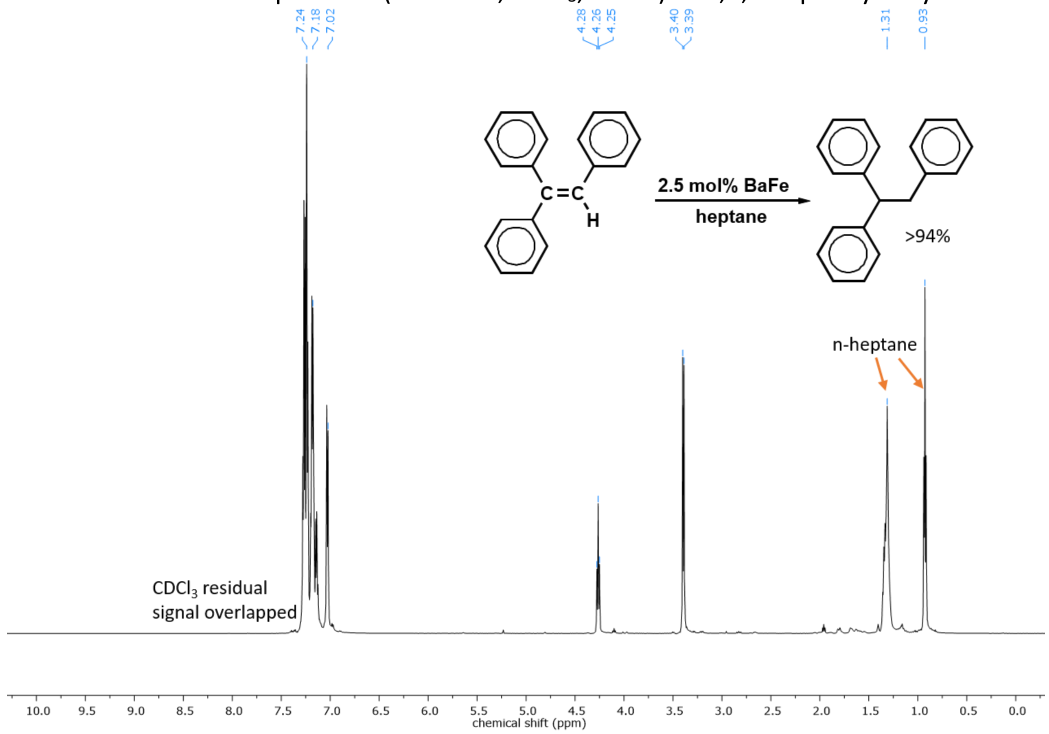

Figure S39. ^{1}H NMR spectrum (600 MHz, $CDCl_3$, 298 K) after catalytic hydrogenation of 1,1,2-triphenylethylene (100 mg, 0.39 mmol in 750 µL n-heptane, 20 bar H_2) with 2.5 mol% BaFe at 120°C for 30 min. Conversion to 1,1,2-triphenylethane was determined by NMR and GC/MS analysis: >94%.

Tetraphenylethylene

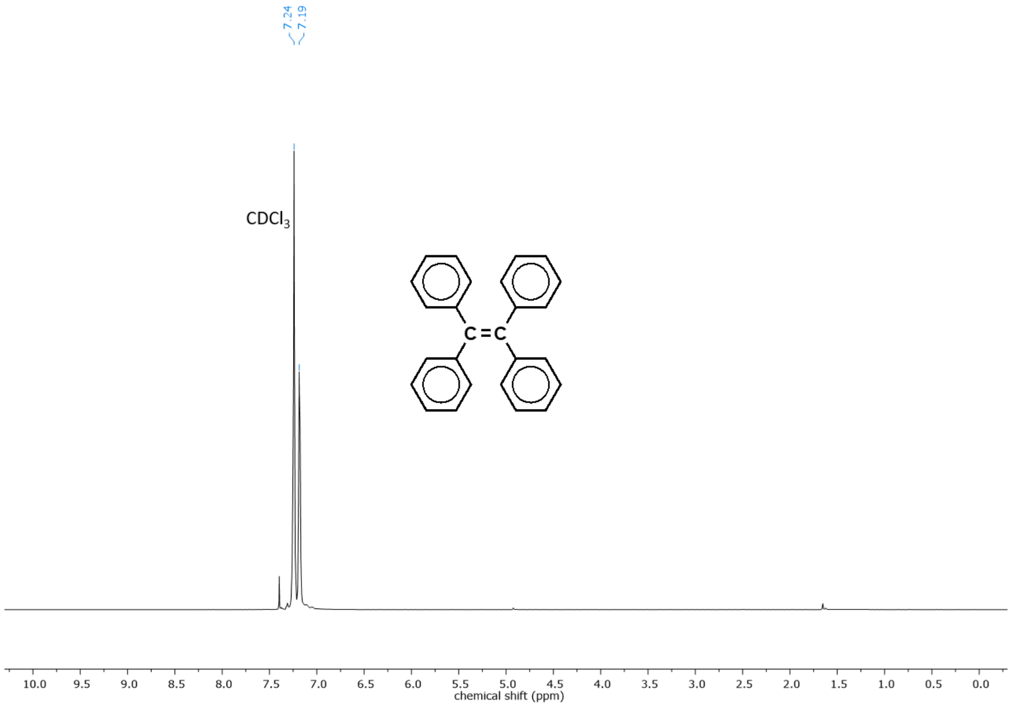

Figure S40. ^{1}H NMR reference spectrum (600 MHz, $CDCl_3$, 298 K) of tetraphenylethylene.

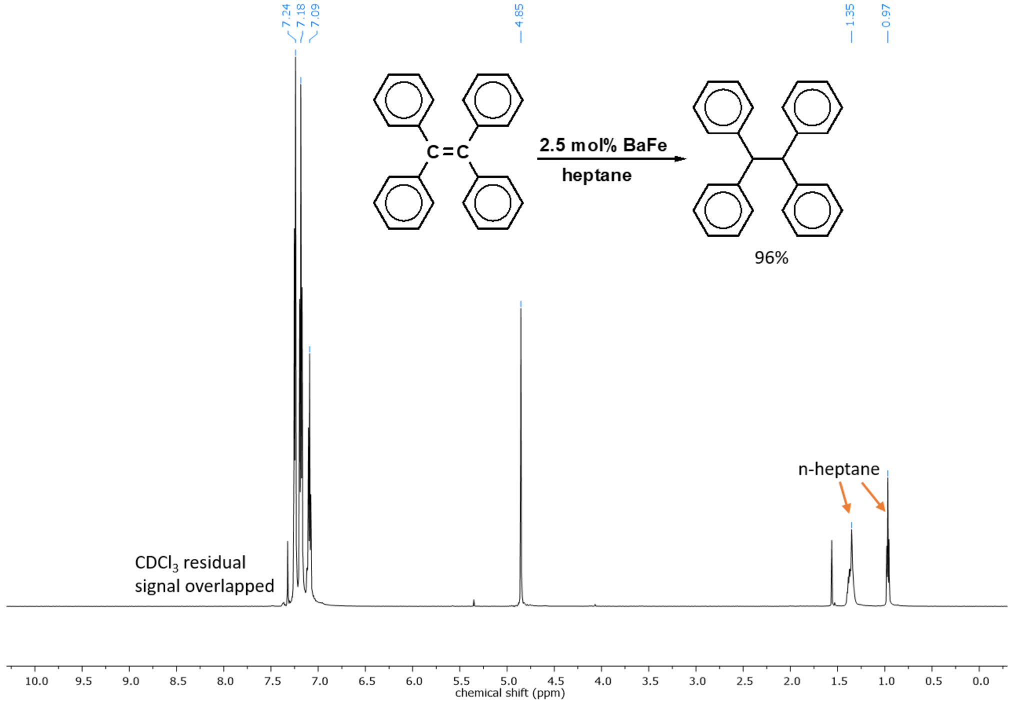

Figure S41. ^{1}H NMR spectrum (600 MHz, $CDCl_3$, 298 K) after catalytic hydrogenation of tetraphenylethylene (100 mg, 0.3 mmol in 750 µL *n*-heptane, 20 bar H_2) with 2.5 mol% BaFe at 120°C for 30 min. Conversion to tetraphenylethane was confirmed by ^{1}H NNMR and GC/MS analysis: 96%.

Benzene

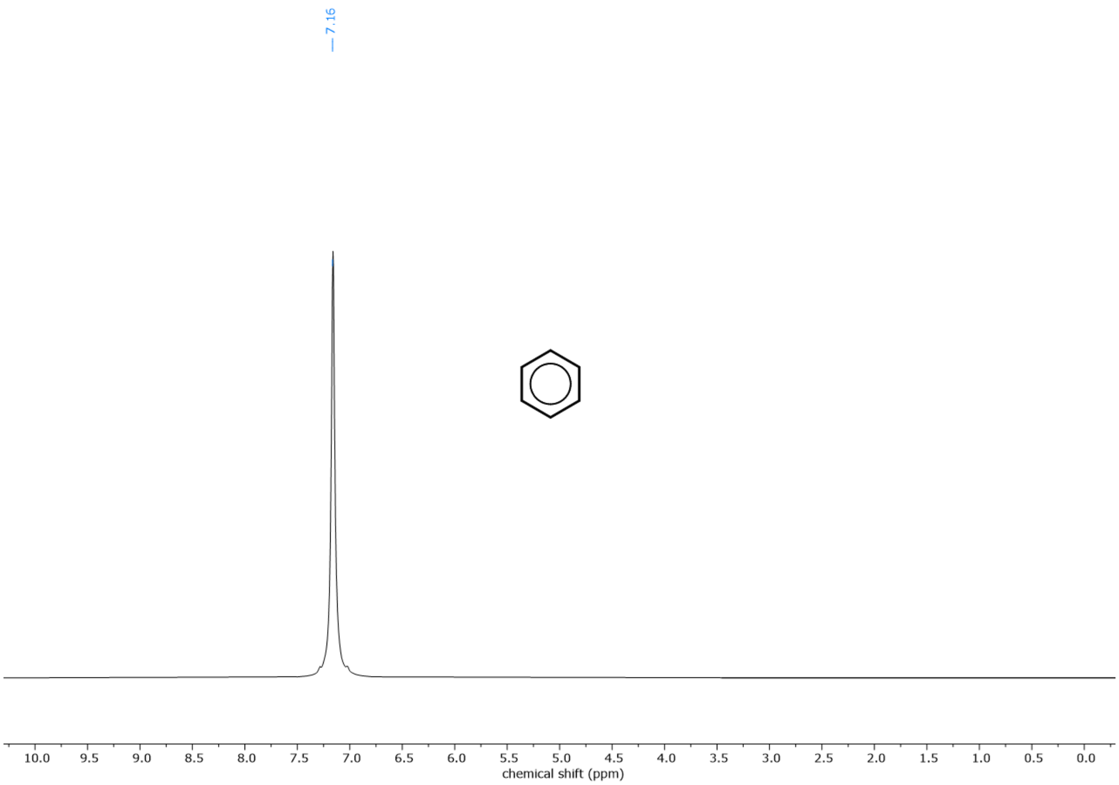

Figure S42. ^{1}H NMR reference spectrum (600 MHz, no-D NMR neat, 298 K) of benzene.

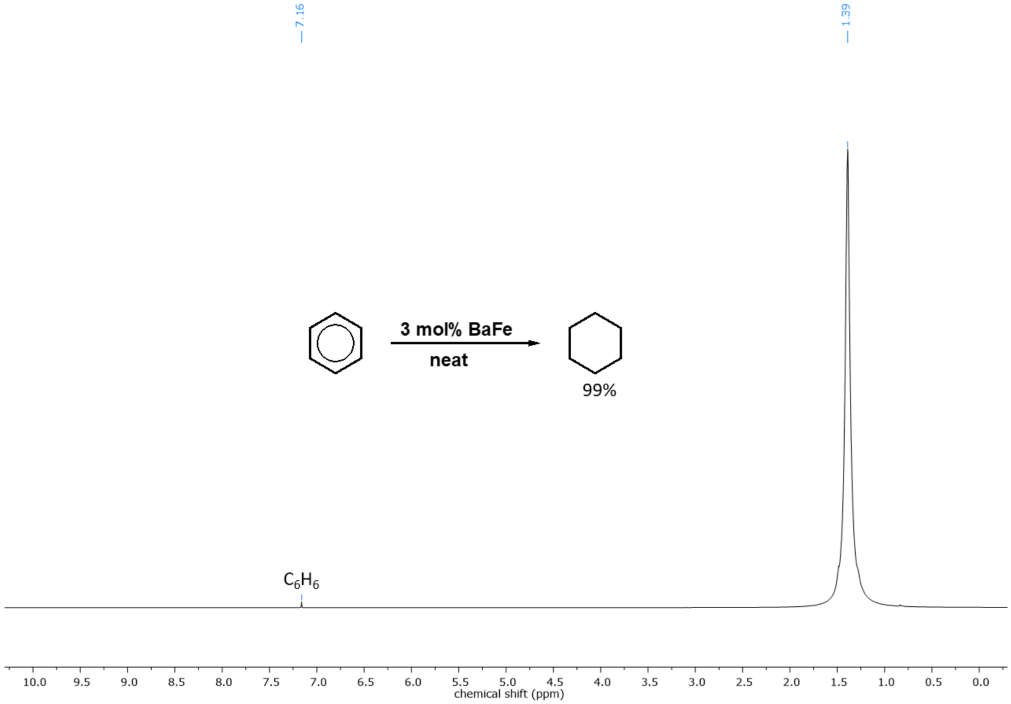

Figure S43. ^{1}H NMR spectrum (600 MHz, no-D NMR neat, 298 K) after catalytic hydrogenation of benzene (neat, 50 bar H_2) with 3 mol% BaFe at 150°C for 30 min. Conversion to cyclohexane: 99%.

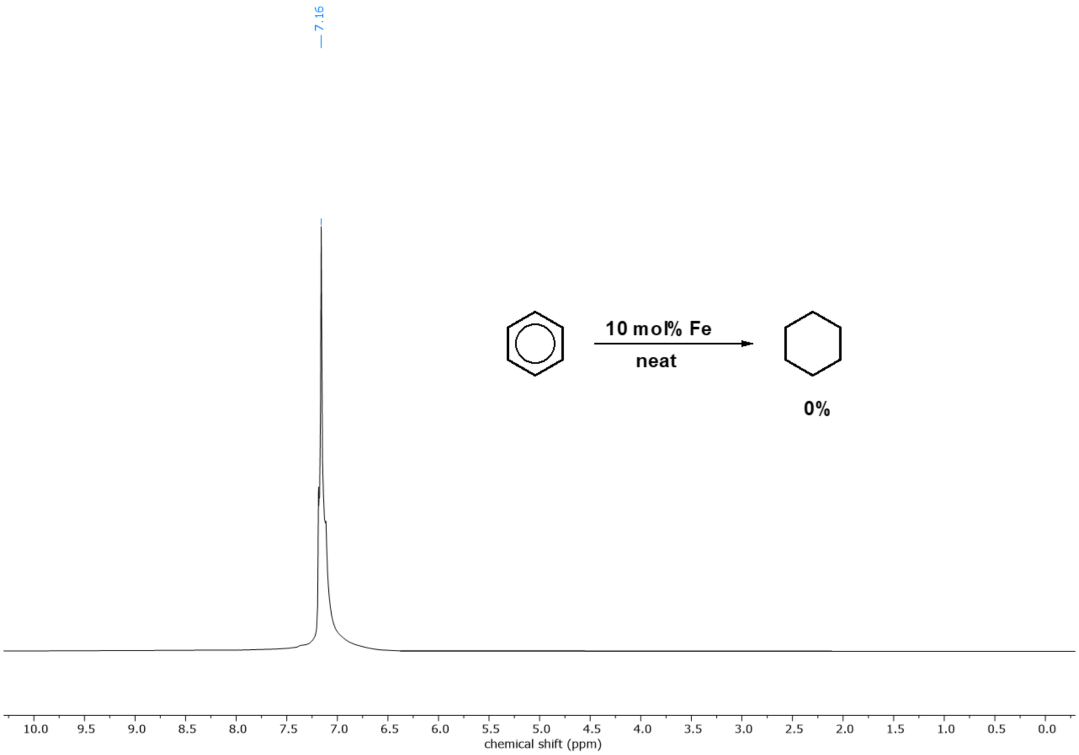

Figure S44. ^{1}H NMR spectrum (600 MHz, no-D NMR neat, 298 K) after catalytic hydrogenation of benzene (neat, 50 bar H_2) with 10 mol% Fe at 150°C for 24 h. Conversion to cyclohexane was not observed.

Toluene

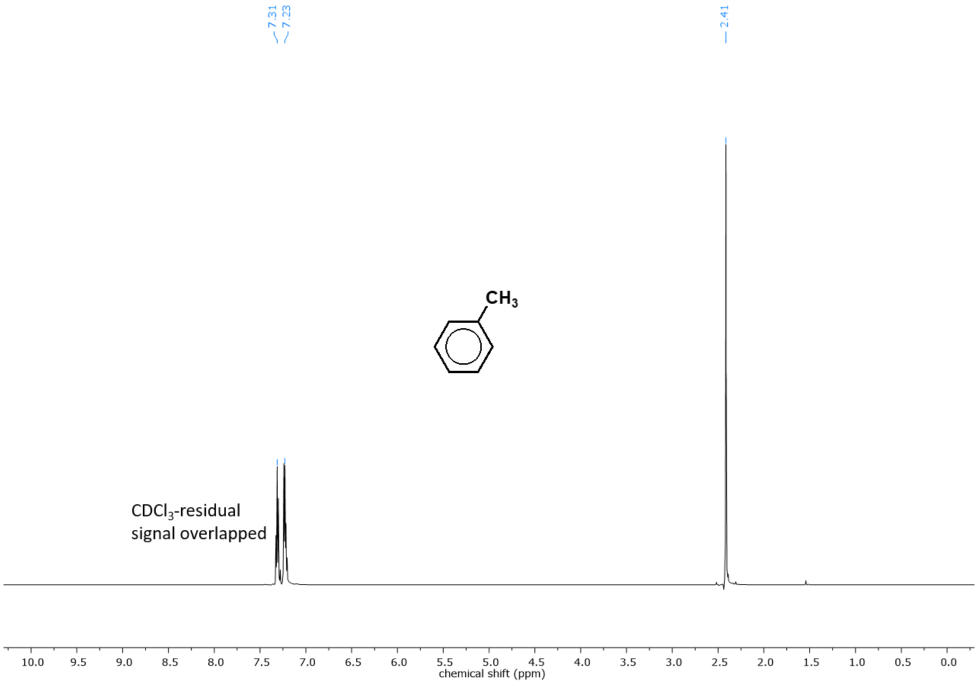

Figure S45. ^{1}H NMR reference spectrum (600 MHz, $CDCl_3$, 298 K) of toluene.

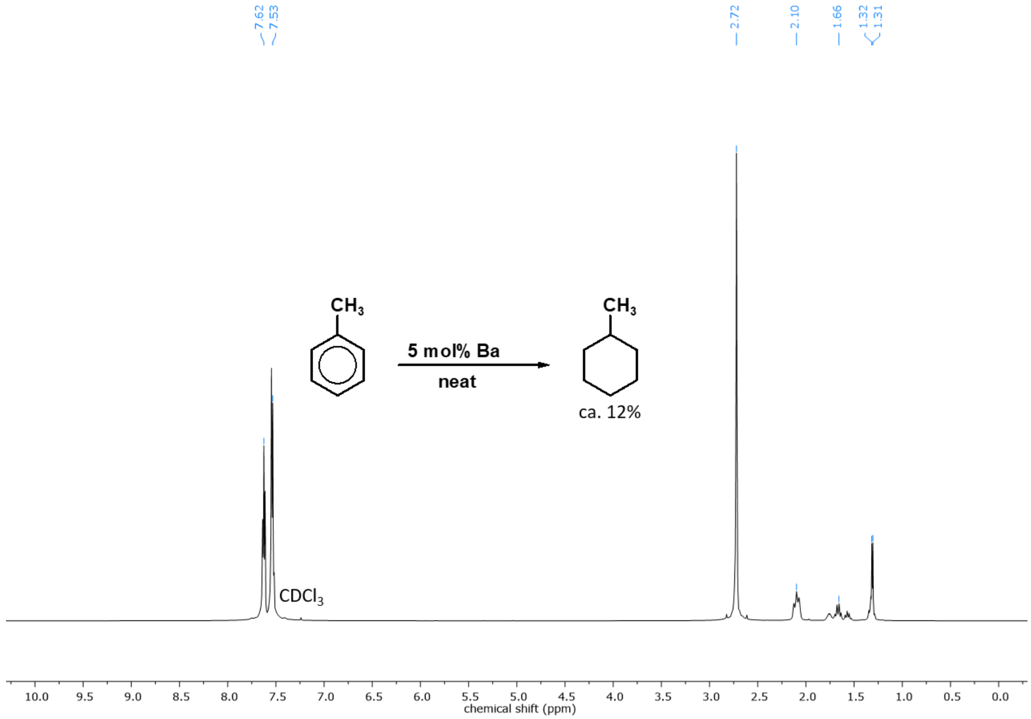

Figure S46. ^{1}H NMR spectrum (600 MHz, $CDCl_3$, 298 K) after catalytic hydrogenation of toluene (neat, 50 bar H_2) with 5 mol% Ba at 150°C for 24 h. Conversion to methylcyclohexane according to GC/MS analysis: 12% (unreacted toluene 88%).

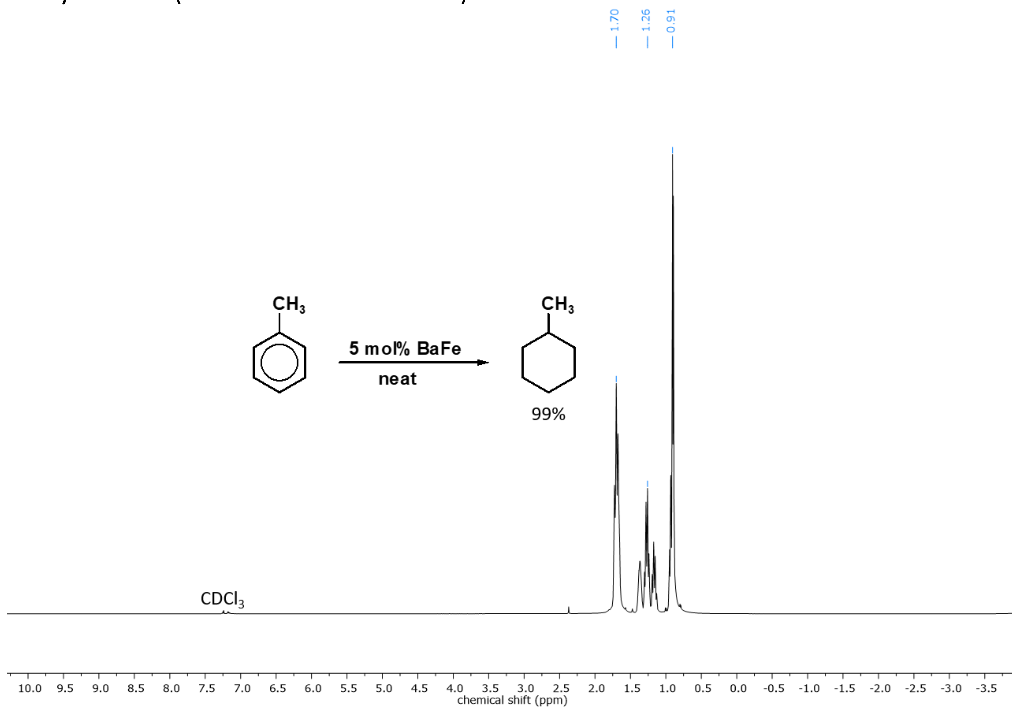

Figure S47. ^{1}H NMR spectrum (600 MHz, $CDCl_3$, 298 K) after catalytic hydrogenation of toluene (neat, 50 bar H_2) with 3 mol% BaFe at 150°C for 3 h. Conversion to methylcyclohexane: 99%.

para-Xylene

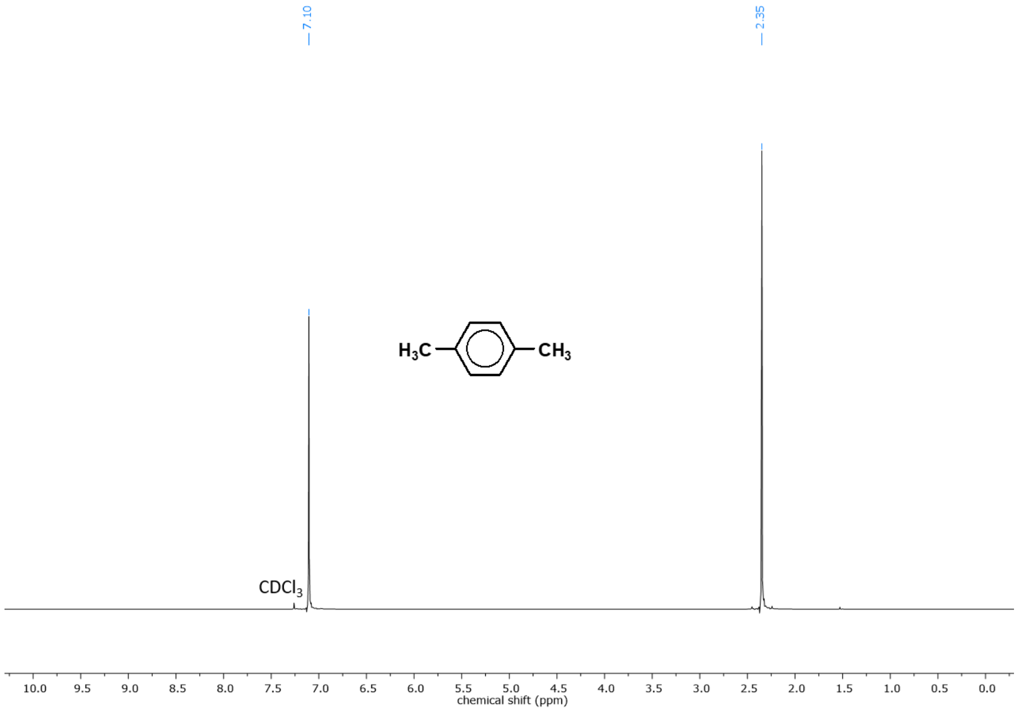

Figure S48. ^{1}H NMR reference spectrum (600 MHz, $CDCl_3$, 298 K) of *para*-xylene.

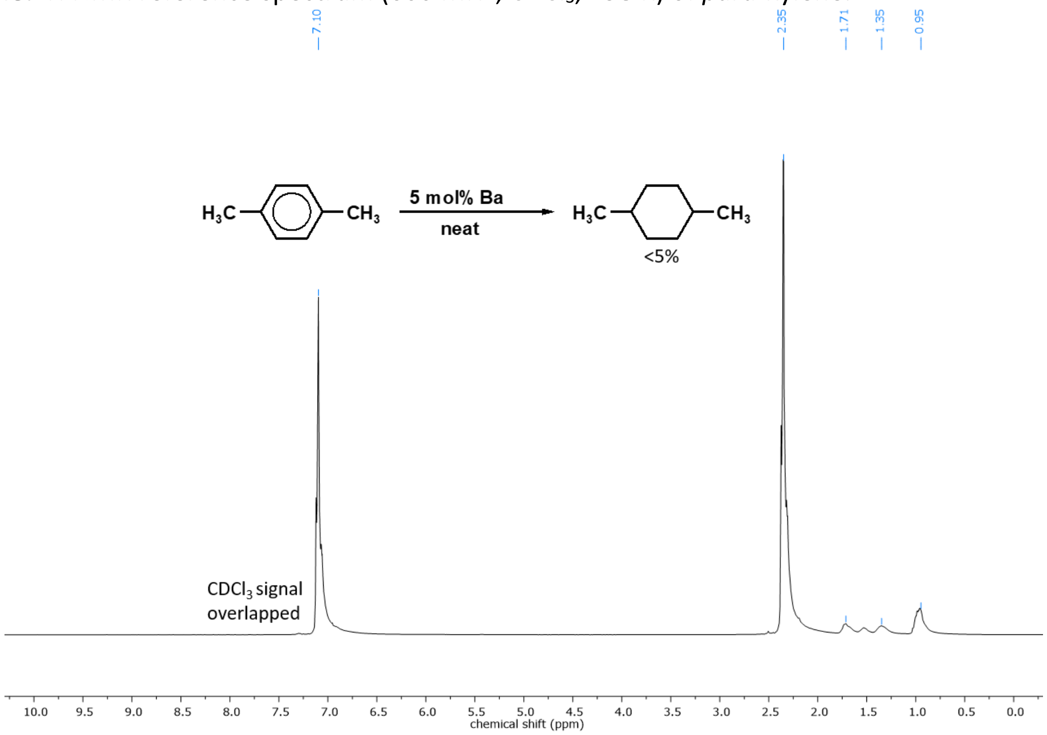

Figure S49. ^{1}H NMR spectrum (600 MHz, $CDCl_3$, 298 K) after catalytic hydrogenation of *para*-xylene (neat, 50 bar H_2) with 5 mol% Ba at 150°C for 24 h. Conversion to 1,4-dimethylcyclohexane was according to GC/MS analysis 4%.

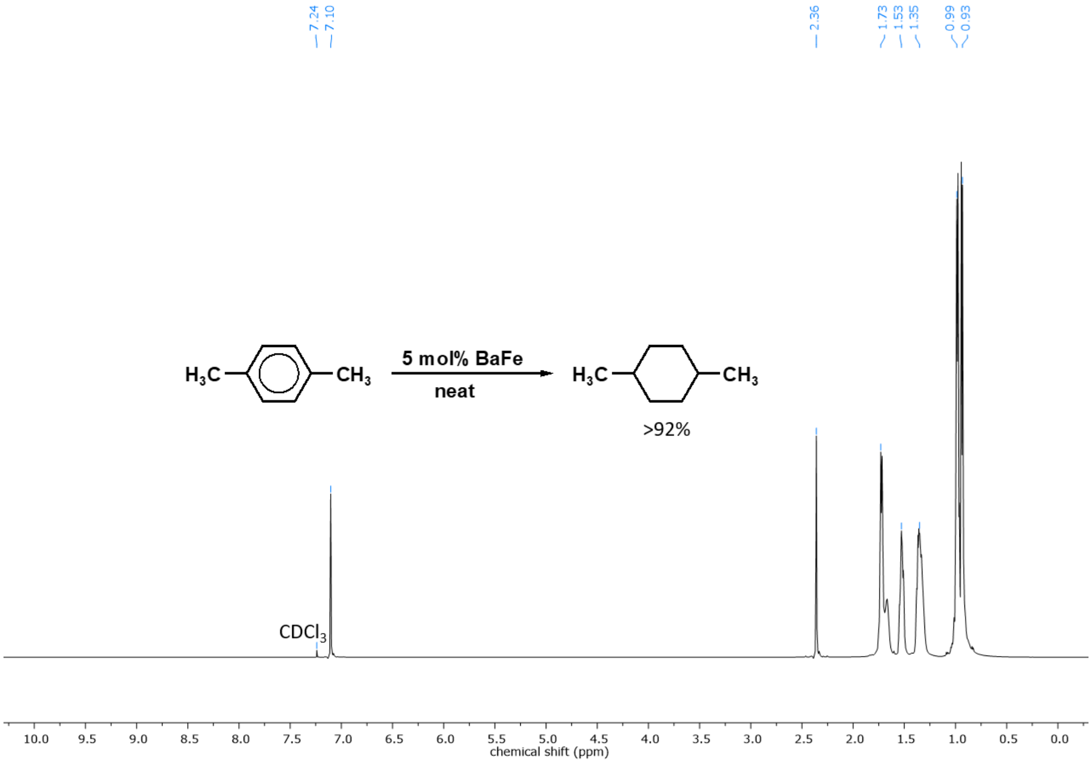

Figure S50. ^{1}H NMR spectrum (600 MHz, $CDCl_3$, 298 K) after catalytic hydrogenation of xylene (neat, 50 bar H_2) with 5 mol% BaFe at 150°C for 6 h. Conversion to 1,4-dimethylcyclohexane: >92%.

Anthracene

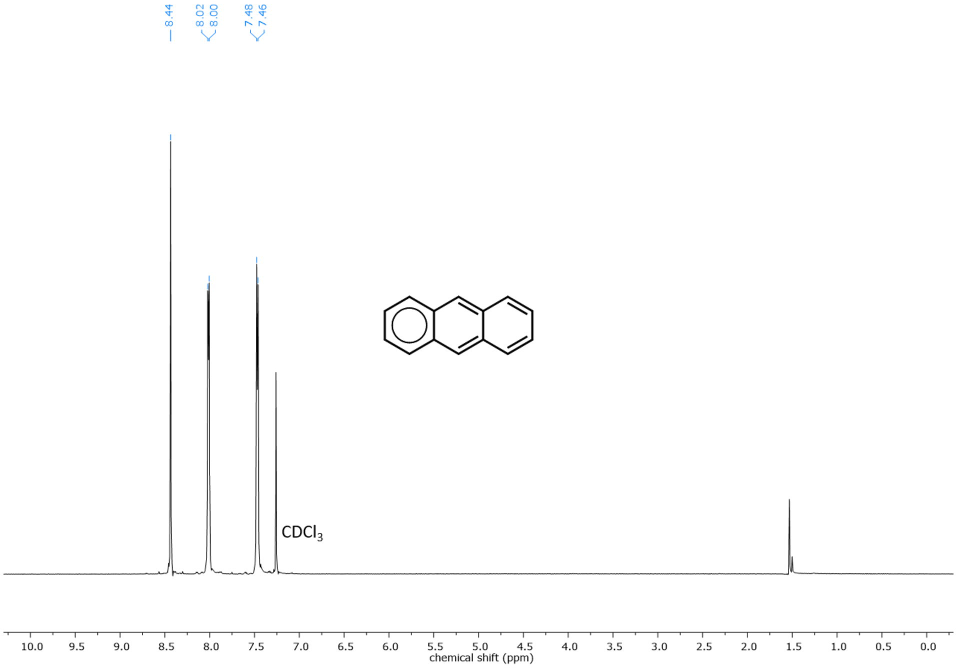

Figure S51. ^{1}H NMR reference spectrum (600 MHz, $CDCl_3$, 298 K) of anthracene.

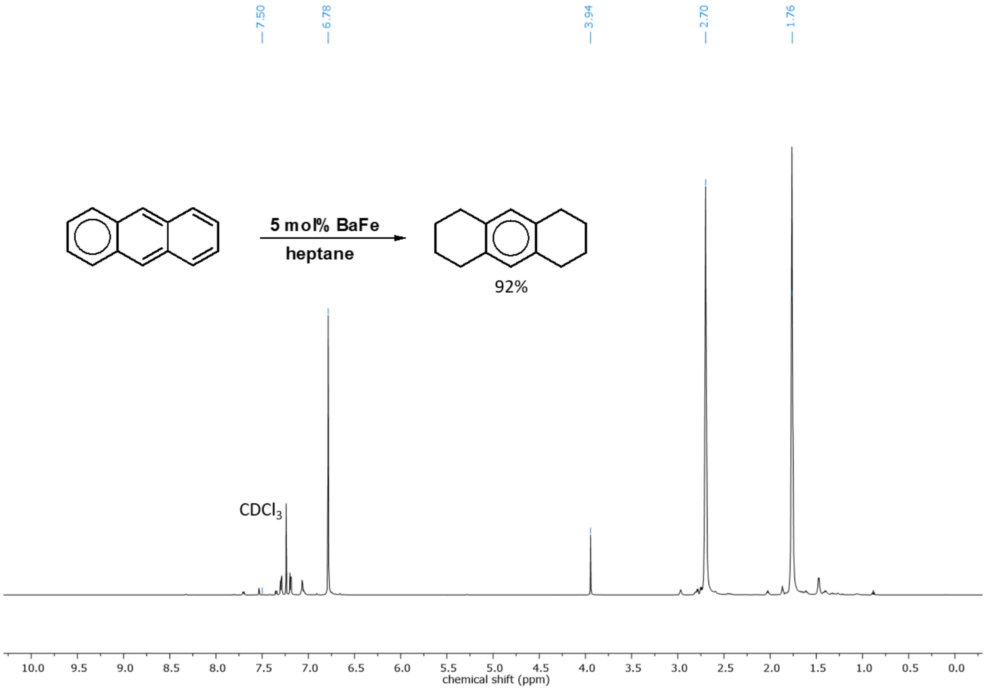

Figure S52. ^{1}H NMR spectrum (600 MHz, $CDCl_3$, 298 K) after catalytic hydrogenation of anthracene (250 mg, 1.5 mmol in 1000 µL *n*-heptane, 50 bar H_2) with 5 mol% BaFe at 150°C for 24 h. Conversion to 1,2,3,4,5,6,7,8-octahydroanthracene was also confirmed by GC/MS analysis (92%). Further analysis revealed the presence of 1,2,3,4,4a,9,9a,10-octahydroanthracene (4.6%), 9,10-dihydroanthracene (2.2%) and 1,2,3,4-tetrahydroanthracene (1.5%).

Naphtalene

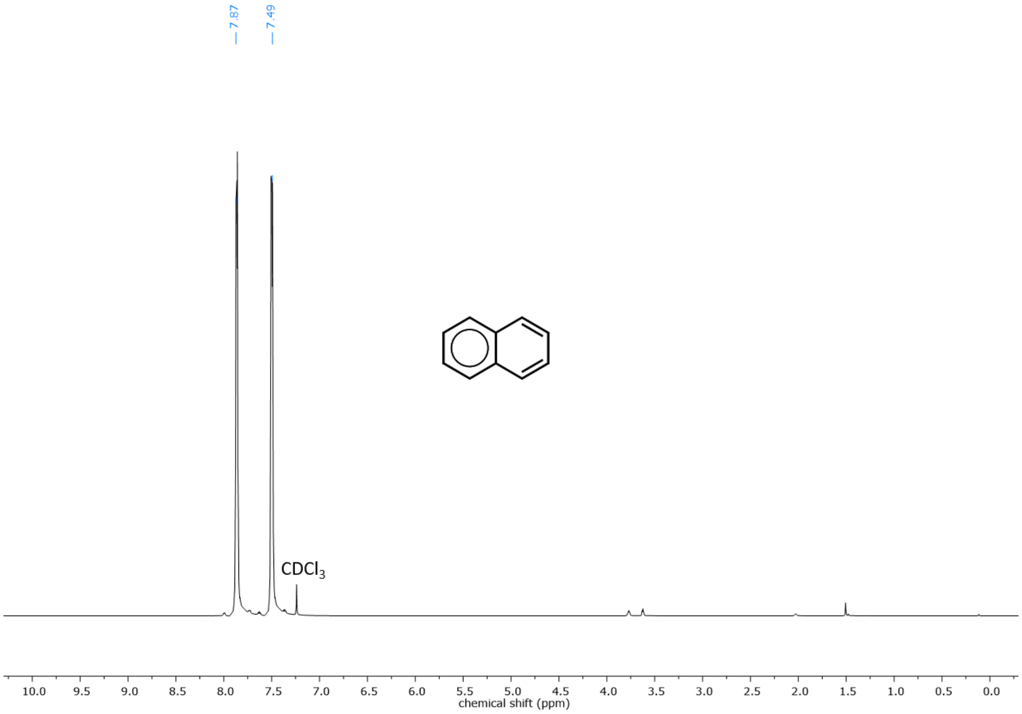

Figure S53. ^{1}H NMR reference spectrum (600 MHz, $CDCl_3$, 298 K) of naphtalene.

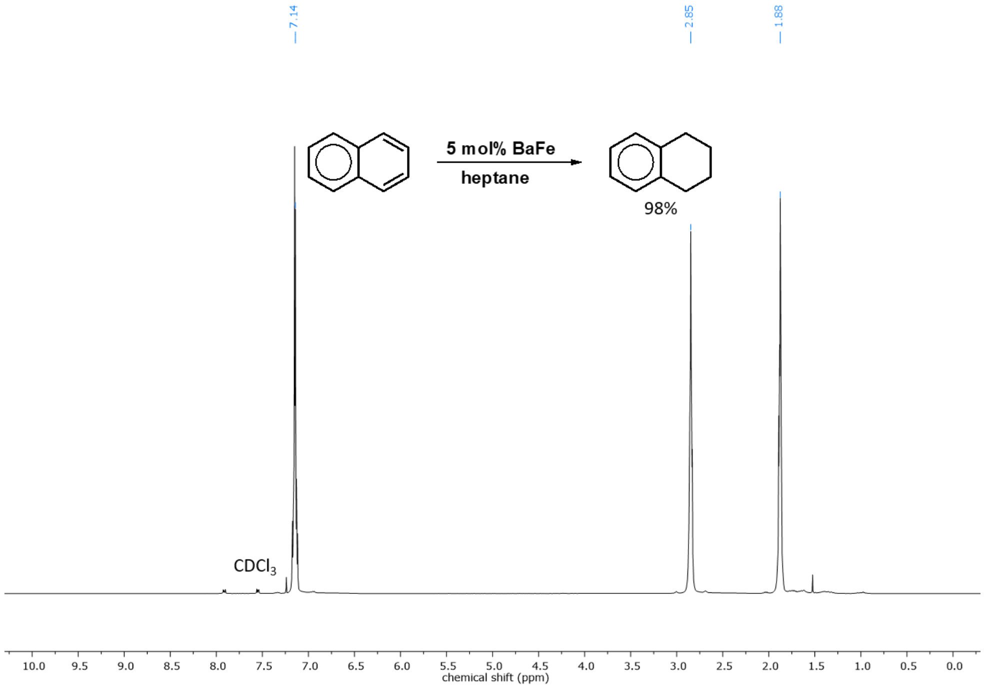

Figure S54. ^{1}H NMR spectrum (600 MHz, $CDCl_3$, 298 K) after catalytic hydrogenation of naphtalene (100 mg, 0.78 mmol in 750 µL *n*-heptane, 20 bar H_2) with 5 mol% BaFe at 120°C for 1 h. Conversion to 1,2,3,4-tetrahydronaphtalene was also confirmed by GC/MS analysis: 98%.

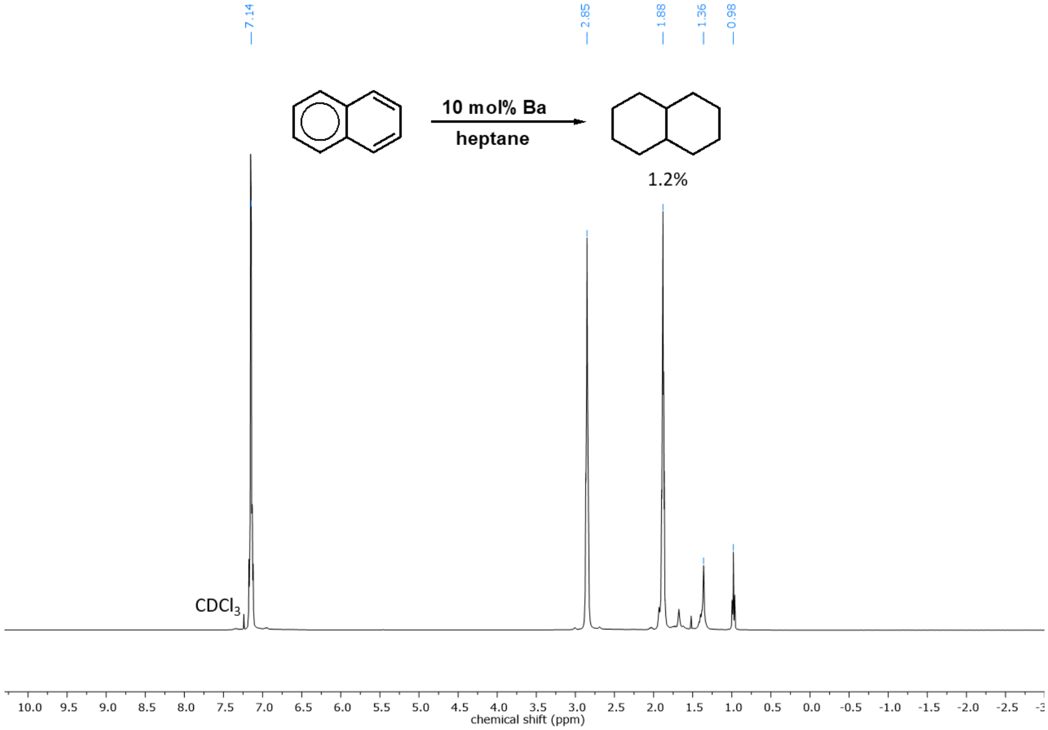

Figure S55. ^{1}H NMR spectrum (600 MHz, $CDCl_3$, 298 K) after catalytic hydrogenation of naphtalene (100 mg, 0.78 mmol in 750 µL *n*-heptane, 50 bar H_2) with 10 mol% Ba at 150°C for 24 h. As a main product (96.3%) conversion to 1,2,3,4-tetrahydronaphtalene was detected by GC/MS and NMR analysis while full hydrogenation of both aromatic rings occurred.

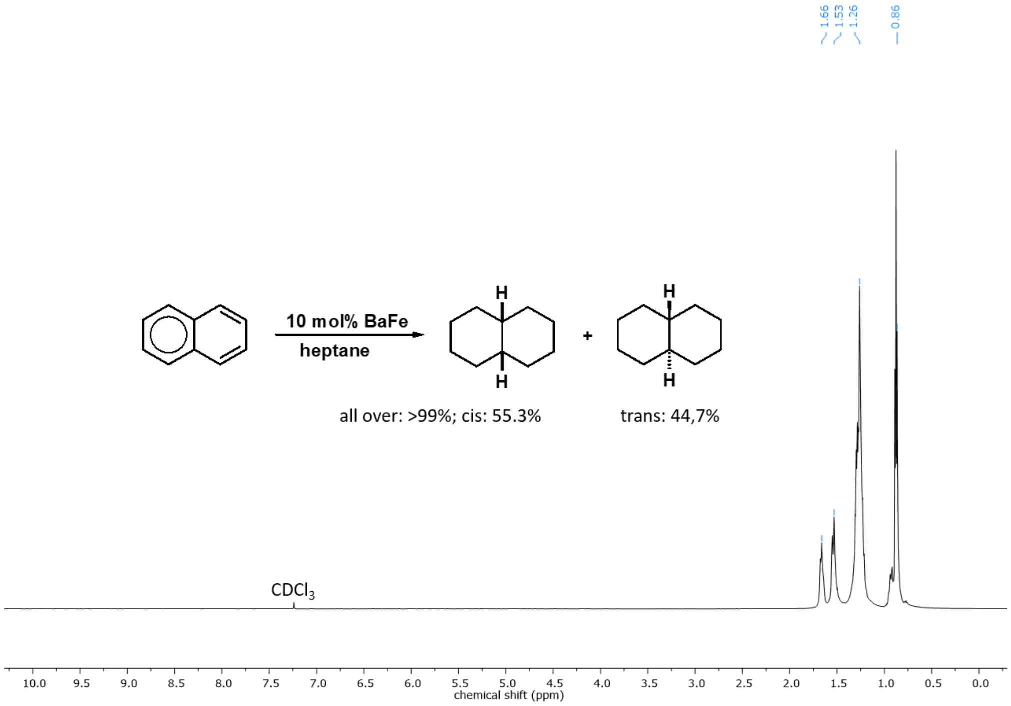

Figure S56. ^{1}H NMR spectrum (600 MHz, $CDCl_3$, 298 K) after catalytic hydrogenation of naphtalene (100 mg, 0.78 mmol in 750 µL *n*-heptane, 50 bar H_2) with 10 mol% BaFe at 150°C for 24 h. As a main product (>99%) conversion to decahydronaphtalene was confirmed by GC/MS and NMR analysis.

Biphenyl

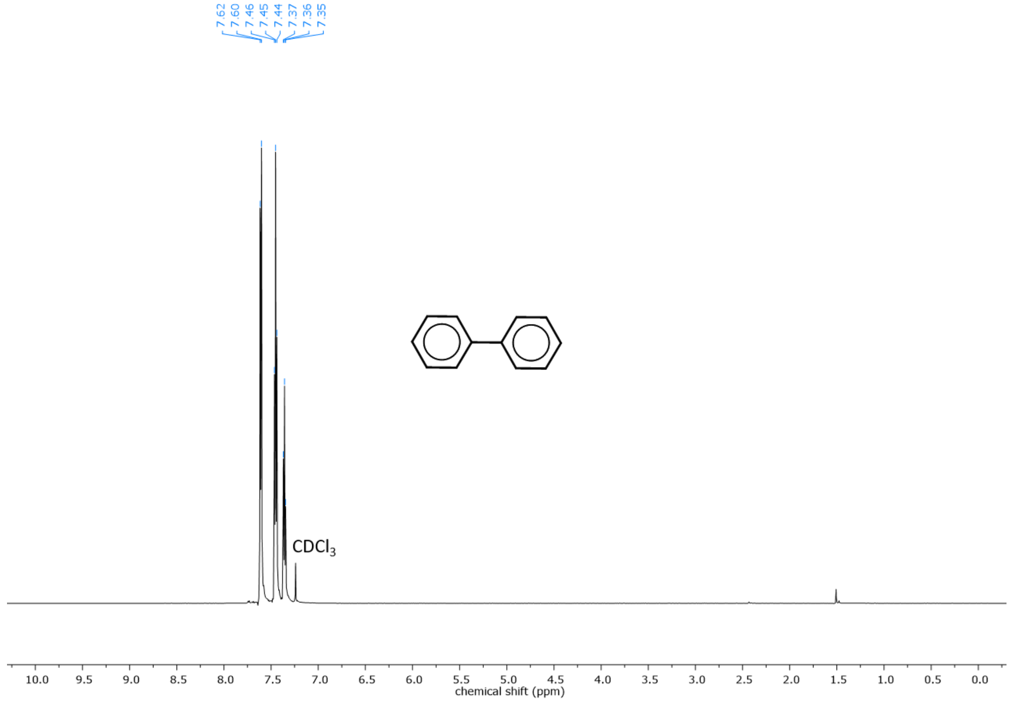

Figure S57. ^{1}H NMR reference spectrum (600 MHz, $CDCl_3$, 298 K) of biphenyl.

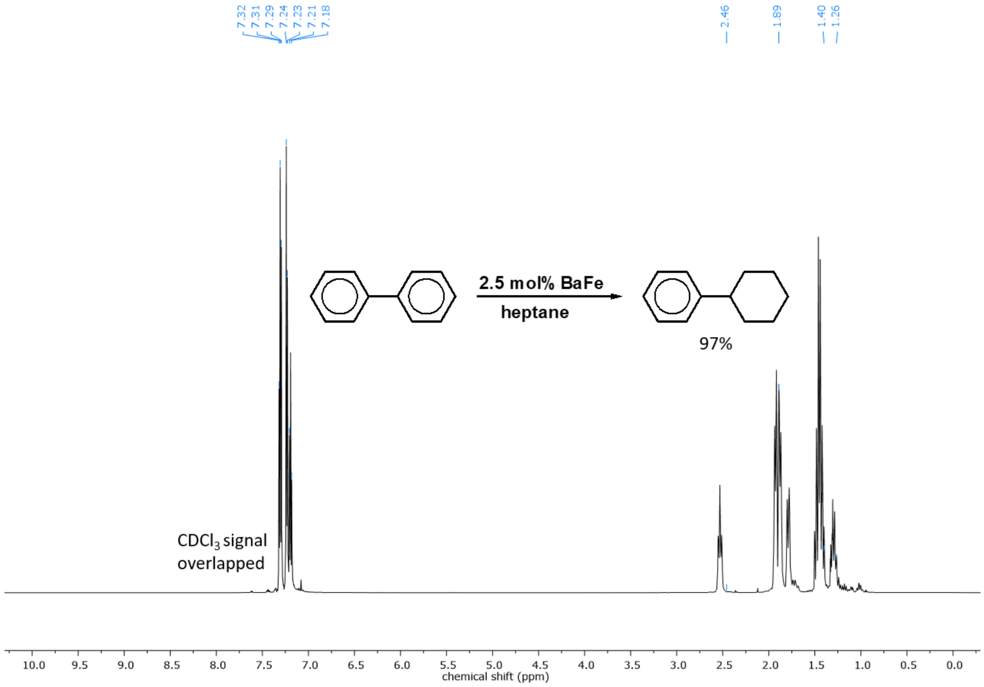

Figure S58. ^{1}H NMR spectrum (600 MHz, $CDCl_3$, 298 K) after catalytic hydrogenation of biphenyl (100 mg, 0.65 mmol in 750 µL *n*-heptane, 20 bar H_2) with 2.5 mol% BaFe at 120°C for 2 h. Conversion (GC/MS) to phenylcyclohexene: 97%.

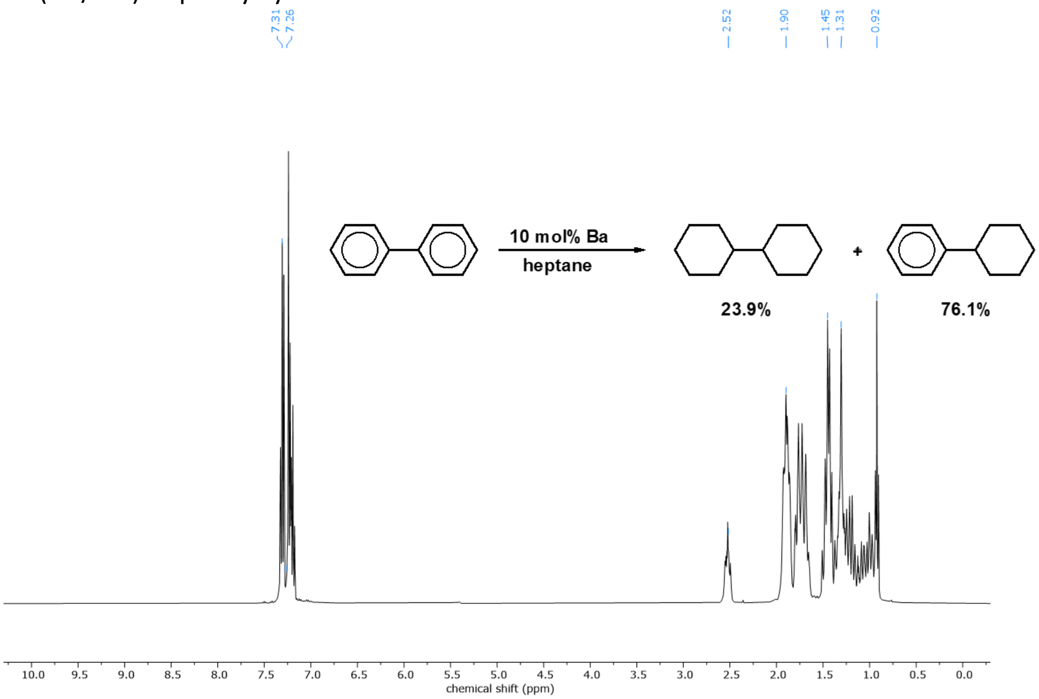

Figure S59. ^{1}H NMR spectrum (600 MHz, $CDCl_3$, 298 K) after catalytic hydrogenation of biphenyl (100 mg, 0.65 mmol in 750 µL *n*-heptane, 50 bar H_2) with 10 mol% Ba at 150°C for 24 h. Conversion (GC/MS) to bicyclohexyl: 23.9%.

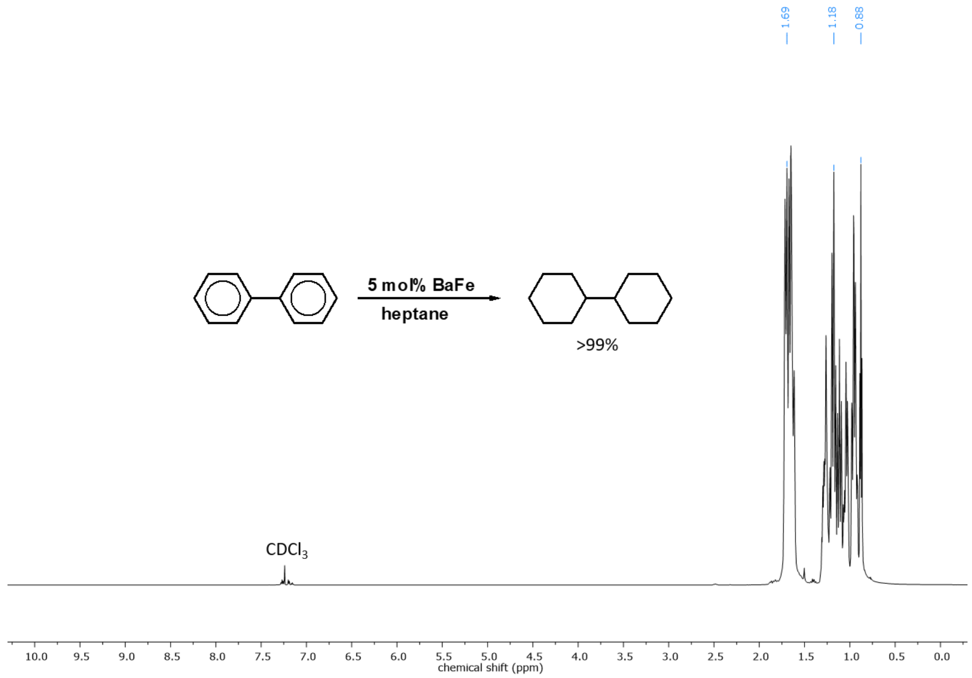

Figure S60. ^{1}H NMR spectrum (600 MHz, $CDCl_3$, 298 K) after catalytic hydrogenation of biphenyl (100 mg, 0.65 mmol in 750 µL n-heptane, 50 bar H_2) with 5 mol% BaFe at 150°C for 6 h. Conversion (GC/MS) to bicyclohexyl: 99%.

Acenaphthylene

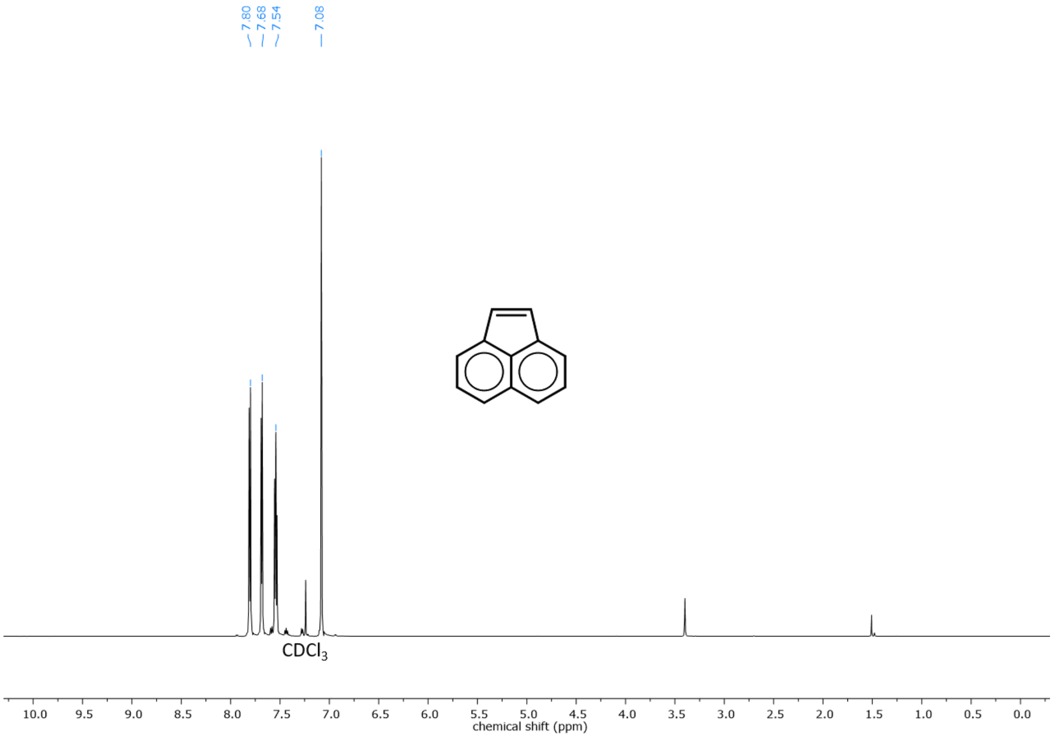

Figure S61. ^{1}H NMR reference spectrum (600 MHz, $CDCl_3$, 298 K) of acenaphthylene.

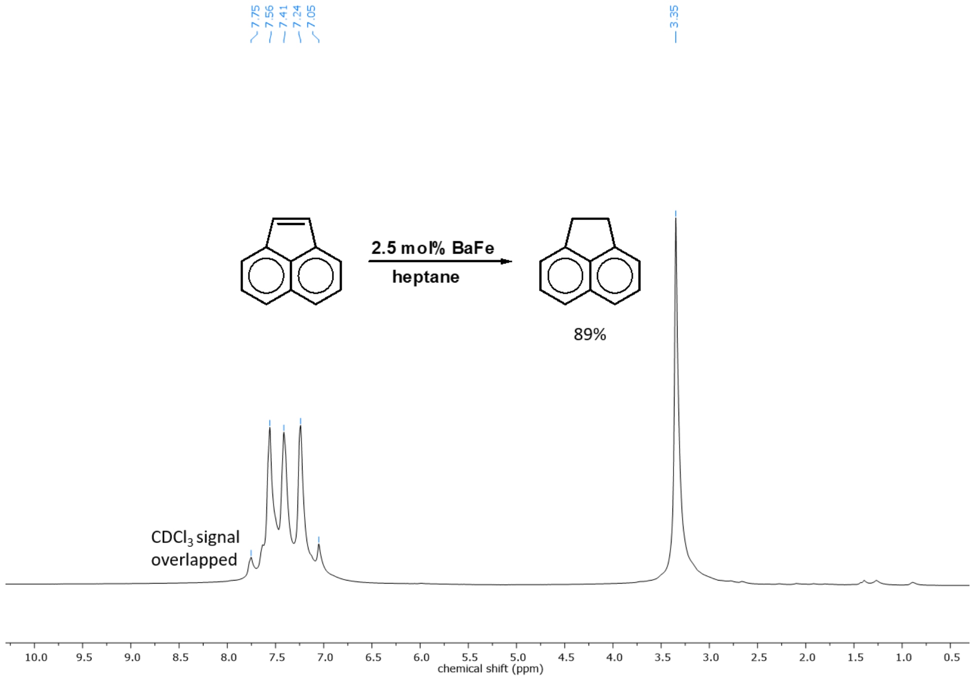

Figure S62. ^{1}H NMR spectrum (600 MHz, $CDCl_3$, 298 K) after catalytic hydrogenation of acenaphthylene (100 mg, 0.66 mmol in 750 µL n-heptane, 12 bar H_2) with 2.5 mol% BaFe at 150°C for 10 min. Conversion (GC/MS) to acenaphthene: 89%. Starting material could also be detected (10%) as well as hexahydroacenaphthylene (1%).

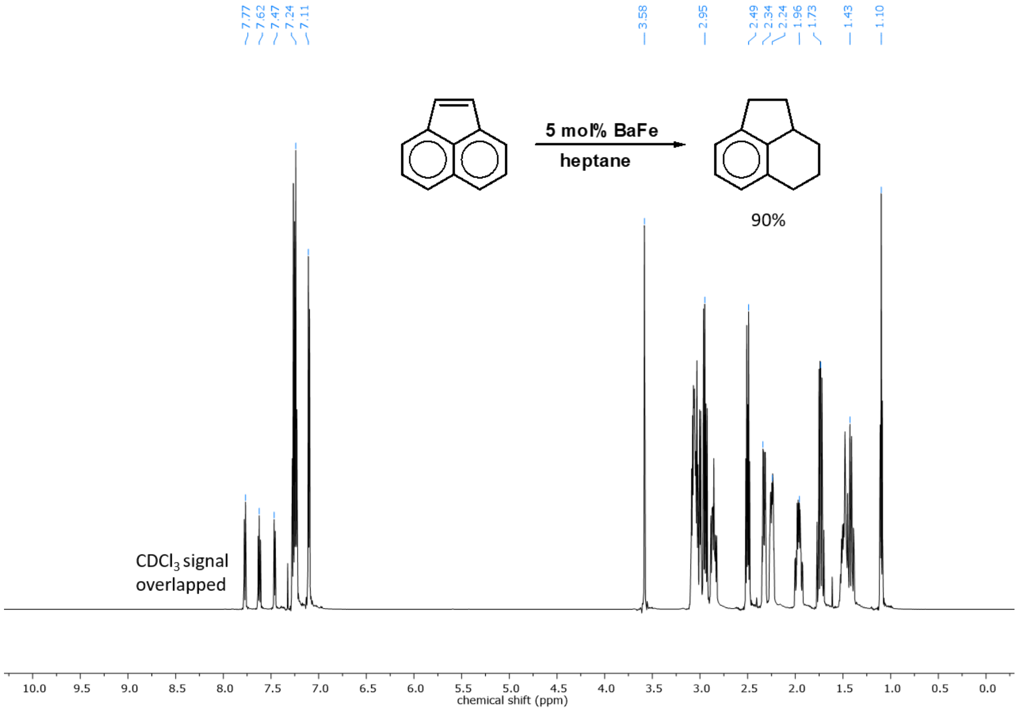

Figure S63. ^{1}H NMR spectrum (600 MHz, $CDCl_3$, 298 K) after catalytic hydrogenation of acenaphthylene (100 mg, 0.66 mmol in 750 µL *n*-heptane, 20 bar H_2) with 5 mol% BaFe at 120°C for 1 h. Conversion (GC/MS) to acenaphthene: 10%. Hexahydroacenaphthylene (90%) is the main component.

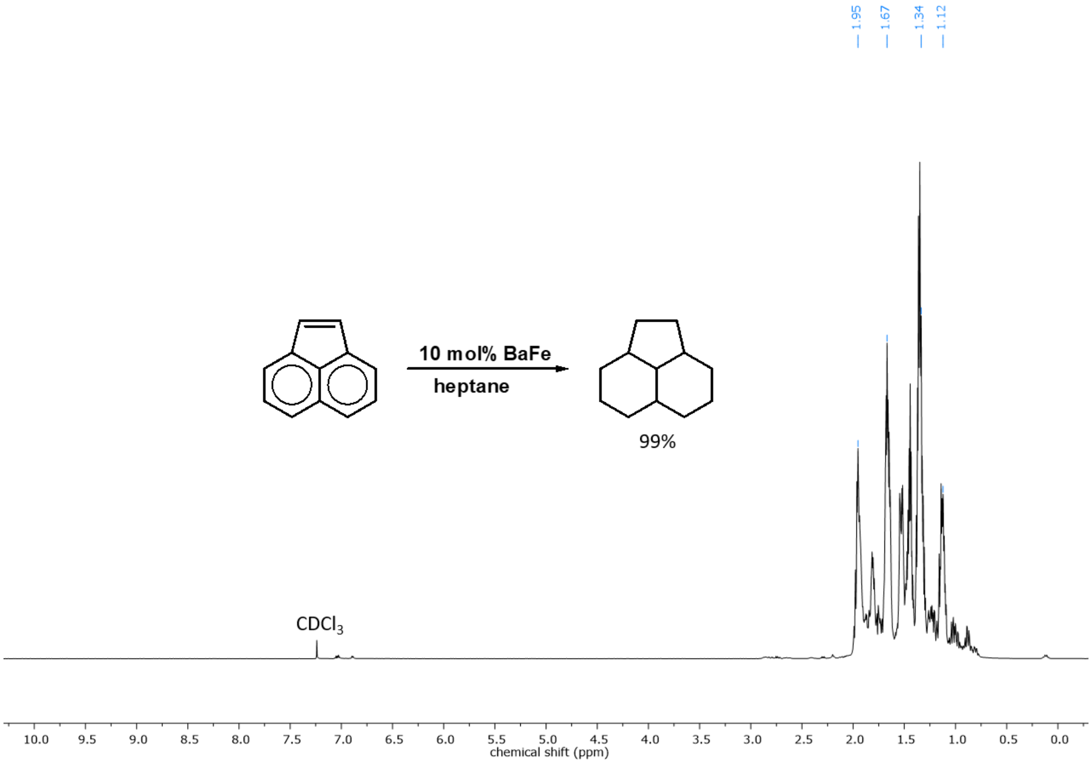

Figure S64. ^{1}H NMR spectrum (600 MHz, $CDCl_3$, 298 K) after catalytic hydrogenation of acenaphthylene (100 mg, 0.66 mmol in 750 µL n-heptane, 50 bar H_2) with 10 mol% BaFe at 150°C for 38 h. Conversion (GC-MS) to dodecahydroacenaphthylene: 99%.

Diphenylacetylene

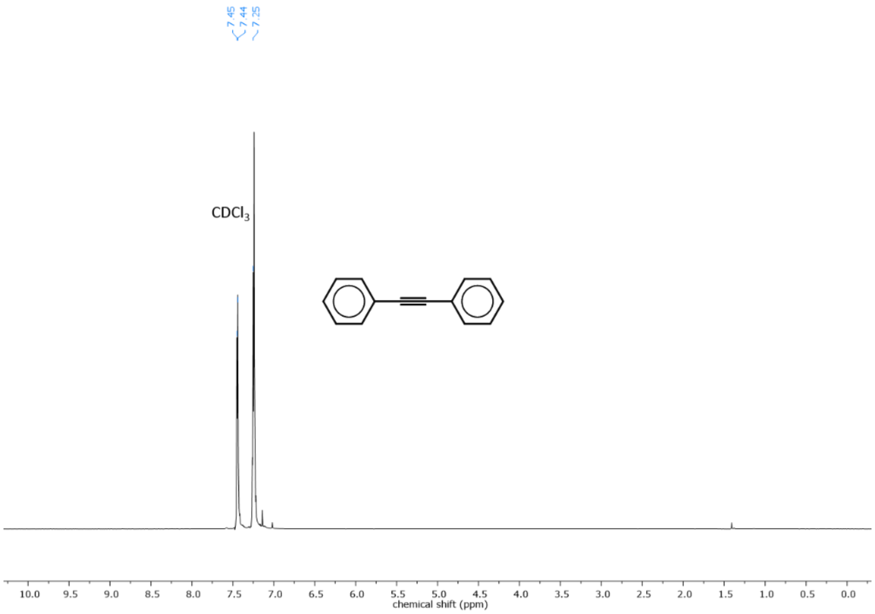

Figure S65. ^{1}H NMR reference spectrum (600 MHz, $CDCl_3$, 298 K) of diphenylacetylene.

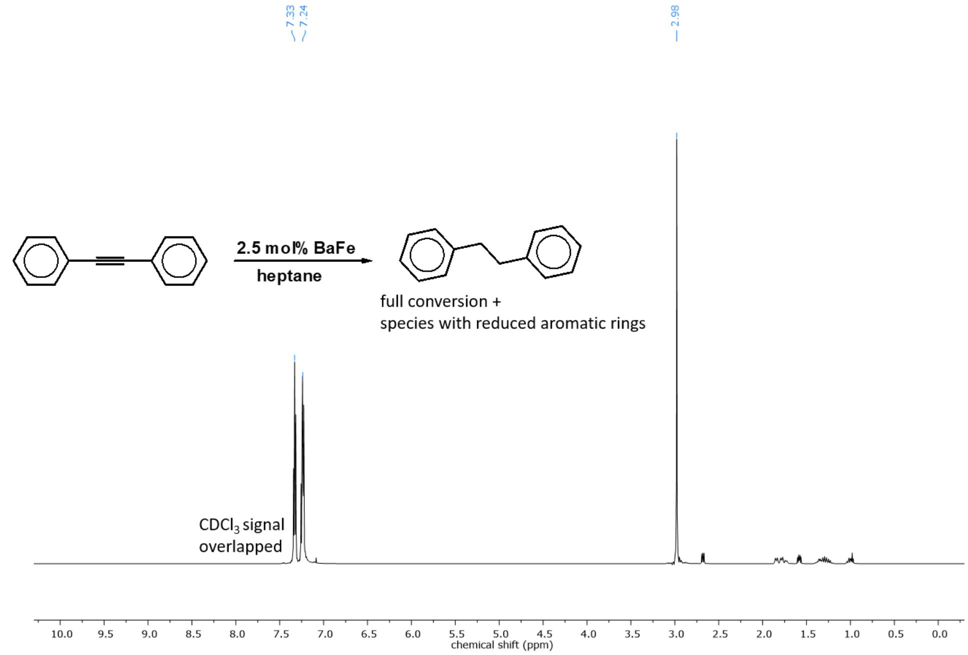

Figure S66. ${}^{1}H$ NMR spectrum (600 MHz, $CDCl_3$, 298 K) after catalytic hydrogenation of diphenylacetylene (200 mg, 1.12 mmol in 750 µL *n*-heptane, 12 bar H_2) with 2.5 mol% BaFe at 120°C for 1 h. Full conversion to 1,2-diphenylethane can be detected via NMR analysis which also reveals the presence of traces of phenyl ring reduction.

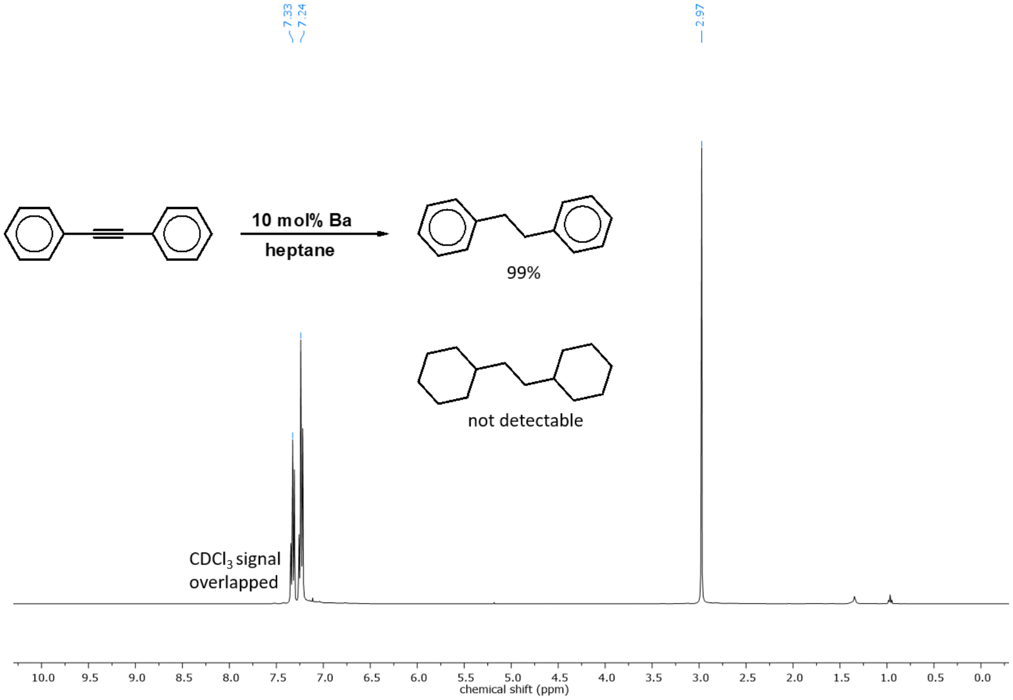

Figure S67. ${}^{1}H$ NMR spectrum (600 MHz, $CDCl_3$, 298 K) after catalytic hydrogenation of diphenylacetylene under forcing conditions (200 mg, 1.12 mmol in 750 µL *n*-heptane, 50 bar H_2) with 10 mol% Ba at 150°C for 24 h. NMR analysis reveals the presence of a sole product: 1,2-diphenylethane. Phenyl ring reduction is not observed.

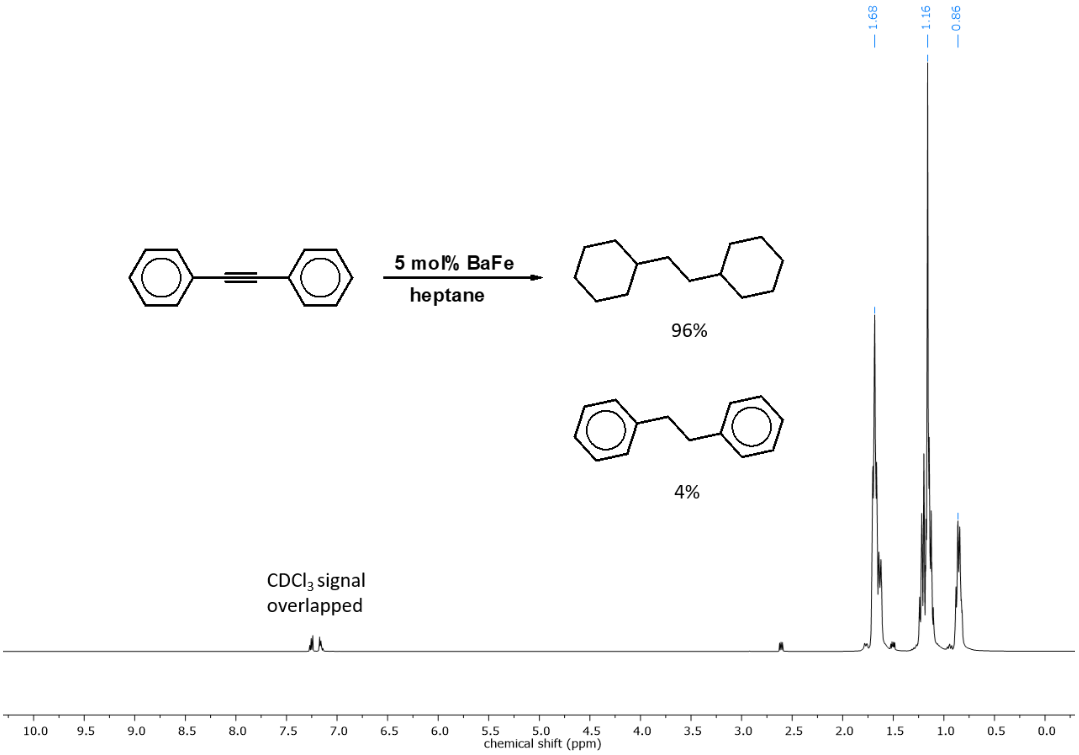

Figure S68. ^{1}H NMR spectrum (600 MHz, $CDCl_3$, 298 K) after catalytic hydrogenation of diphenylacetylene under forcing conditions (200 mg, 1.12 mmol in 750 µL *n*-heptane, 50 bar H_2) with 5 mol% Ba at 150°C for 24 h. Conversion (GC/MS) to 1,2-dicyclohexylethane: 96%.

3-Hexyne

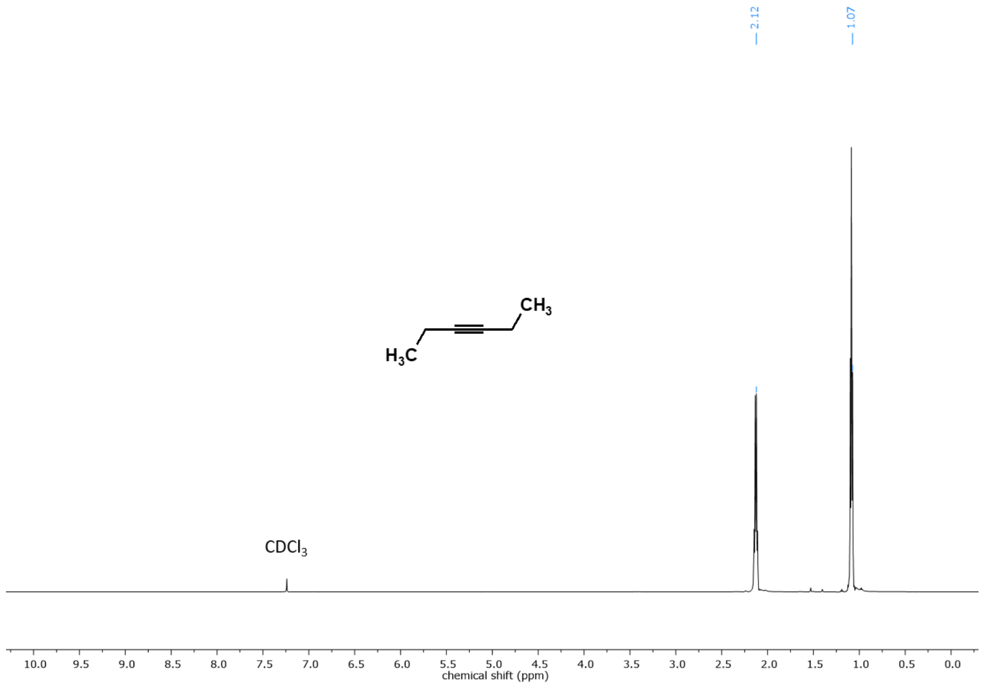

Figure S69. ^{1}H NMR reference spectrum (600 MHz, $CDCl_3$, 298 K) of 3-hexyne.

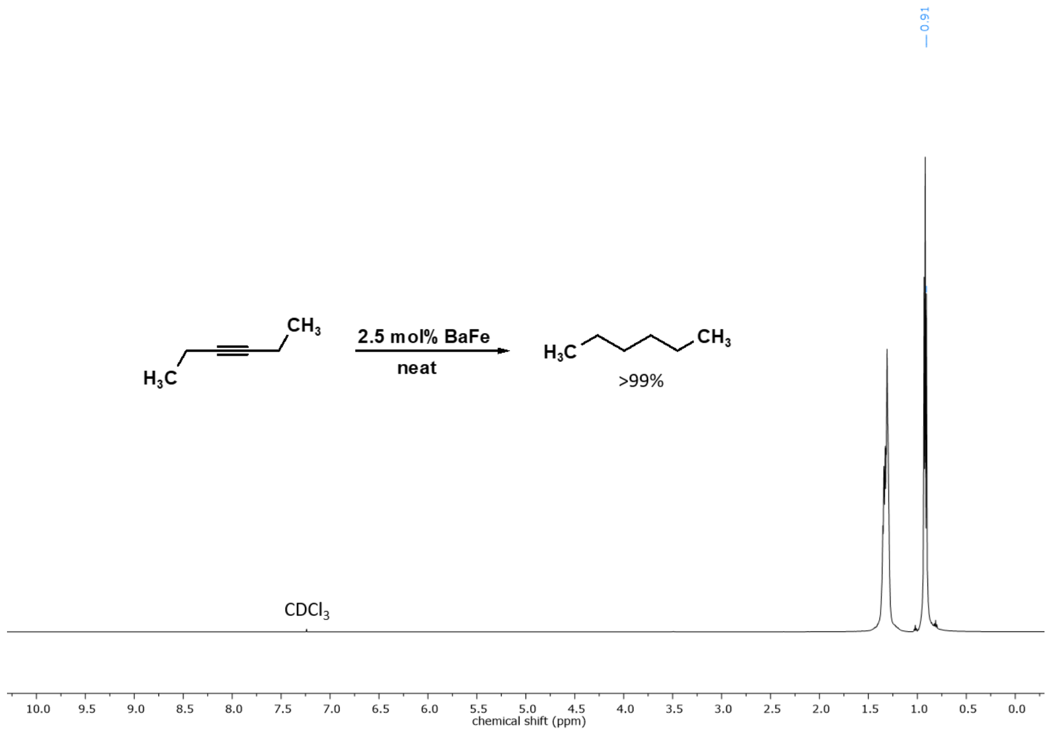

Figure S70. ^{1}H NMR spectrum (600 MHz, $CDCl_3$, 298 K) after catalytic hydrogenation of 3-hexyne (neat, 20 bar H_2) with 2.5 mol% BaFe at 120 °C for 1 h. Conversion to hexane: >99%.

***N*-Benzylidene-*tert*-butylimine**

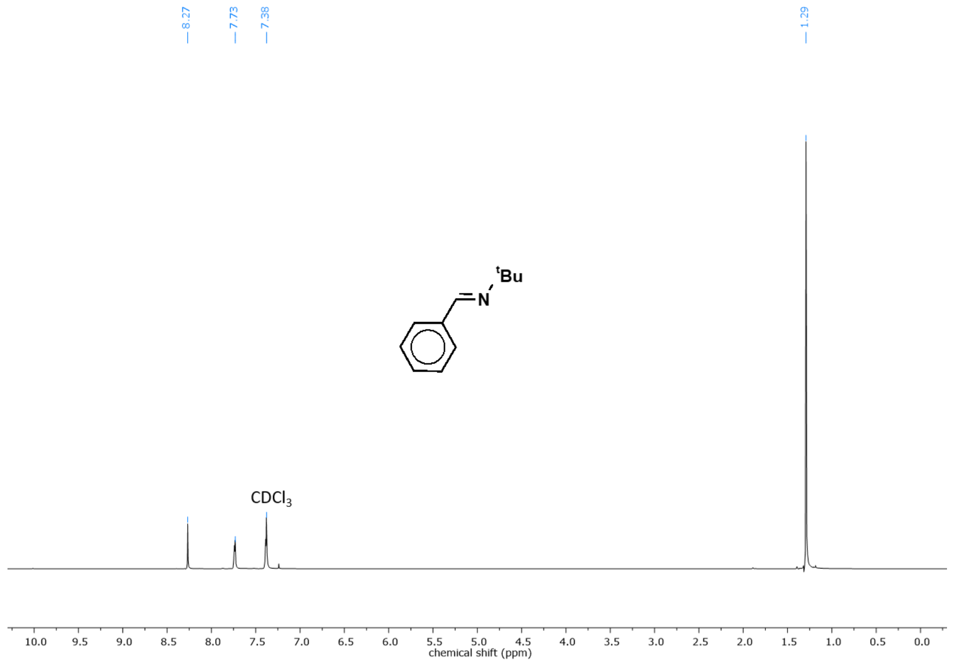

Figure S71. ^{1}H NMR reference spectrum (600 MHz, $CDCl_3$, 298 K) of *N*-benzylidene-tert-butylimine.

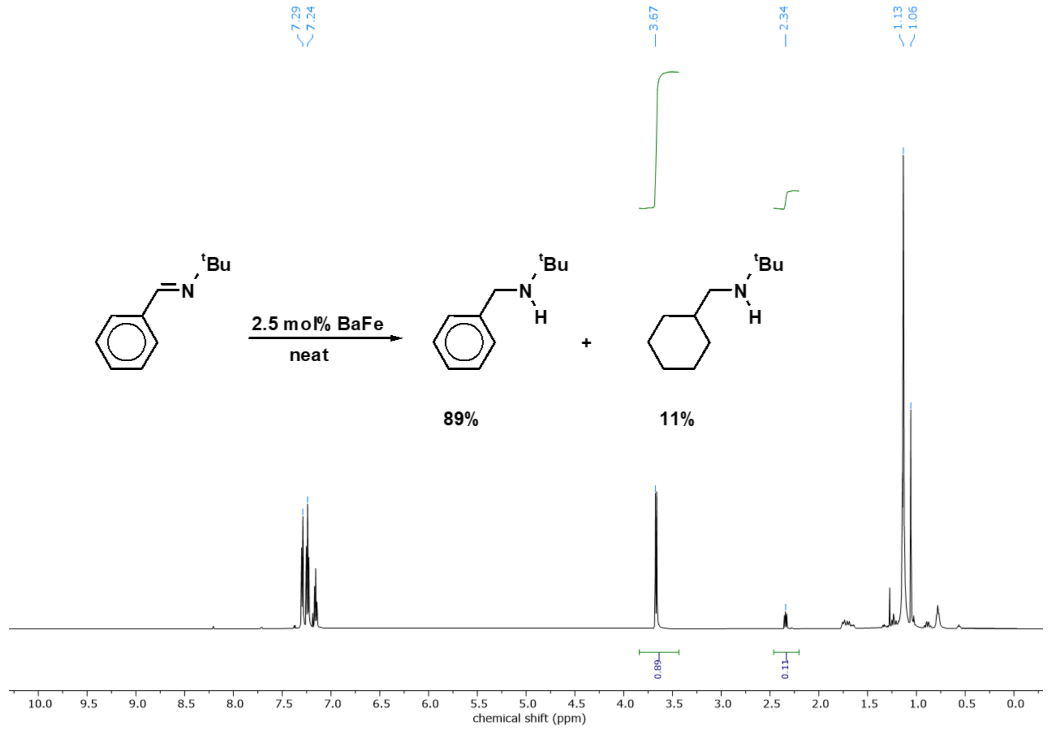

Figure S72. ^{1}H NMR spectrum (600 MHz, $CDCl_3$, 298 K) after catalytic hydrogenation of *N*-benzylidene-tert-butyl-amine (neat, 12 bar H_2) with 2.5 mol% BaFe at 120°C for 2 h. NMR analysis confirms full conversion to benzyl-tert-butyl-amine. The side-product with phenyl ring reduction is also present.

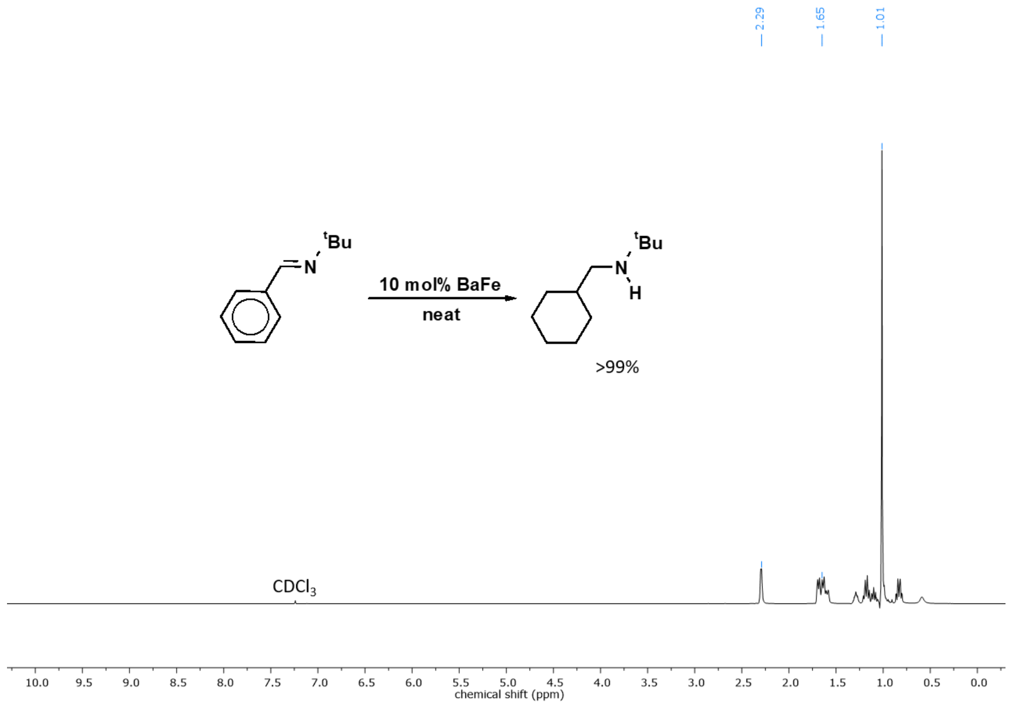

Figure S73. ^{1}H NMR spectrum (600 MHz, $CDCl_3$, 298 K) after catalytic hydrogenation of *N*-benzylidene-tert-butyl-amine (neat, 50 bar H_2) with 10 mol% BaFe at 150°C for 24 h. NMR analysis confirms full conversion to cyclohexylmethanamine.

N-(diphenylmethylene)aniline

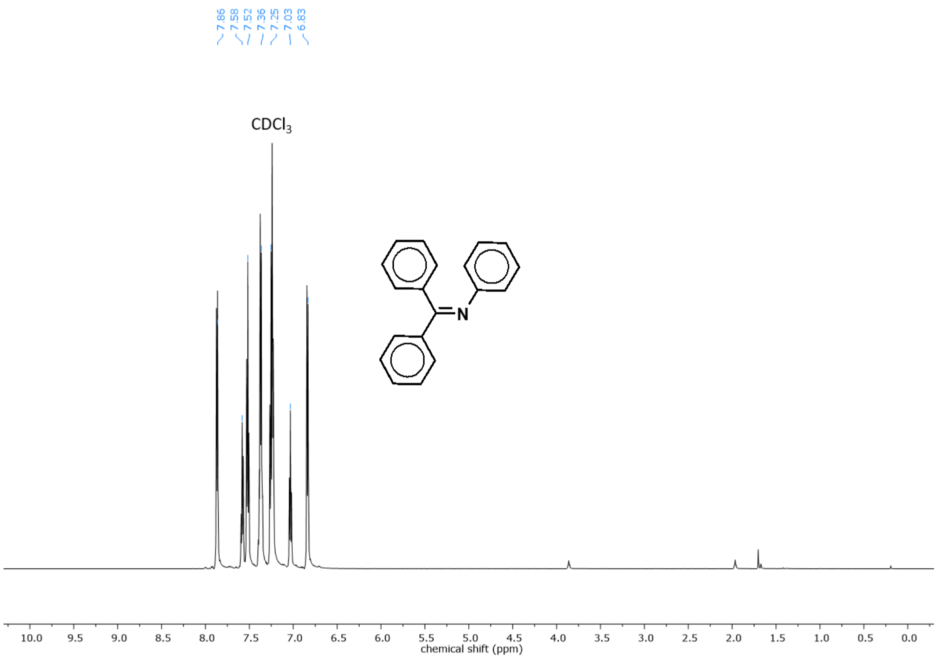

Figure S74. ^{1}H NMR reference spectrum (600 MHz, CDCl$_3$, 298 K) of *N*-(diphenylmethylene)aniline.

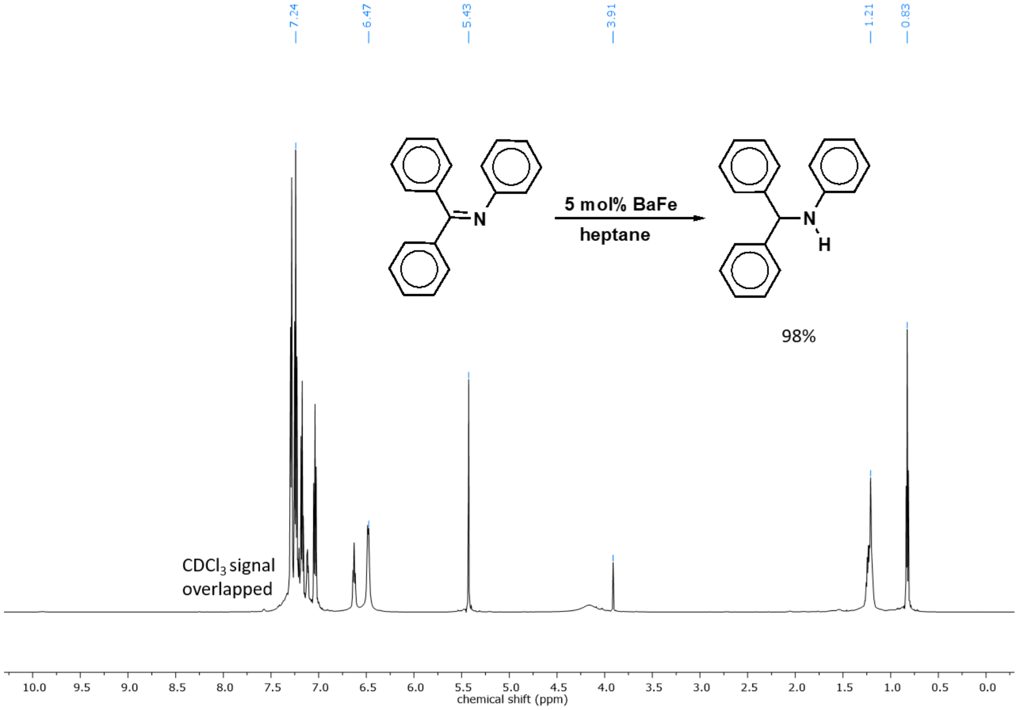

Figure S75. ^{1}H NMR spectrum (600 MHz, CDCl$_3$, 298 K) after catalytic hydrogenation of *N*-(diphenylmethylene)aniline (200 mg, 0.78 mmol in 750 µL heptane, 50 bar H_2) with 7.5 mol% BaFe at 150°C for 24 h. The crude product was examined by NMR and GC/MS analysis which confirmed reduction of the double bond (98%).

Quinoline

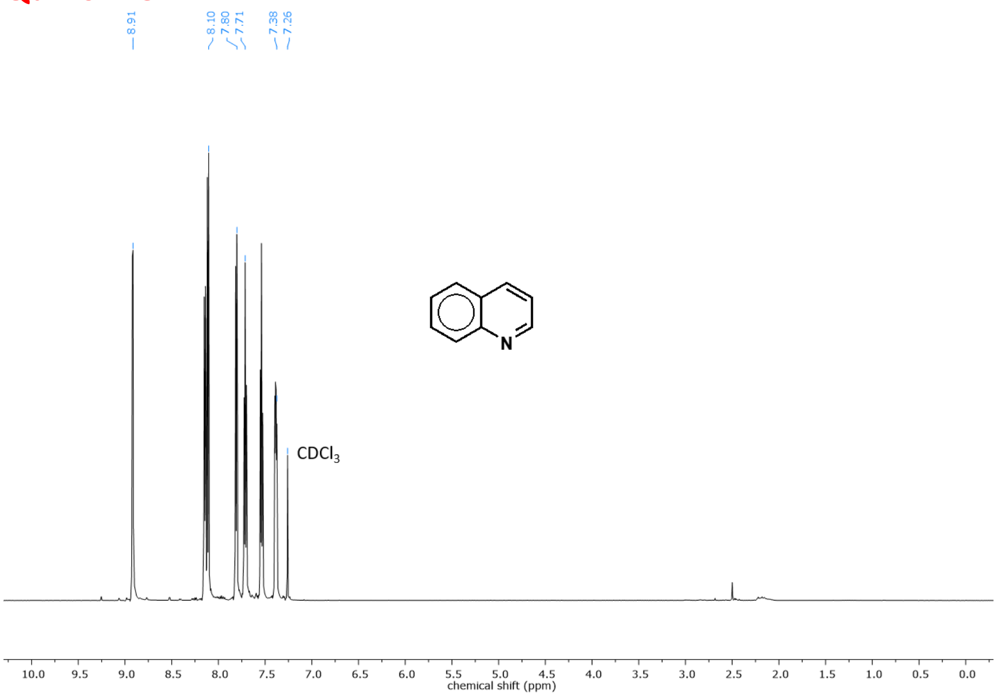

Figure S76. ^{1}H NMR reference spectrum (600 MHz, $CDCl_3$, 298 K) of quinoline.

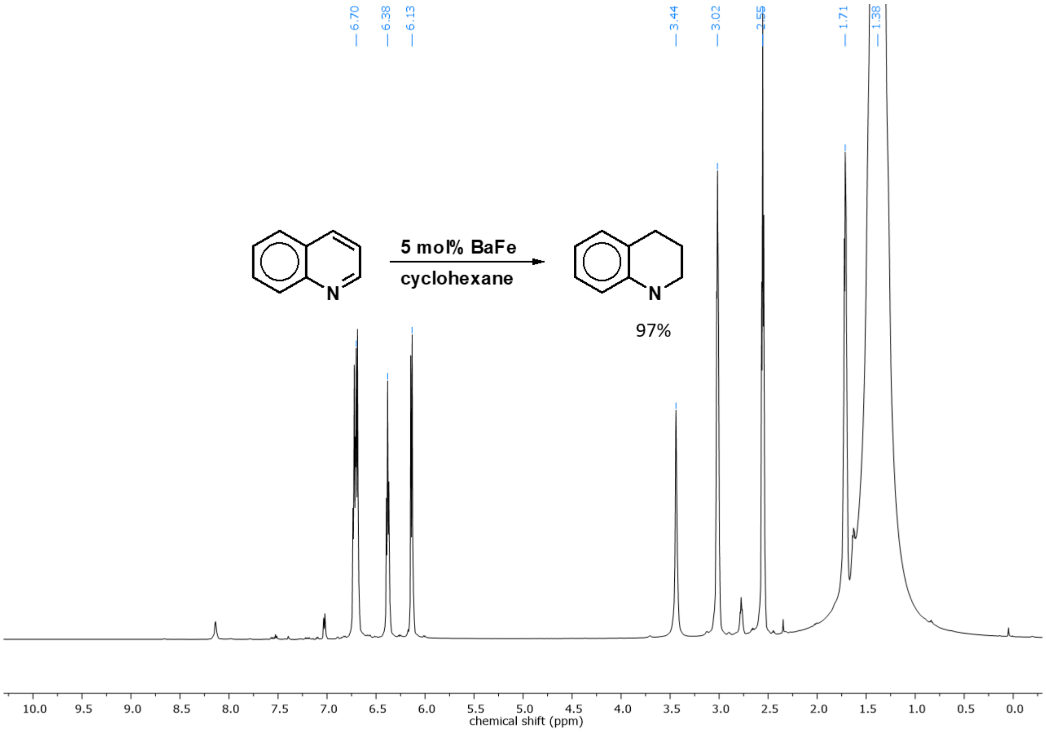

Figure S77. ^{1}H NMR spectrum (600 MHz, $CDCl_3$, 298 K) after catalytic hydrogenation of quinoline (118 µL, 1.00 mmol) in cyclohexane (882 µL) with 5 mol% BaFe (120°C, 20 bar H_2). The conversion after 11 h is 97% and after 12 h is 99% (GC-MS).

References

[1] R. Wang, M. Ma, X. Gong, G. B. Panetti, X. Fan, P.J. Walsh, Visible-Light-Mediated Umpolung Reactivity of Imines: Ketimine Reductions with Cy_2NMe and Water. *Org. Letters* **2018**, *20*, 2433-2436.

[2] P. L. Timms, Transition metal vapors in chemical synthesis. The direct preparation of dibenzene chromium as an undergraduate experiment. *J. Chem. Educ.* **1972**, *49*, 782-784

[3] U. Zenneck, Die Chemie freier Metall-Atome. *Chemie in unserer Zeit* **1993**, *27*, 208-219.

[4] U. Zenneck, W. Frank, Bis (η^2-ethene)(η^6-toluene)iron. *Angew. Chem. Int. Ed. Engl.* **1986**, *25*, 831-833.

[5] C. Batalli-Cosmovici, β-Modifikation des Bariums bei im Vakuum zerstäubten Partikeln. *Z. Naturforsch. - Sect. A J. Phys. Sci.* **1969**, *24*, 677.

[6] O. Kubaschewski in IRON - Binary Phase Diagrams, Springer-Verlag Berlin Heidelberg, Germany, **1982**.

[7] M. Descostes, F. Mercier, N. Thromat, C. Beaucaire, M. Gautier-Soyer, Use of XPS in the determination of chemical environment and oxidation state of iron and sulfur samples: constitution of a data basis in binding energies for Fe and S reference compounds and applications to the evidence of surface species of an oxidized pyrite in a carbonate medium. *Appl. Surf. Sci.* **2000**, *165*, 288-302.

[8] O. Karslıoğlu, L. Trotochaud, I. Zegkinoglou, H. Bluhm, X-Ray Spectroscopic Characterization of BaO, $Ba(OH)_2$, $BaCO_3$, and $Ba(NO_3)_2$. *J. Electron Spectros. Relat. Phenomena* 2018, 225, 55–61.

[9] P. Stegner, C. Färber, U. Zenneck, C. Knüpfer, J. Eyselein, M. Wiesinger, S. Harder, Metallic Barium: A Versatile and Efficient Hydrogenation Catalyst. *Angew. Chem. Int. Ed.* **2021**, *60*, 4252-4258.

[10] S. Kozuch, J. M. L. Martin, "Turning Over" Definitions in Catalytic Cycles, *ACS Catal.* **2012**, *2*, 2787-2794.

[11] D. Broadbent, D. Dollimore, J. Dollimore, The Thermal Decomposition of Oxalates. Part IX.[1] The Thermal Decomposition of the Oxalate Complexes of Iron. *J. Chem. Soc. A* **1967**, 451-454.

[12] D. Gärtner, S. Sandl, A. Jacobi von Wangelin, Homogeneous vs. heterogeneous: mechanistic insights into iron group metal-catalyzed reductions from poisoning experiments. *Catal. Sci. Technol.* **2020**, *10*, 3502-3514.

[13] B. A. Suvorov, Intramolecular electronic interaction in a series of organosilicon compounds. *Russ. J. Gen. Chem.* **2006**, *76*, 1401-1406.
